# Map and model—moving from observation to prediction in toxicogenomics

**DOI:** 10.1093/gigascience/giz057

**Published:** 2019-05-29

**Authors:** Andreas Schüttler, Rolf Altenburger, Madeleine Ammar, Marcella Bader-Blukott, Gianina Jakobs, Johanna Knapp, Janet Krüger, Kristin Reiche, Gi-Mick Wu, Wibke Busch

**Affiliations:** 1 Department Bioanalytical Ecotoxicology, Helmholtz-Centre for Environmental Research – UFZ, Permoserstr. 15, 04318 Leipzig, Germany; 2 Institute for Environmental Research, RWTH Aachen, Worringerweg 1, 52074 Aachen, Germany; 3 Bioinformatics Unit, Department of Diagnostics, Fraunhofer Institute for Cell Therapy and Immunology, Perlickstr. 1, 04103 Leipzig, Germany; 4 DEVELOP, Helmholtz-Centre for Environmental Research – UFZ, Permoserstr. 15, 04318 Leipzig, Germany

**Keywords:** risk assessment, environmental monitoring, adverse outcome pathway, mode of action, ’omics time course, dose response, machine learning, diuron, diclofenac, naproxen

## Abstract

**Background:**

Chemicals induce compound-specific changes in the transcriptome of an organism (toxicogenomic fingerprints). This provides potential insights about the cellular or physiological responses to chemical exposure and adverse effects, which is needed in assessment of chemical-related hazards or environmental health. In this regard, comparison or connection of different experiments becomes important when interpreting toxicogenomic experiments. Owing to lack of capturing response dynamics, comparability is often limited. In this study, we aim to overcome these constraints.

**Results:**

We developed an experimental design and bioinformatic analysis strategy to infer time- and concentration-resolved toxicogenomic fingerprints. We projected the fingerprints to a universal coordinate system (toxicogenomic universe) based on a self-organizing map of toxicogenomic data retrieved from public databases. Genes clustering together in regions of the map indicate functional relation due to co-expression under chemical exposure. To allow for quantitative description and extrapolation of the gene expression responses we developed a time- and concentration-dependent regression model. We applied the analysis strategy in a microarray case study exposing zebrafish embryos to 3 selected model compounds including 2 cyclooxygenase inhibitors. After identification of key responses in the transcriptome we could compare and characterize their association to developmental, toxicokinetic, and toxicodynamic processes using the parameter estimates for affected gene clusters. Furthermore, we discuss an association of toxicogenomic effects with measured internal concentrations.

**Conclusions:**

The design and analysis pipeline described here could serve as a blueprint for creating comparable toxicogenomic fingerprints of chemicals. It integrates, aggregates, and models time- and concentration-resolved toxicogenomic data.

## Key Points

- Comparability between toxicogenomic experiments can be improved with the help of:
  - the zebrafish toxicogenomic universe – a self-organizing map (SOM) of various toxicogenomic datasets providing a common reference frame for biological interpretation, and
  - a regression model allowing quantitative characterisation of biological responses and inference on a concentration and time scale.
- In a case study the dynamics of key responses (related to e.g. developmental delay, stress response and cyclooxygenase (COX) inhibition) could be identified and discriminated.

## Background

Chemical risk assessment and environmental monitoring are challenged to find ways of accounting for a large variety and quantity of chemicals [1], which are developed, used, and discharged by modern societies [2], and to which wildlife [3] and humans [4] are exposed during the course of their lifetimes. Hence, methodological innovation for an improved and more comprehensive characterization of human and environmental exposures to chemicals [5] and their related effects [6] is sought.

Offering comprehensive response detection, toxicogenomic methods are suggested for an improved assessment of chemical-related hazards [7] or environmental health [8]. Because chemicals induce characteristic transcriptome changes (toxicogenomic fingerprints) in tissues [9] and whole organisms [10], ’omics approaches provide novel possibilities for exposure and effect diagnosis for ill-characterized chemicals and environmental samples [11, 12] and may extend the prediction of toxicity on the basis of mechanistic information [13]. In this regard, comparison or connection of different experiments becomes crucial for the interpretation of toxicogenomic observations [14].

When comparing gene expression profiles, the sheer amount of signals in an ’omics dataset poses a quest for comparison and extraction of relevant patterns [15]. The application of self-organizing maps (SOMs), a machine learning method developed by Kohonen [16], has been shown to be valuable for the comparison of transcriptome profiles of different tissues [15] and cancer subtypes [17]. Here, we aimed at improving comparability of toxicogenomic fingerprints with the help of a SOM. This is not yet an established approach in toxicogenomics.

Furthermore, comparability between toxicogenomic datasets is typically limited owing to substantial differences in study designs, e.g. with respect to selected exposure time and concentration [18]. In their pioneering studies investigating toxicogenomic fingerprints Hamadeh et al. [9] and Yang et al. [10] showed that responses vary with exposure time and concentration. This implies that comparative interpretations of toxicogenomic fingerprints undergo a risk of deriving ambiguous conclusions when the concentration and time dependence of the reported responses is neglected. Additionally, this severely limits the scope for interpretation or prediction of effects for untested exposures in risk assessment or monitoring efforts. Therefore, comparability would require means for extrapolation. Studies that have analyzed time- or concentration-resolved toxicogenomic fingerprints applied correlation networks (e.g., [19]), unsupervised clustering (e.g., [20]), or a set of different regression models (e.g., [21,22]) to describe the responses. However, an integration of concentration and time dependence in one model has not yet been achieved for toxicogenomic responses. Therefore, in this study we strived for establishing a regression model capturing the time and concentration dependence of toxicogenomic responses.

Taken together we aimed to integrate, aggregate, and model dynamic toxicogenomic responses in order to obtain aggregated compound fingerprints, which can be extrapolated on the scale of exposure duration and concentration, and which are comparable between different compounds and studies.

To address the raised issues, we developed an analysis pipeline combining the algorithm of SOMs with a concentration- and time-dependent response model (CTR model). With the SOM we integrated previously published toxicogenomic data to a reference frame that we called "toxicogenomic universe" and aggregated toxicogenomic fingerprints from single compounds to this reference frame to foster comparison between the fingerprints. A regression model was built to derive quantitative parameters for comparing response dynamics and extending the scope for inference.

To demonstrate the added value of the suggested approach, we performed an experimental case study and applied the pipeline on microarray data of the zebrafish embryo (ZFE) (*Danio rerio*) after exposure to 3 selected environmentally relevant contaminants. The experimental design covered 6 different exposure durations and 5 increasing compound concentrations. The 3 compounds were diclofenac and naproxen, 2 pharmaceuticals known to inhibit the enzyme cyclooxygenase (COX) in humans, and diuron, a herbicide known to target the arylhydrocarbon receptor (AHR) pathway in mammalian cells [23].

Besides gene expression we also measured the internal concentrations of all 3 compounds after the exposure. Together with parameter estimates from the CTR model, this allowed us to separate toxicodynamic from toxicokinetic responses. Finally, we discuss the suggested analysis pipeline for achieved progress in inferential statements on compounds' effects, and outline further uses in the field of toxicogenomics.

## Data description

In this study we integrated transcriptome data of the ZFE from public databases with transcriptome data from our own exposure experiments to infer a universal SOM. Based on this, we performed a case study further investigating the time- and concentration-resolved toxicogenomic fingerprints from our exposure experiments. In this section we briefly describe the dataset used for generating the SOM and explain the experimental design and selection of model compounds for the case study.

### Dataset for generating the toxicogenomic universe

For the generation of a toxicogenomic universe for the ZFE we used the toxicogenomic fingerprints of the model substances measured in this study in combination with previously published toxicogenomic fingerprints in the ZFE. Data were selected, downloaded, and processed from Gene Expression Omnibus (GEO) and ArrayExpress in a semi-automatic workflow, which can be accessed via protocols.io [24]. A summary of datasets included in our study is provided in Table S1. The included microarray platforms were annotated to the most recent zebrafish genome (Genome Reference Consortium Zebrafish Build 11), and Ensembl gene annotation (Ensembl database release 93 [25]).

### Case study

For our case study, investigating time- and concentration-dependent toxicogenomic responses in the ZFE, we selected 3 environmentally relevant model compounds, namely, diuron, diclofenac, and naproxen:

Diclofenac (CAS RN:15307-79-6) is used as a pharmaceutical substance, often applied as a painkiller and to reduce inflammation. It belongs to the group of non-steroidal anti-inflammatory drugs (NSAIDs) and is a known inhibitor of both variants of the COX enyzme. COX produces prostaglandins, which act as inflammatory signalling molecules (reviewed in [26]). By inhibiting COX an inflammatory response is repressed. As environmental toxicant, diclofenac gained attention owing to its toxicity in vultures, which has led to a significant decline in the vulture population in Pakistan [27]. Furthermore, it was identified as a priority pollutant in aquatic environments (e.g., [28]). Several toxicological studies were performed using aquatic organisms (reviewed in [29]). In fish, adverse effects of diclofenac exposure on gill, liver, kidney, and the gastrointestinal tract, as well as reduced egg growth and delay in hatching, have been reported. Diclofenac has also been associated with drug-induced liver toxicity in response to the formation of reactive metabolites, mitochondrial dysfunction, and impairment of ATP synthesis [30, 31].

Naproxen (CAS RN:26159-34-2), like diclofenac, is widely applied as a COX inhibitor of the NSAID group. It has been detected in surface waters [32, 33, 28] and it was shown to lead to histopathological liver damage and pericardial edema in ZFEs [34].

The third compound used in this study was diuron (CAS RN:330-54-1), a herbicide listed as a priority substance to be monitored under the European Water Framework Directive [35]. In plants, it is known to specifically inhibit the electron transfer from photosystem II. In mammalian cells, it was found to bind to the AHR [23]. In the ZFE, diuron has been reported to provoke sublethal effects on heartbeat and spontaneous movements [36]. We thus expected diuron to act differently compared with diclofenac and naproxen.

#### Experimental design

Exposure settings for our transcriptome measurements were designed to meet several requirements: we intended to follow compound-specific toxicodynamic processes but also account for differences in toxicokinetics. Most importantly, results were meant to be comparable among the different compounds.

The exposure for a standard ZFE toxicity test starts immediately after fertilization [37]. However, because we expect many unspecific effects when disturbing the first hours of development, we opted for an exposure period between 24 and 96 hours post fertilization (hpf). Time points of RNA extraction during the exposure were 3, 6, 12, 24, 48, and 72 hours post exposure (hpe). The exposure concentrations were phenotypically anchored to the lethal concentration (LC) at 96 hpf/72 hpe. The LC_25_, modelled from experimental observations (see Supplementary Methods), served as highest and the LC_0.5_ as lowest exposure concentration with 6 equal dilution steps in between, with dilution steps 1, 2, 4, and 6 chosen for exposure (see Equations [1] and [2], Fig. S1). The selected concentrations for transcriptome experiments can be found in the Supplementary Methods file.

(1) \begin{equation*}
\textrm{Dilution factor (df)} = \sqrt [6 \ ] {\frac{\mathrm{LC}_{25}}{\mathrm{LC}_{0.5}}},
\end{equation*}

(2) \begin{equation*}
\textrm{Exposure concentrations} = \frac{\mathrm{LC}_{25}}{df^{x}}; x = {0,1,2,4,6}.
\end{equation*}

## Data analyses and Results

**Figure 1:**
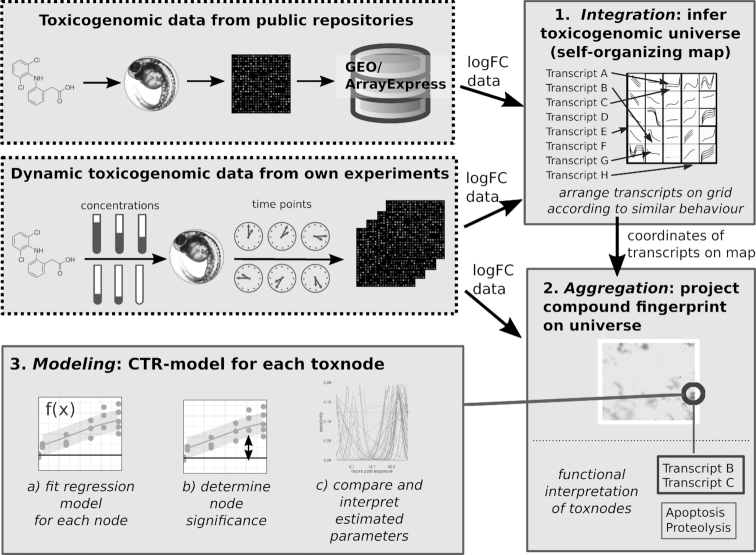
Flowchart of analysis pipeline to obtain dynamic toxicogenomic fingerprints.

Our analysis aimed at obtaining aggregated dynamic toxicogenomic fingerprints from the measured transcriptome data. The key parts of the analysis workflow (depicted in Fig. 1) are

**Figure 2:**
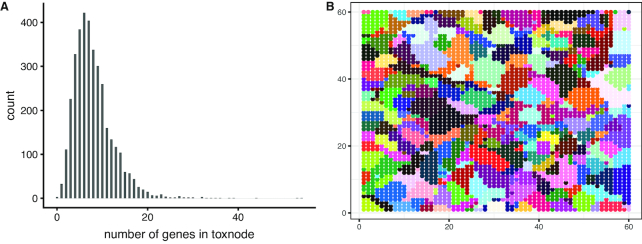
Response integration: The Zebrafish Embryo Toxicogenomic Universe (ZTU) comprising 3,600 toxnodes. A, Number of genes per toxnode. B, 118 clusters of toxnodes, each color representing a distinct cluster. For cluster assignments also compare Table S2.

**Figure 3:**
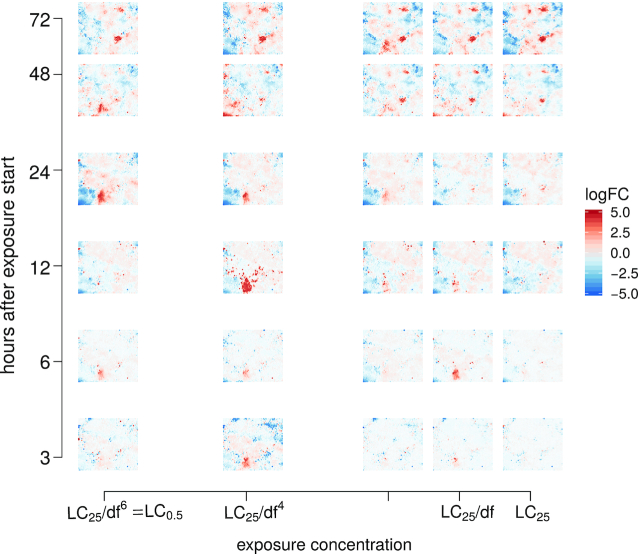
Response aggregation: Toxicodynamic fingerprint of naproxen projecting the responses of 30,000 transcripts on 3,600 nodes in the toxicogenomic universe. Shown is a grid of the mean logFC fingerprints for each sampled time point/exposure concentration. df: dilution factor (1.15 for naproxen); LC_25_: exposure concentration at which 25% of embryos show lethal effects after 72 hours of exposure (309 μmol/L for naproxen).

**Figure 4:**
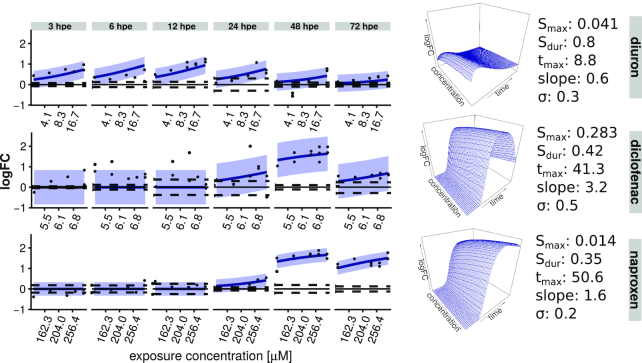
Response modelling: Model fit for response of toxnode No. 1119—containing the gene for nfe2l2b—towards model compound exposure. The regression model allows a 3D interpolation of time and concentration dependence. Shaded areas indicate a 95% confidence interval; dashed lines indicate 2.5%/97.5% quantile of the respective controls.

**Figure 5:**
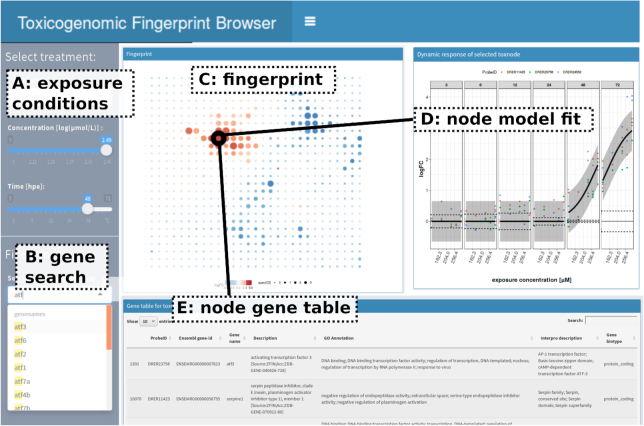
Response exploration: Screenshot of interactive toxicogenomic fingerprint browser (available via https://webapp.ufz.de/itox/tfpbrowser). This online tool provides access to visualizations of toxicogenomic fingerprints, model fits, and detailed investigation of single toxnodes.

- **Integration** of previously published and new toxicogenomic datasets using an SOM into the Zebrafish Embryo Toxicogenomic Universe (ZTU) (Fig. 2);
- **Aggregation** of compound toxicogenomic fingerprints by projection onto the ZTU (Fig. 3);
- **Modelling** of time- and concentration-resolved responses using a regression model (Fig. 4);
- **Exploration** of the analysis results with the help of an interactive toxicogenomic fingerprint browser (Fig. 5).

In the following, we describe the analysis steps and the respective results in more detail. Subsequently, we report the results of a case study in which we applied the workflow.

### Integration: the Zebrafish Embryo Toxicogenomic Universe

A compiled dataset of published toxicogenomic data was combined with data from our 3 single-compound exposures to infer the ZTU based on all currently retrievable toxicogenomic ZFE microarray data. All datasets were normalized against the respective control of the same experiment. The resulting dataset containing log_2_(fold change) (logFC) values from 342 different treatments and for 29,046 unique genes was used to infer a SOM (Fig.   2). This method organizes genes into groups of co-regulated or co-expressed transcripts. Those groups are arranged on a 2D grid in a way that similar behaving (i.e., co-expressed) groups end up in the same regions. Each coordinate on the map gets assigned a distinct group of genes. Because the ZTU is derived from toxicogenomic data we call this coordinate "toxnode" with reference to the term "node" used in general network terminology (equivalent to the term "metagene" in Wirth et al. [15]). The outcome of this step is a 60 × 60 grid of 3,600 toxnodes. Each gene present in our dataset is permanently assigned to a toxnode, while each node contains genes that behave similarly across all exposure conditions. The number of genes per toxnode ranges from 1 to 54, with a mean of 8 genes per node (Fig.   2A).

To obtain an overview of the ZTU we grouped the 3,600 toxnodes into 118 clusters (which we determined to be among the optimal cluster sizes; see Supplementary Methods) with the help of k-means clustering. To enable easy description of the clusters a random name was assigned to each cluster of nodes. The clustering is visualized in Fig. 2B and summarized in Table S2. The resulting clusters contained between 3 and 93 toxnodes, with a mean of 31.

The data integration and clustering with the help of the SOM and subsequent k-means clustering may help in biological interpretation of toxicogenomic responses in the ZFE. We performed an over-representation analysis for functional annotation terms from the databases ZFIN [38], InterPro [39], Reactome [40], and Gene Ontology (GO) [41, 42]. Biological annotations of at least 1 of the 4 databases are significantly enriched for 100 of 118 clusters in the ZTU (Tables S3−S5). The clusters with the highest proportion of genes assigned to a common function in the 4 databases are cluster "Trae", containing mainly a set of different crystallin genes (InterPro domain: β/γ crystallin in 37 of 52 genes, enriched with an adjusted *P*-value of $3\times 10^{-89}$; ZFIN: solid lens vesicle, 15 of 52 genes, adjusted *P*-value $5\times 10^{-11}$); the cluster "Dakota", containing different vitellogenin genes (GO: lipid transporter activity, 5 of 10 genes, adjusted *P*-value $7\times 10^{-11}$; ZFIN: unfertilized egg, 3 of 10 genes, adjusted *P*-value $6\times 10^{-6}$; interpro: vitellogenin,open β-sheet, 6 of 10 genes, adjusted *P*-value $9\times 10^{-19}$); and cluster "Vincent", containing genes enriched for the upstream regulator RUNX1 as well as for oxygen transport (Reactome: RUNX1 regulates transcription of genes involved in differentiation of keratinocytes, 9 of 42 genes, adjusted *P*-value $2\times 10^{-21}$; GO: oxygen transport, 5 of 42 genes, adjusted *P*-value $9\times 10^{-11}$).

Examples of further functional enrichments for toxnode clusters, which are affected and explained in detail later on in our case study, are cluster "John", containing a set of genes expressed in the pancreas (ZFIN: pancreas, 14 of 87 genes, adjusted *P*-value $1\times 10^{-10}$); cluster "Karan", containing genes associated with cell death (GO: regulation of cell death, 10 of 56 genes, adjusted *P*-value $7\times 10^{-5}$); cluster "Pauline", containing genes associated with phase II biotransformation (Reactome: *Danio rerio:*phase II—conjugation of compounds, 10 of 56 genes, adjusted *P*-value $3\times 10^{-12}$); and cluster "Taamira", containing genes associated with the arachidonic acid (AA) pathway (GO: arachidonic acid metabolic process, 3 of 23 genes, adjusted *P*-value $2\times 10^{-5}$).

### Aggregation: compound fingerprints projected on the ZTU

The ZTU retrieved in the previous step can be used as a universal coordinate system to project any exposure-specific fingerprint. In Fig. 3 this is exemplarily shown for the treatment with naproxen (see Figs S2 and S3 for diuron and diclofenac treatments, respectively). Here, the mean logFC of each toxnode for the different exposure settings is shown. This allows us to obtain an impression of the response to exposure to a compound for the defined conditions.

We observe that the fingerprints show regulation in both directions (up- and down-regulation). It also becomes obvious that fingerprints differ between exposure compound, duration, and concentration but also show some commonalities. Contrary to expectation, diclofenac and naproxen—both known to inhibit the same enzyme—show distinctly different patterns in their toxicogenomic fingerprints.

These observations can already give some insight about the toxicogenomic responses, yet they only allow for anecdotal interpretations. For a more generalizable exploration, a modelling approach was deemed helpful and followed in the next step.

### Modelling: regression models for time- and concentration-dependent toxicogenomic responses

The analysis up to this step allows the consideration of findings for each exposure setting in isolation. To arrive at a more general and transferable response characterization, which allows more than qualitative extrapolation and comparison between substances, we strived for a quantitative description of the measured transcriptional changes. Therefore, we implemented a regression model, capturing the toxicogenomic responses over concentration and time for different substances.

The CTR model describes concentration dependence in a monophasic and time dependence in a biphasic manner. Therefore, we call it "mobi-CTR model" here. It is based on the Hill equation, a 3-parameter non-linear model, originally describing the binding of oxygen to haemoglobin as dependent on oxygen saturation [43]. Due to its flexibility on the one hand and physiological meaningfulness on the other hand, it was later on used in many applications (reviewed in [44]) and also proposed for pharmacological dose response modelling [45]. One representation of the Hill equation is provided in Equation (3). It is defined by the parameters logFC_max_, slope, and *X*_50_. The parameter logFC_max_ is the maximum logarithmic fold change observed for the respective transcript or toxnode, the slope defines the steepness of the curve, and *X*_50_ defines the concentration, for which the response (i.e., logFC) reaches half-maximum.

The progression of the response over time can be captured by a time-dependent description of the parameter *X*_50_ in Equation (3). Empirically, we discovered that the dynamics of the reciprocal of *X*_50_ is in many cases accurately captured by the logarithmic Gaussian function (Equation 4). We call the reciprocal of *X*_50_ "sensitivity" because a large value indicates a sensitive response. When inserting Equation (4) into Equation (3), we obtain a complete regression model describing the time- and concentration-dependent logFC after compound exposure (Equation 5):

(3) \begin{equation*}
\mathrm{logFC}(c) = \displaystyle\frac{\mathrm{logFC}_{\mathrm{max}}}{1+exp\left[-\mathrm{slope}*\left(\log(c)-\log (X_{50})\right)\right]},
\end{equation*}

(4) \begin{eqnarray*}
\mathrm{sensitivity}(t) = \frac{1}{X_{50}(t)}= S_{\mathrm{max}}*exp\left[-0.5*\left(\displaystyle\frac{\log(t)-\log(t_{\mathrm{max}})}{S_{\mathrm{dur}}}\right)^{2}\right],\nonumber\\
\end{eqnarray*}

(5) \begin{eqnarray*}
\mathrm{logFC}(c,t) &=& \frac{\mathrm{logFC}_{\mathrm{max}}}{1+exp\left[-\mathrm{slope}*\left(\log(c)-\log\left(\frac{1}{S_{\mathrm{max}}*exp\left(-0.5*\left(\frac{\log(t)-\log(t_{\mathrm{max}})}{S_{\mathrm{dur}}}\right)^{2}\right)}\right)\right)\right]}\nonumber\\
&&+\,\epsilon,\quad \epsilon \sim \mathcal {N}(0,\, \sigma ^{2}), 
\end{eqnarray*}

where logFC_max_ corresponds to the maximum fold change of the respective node across all conditions, *S*_max_ is the maximum sensitivity (1/EC_50_) of the gene, *t*_max_ is the point in time with maximum sensitivity, and *S*_dur_ represents a measure of duration of the sensitivity interval.

An exemplary model fit is shown in Fig. 4 for toxnode No. 1119. This node is sensitive in response to the exposure to all 3 substances. The different dynamics are reflected in the parameter estimates. For example, *t_max_* is substantially smaller for diuron (8.8 hpe) than for diclofenac (41.3 hpe) and naproxen (50.6 hpe), reflecting an earlier response for the former. The smaller values of *S*_dur_ for diclofenac (0.42) and naproxen (0.35) in comparison to diuron (0.8) indicate a shorter time frame of sensitivity for this toxnode regarding both of the COX inhibitors. The values of *S*_max_ indicate that the toxnode responds much more sensitively to diclofenac exposure in comparison with the other 2 compounds.

The mobi-CTR model was fitted to the measured responses (i.e, logFC) of each toxnode, arriving at a quantitative aggregation of time- and concentration-dependent toxicogenomic responses. In contrast to Figs 3, S2, and S3, we can now aggregate the response information to a single fingerprint, and accordingly a single plot, per substance, by projecting the estimates for a parameter on the ZTU. These aggregated fingerprints then allow a systematic analysis as we demonstrate in the case study below.

#### Quality of data description by fitted model

The model-fitting algorithm converged for all nodes and thus provided viable parameter estimates. There is no trivial measure for goodness of fit for non-linear models (such as *R*^2^ for linear models; cf. [46]). Therefore, the quality of data description by the model was determined using the small-sample Akaike information criterion (AICC)-weight compared to a null model. In the vast majority of cases the regression models are preferred over the null model (Fig. S4A−C). When comparing the regression models to the more flexible spline fit (Fig. S4D−F), which is assumed to offer the optimal data description here, there are (as could be expected) many toxnodes for which the spline provides better data description. However, for ∼20% of the nodes the mobi-CTR models are even preferred over a spline fit, thus indicating a good description of the data by the model fit. In contrast to the CTR model the spline fit does not offer much scope for inference. The major advantage of the CTR model is that the parameters can be interpreted in a biological context.

#### Selection of significantly affected toxnodes

Typically, we only expect a small fraction of the toxnodes to show a statistically significant response after exposure to a specific compound. To judge whether a node shows a significant regulation in our exposure scenario, we compared the 95% confidence interval for the regression model fits with the 2.5% and 97.5% quantiles of control measurements. We selected those nodes with a sum of differences between these curves above or below zero (see Fig. S5A for visualization). This resulted in a total number of 432 significantly affected nodes, with 60 nodes for diuron, 73 nodes for diclofenac, and 353 nodes for naproxen exposure meeting this criterion (Tables S7−S9). Eight nodes are regulated in both diuron and diclofenac exposures (1 in different directions), 22 nodes in diuron and naproxen (6 in different directions), and 18 nodes in diclofenac and naproxen exposures (3 in different directions); 3 nodes are regulated in exposures to all 3 compounds (Fig. S5B).

### Exploration: toxicogenomic fingerprint browser

To ease the exploration of the toxicogenomic fingerprints in the context of the ZTU, we created an online fingerprint browser [47]. A screenshot of the browser is shown in Fig. 5. The browser allows visualization of fingerprints of different exposure conditions and provides details about toxnode responses and genes that are assigned to the respective nodes. It is possible to select different substances and exposure conditions (Fig. 5A) or to search for genes in the universe (Fig. 5B). After treatment selection the respective fingerprint is shown (Fig. 5C). When selecting a toxnode on the fingerprint or searching for a specific gene name, the CTR model fit is shown (Fig. 5D). Furthermore, the member genes and some functional annotation are displayed (Fig. 5E).

### Case study: investigation of toxicogenomic fingerprints of 3 model compounds

**Figure 6:**
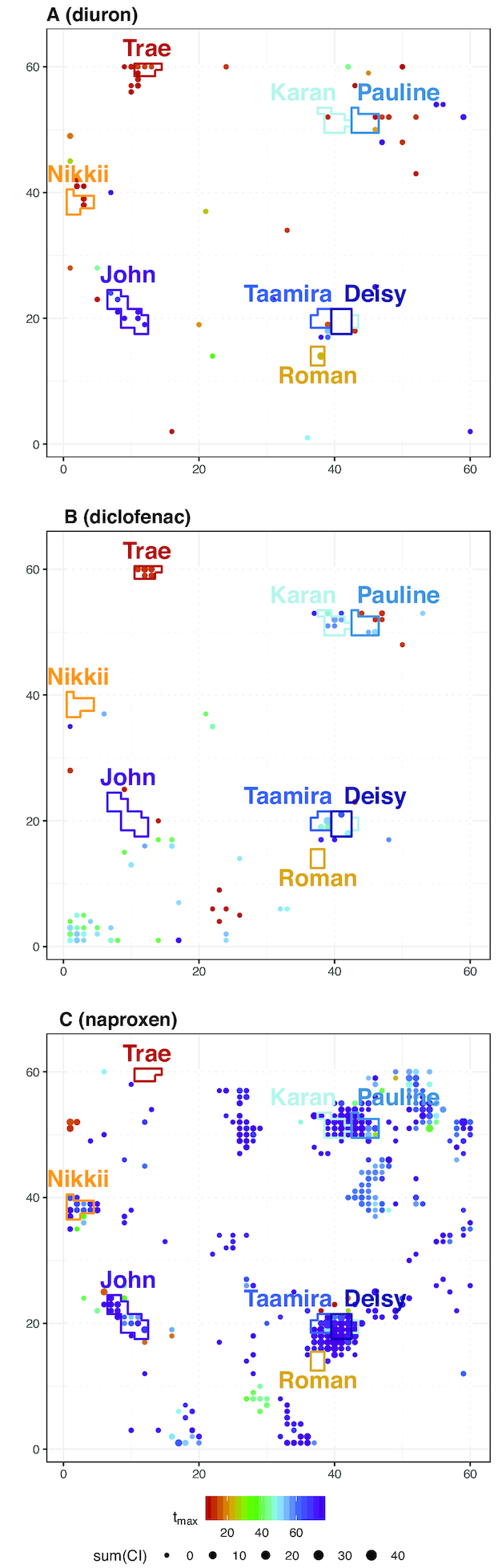
Case study: fitted parameter values for time point of maximum sensitivity (*t*_max_) of all significant toxnodes projected on toxicogenomic universe. Dot size represents significant effect level (sumCI; cf. Fig. S5A). Selected clusters are highlighted in the plots and are discussed in the text.

In order to demonstrate the added value of our approach, we conducted a case study and applied the described pipeline to toxicogenomic data of the 3 model compounds diuron, diclofenac, and naproxen. By applying a combination of fingerprint projection on the ZTU and regression modelling, as described above, we received quantitative, dynamic toxicogenomic fingerprints of the 3 compounds. This is exemplarily shown in Fig. 6, where generalized representations of the 3 dynamic fingerprints are visualized by a projection of parameter estimates for *t*_max_ on the ZTU. In the figure each significantly affected toxnode is coloured according to the estimated*t*_max_. The size of each dot indicates the extent of regulation for the measured conditions. Furthermore, some node clusters are highlighted, which we discuss below.

The figure shows that naproxen exposure affects considerably more toxnodes in the ZTU than exposures to diuron or diclofenac. We can identify commonly and differentially affected toxnodes and clusters on the map, e.g., cluster "John", comprising many toxnodes affected late during exposure to diuron and naproxen, cluster "Trae" affected early by diuron and diclofenac, or cluster "Roman" only induced by diuron, as we discuss in more detail below.

Generally, there are 2 kinds of information we can deduce from the dynamic toxicogenomic fingerprints: first, the clustering of genes into the same toxnode or region of the ZTU may indicate a common upstream regulator or common cellular process that the genes are involved in. Thus, the member genes of toxnodes affected by a compound exposure may provide qualitative functional information about the response. Second, the estimated model parameters provide quantitative information about the dynamics (i.e., *t*_max_, *S*_dur_) and the concentration dependence (i.e., *S*_max_, slope) of the effects. Additionally, the ratio of min(EC_50_)_morphological_/min(EC_50_)_toxnode_ (ratio_*m*__/*t*_) indicates the concentration range that lies between effects on toxnode regulation and morphological effects observable under the microscope.

In this regard, we inspected the toxicogenomic fingerprints retrieved by the pipeline (lists of significantly affected toxnodes can be found in supplementary Tables S7−S9). We explored the affected nodes and their model parameters in the fingerprint of diuron and compared this with the fingerprints of diclofenac and naproxen. In doing so, we specifically focussed on effects that could be linked to an exposure to COX inhibitors.

#### Diuron

We found 60 toxnodes in 35 clusters to be significantly affected in the fingerprint of diuron (Fig. 6A, Table S7). Here we focus on the effects in clusters "Roman", "Nikkii", "Trae", and "John".

##### Roman

The most prominently affected node in the fingerprint of diuron is No. 818 in cluster "Roman". It comprises genes for phase I biotransformation enzymes of the cytochrome P450 family Cyp1 (Cyp1a, Cyp1c1,Cyp1c2). The *t*_max_ was fitted to 18.4 hpe. With an *S*_dur_ of 0.8 it belongs to the toxnodes with the most sustained response after diuron exposure. Diuron is known to bind to the AHR in mammalian cells [23], which is an upstream regulator of CYP1 genes. The strong induction of these genes in the toxicogenomic fingerprint indicates a persistent interaction of diuron with the AHR in ZFE.

##### Nikkii

Additionally, we observed toxnodes 2223 and 2283 to be up-regulated in the fingerprint. These nodes are assigned to cluster "Nikkii", which is enriched for genes involved in the phototransduction pathway (Table S5). Both toxnodes were induced early (*t*_max_ 3.3 and 3.8 hpe, respectively) with a high sensitivity compared to other nodes (ratio_*m*__/*t*_ > 10). Similar parameter estimates were found for the 3 significantly induced nodes of cluster "Robert" (Nos. 3431, 3550, 3549) and "Tiana" (Nos. 3310, 3370, 3371), which are enriched for the retinal photoreceptor layer and the neuronal system (*t*_max_ between 1.5 and 3.8 hpe, ratio_*m*__/*t*_ > 10). This early induction of the phototransduction pathway was not observed with the other 2 compounds and may be connected to an observed increase of locomotor response after diuron exposure [36, 48]. Some of these nodes as well as some other nodes of the clusters "Nikki" and "Robert" were found to be down-regulated with naproxen, though significantly later and less sensitively, with a *t*_max_ between 57 and 75 hpe and a ratio_*m*__/*t*_ < 1.5 (see also Fig. 6).

##### Trae

Toxnodes 3551, 3552, and 3553 belong to cluster "Trae" and were down-regulated early after diuron exposure (*t*_max_ = 8 hpe for all 3 nodes). The same was observed with diclofenac, where 6 nodes of cluster "Trae" were down-regulated with similar values for *t*_max_ between 6.1 and 7.3 hpe (Fig. 6). This cluster did not seem to be affected in the naproxen fingerprint. As already mentioned above, cluster "Trae" is highly enriched for crystallin genes (Table S4).

##### John

In contrast to this early regulation, we found toxnodes 1151, 1328, 1149, 1211, 1092, and 1387, all belonging to cluster "John", to be down-regulated with diuron exposure, with a *t*_max_ between 62 and 74 hpe, indicating a late response. This cluster is significantly enriched for pancreatic enzymes (Table S6). We also observed these, as well as 6 additional nodes of the same cluster, to be down-regulated in the fingerprint of naproxen, with similar values for *t*_max_ estimated between 53 and 75 hpe (Fig. 6).

#### Diclofenac and Naproxen

By comparing the fingerprints of the 3 compounds we found 15 toxnodes to be significantly up- or down-regulated in the same direction in response to the 2 known COX inhibitors, naproxen and diclofenac, without showing a significant regulation in response to diuron. A selection of estimated CTR model parameters for these nodes are summarized in Table 1. Similarly as for diuron, the most prominently affected nodes after diclofenac and naproxen exposure contain genes for biotransformation enzymes (cf. Tables S8 and S9). Yet, the specific enzymes were in part different from the ones up-regulated with diuron exposure and were mainly located in the clusters "Taamira" and "Pauline" in the ZTU as opposed to cluster "Roman" under diuron exposure.

**Table 1.** Parameter estimates (mobi-CTR model) for toxnodes specifically affected by diclofenac and naproxen in our experiment

Toxnode No.	Genes	Cluster	logFC_max_	*t*_max_		*S*_max_		sum(CI)		ratio_*l*__/*t*_		ratio_*m*__/*t*_	
				Diclofenac	Naproxen	Diclofenac	Naproxen	Diclofenac	Naproxen	Diclofenac	Naproxen	Diclofenac	Naproxen
1118	*abcc2, CYP2C9, abcb5*	"Taamira"	2.2	44.1	75.0	0.283	0.017	2.84	7.80	2.3	6.1	2.0	3.0
1179	*cyp2k18*	"Taamira"	4.8	48.9	69.0	0.283	0.017	12.02	34.12	2.3	6.1	2.0	3.0
2985	*cbr1l, dhrs13l1, ethe1, mgst3b, sult6b1*	"Pauline"	2.1	52.6	53.7	0.094	0.017	0.01	2.31	0.8	6.1	0.7	3.0
1062	*lepa, pth1a*	"Deisy"	3.6	46.1	72.0	0.229	0.017	5.38	8.60	1.9	6.1	1.6	3.0
1241	*hsp70l, hsp70.1, hsp70.2*	"Deisy"	3.3	61.6	64.0	0.149	0.017	2.35	10.39	1.2	6.1	1.1	3.0
3039	*dusp1, cyr61, gadd45ba, sik1, nfkbiaa*	"Karan"	1.7	55.2	72.9	0.146	0.005	0.07	0.11	1.2	1.8	1.0	0.9
3040	*btg2, zgc:162730, tcima, ier2b, egr2a, jdp2b*	"Karan"	2.1	55.6	75.0	0.148	0.007	0.09	1.33	1.2	2.5	1.1	1.2
3100	*socs3a, fosab*	"Karan"	3.3	53.8	73.0	0.180	0.006	2.59	2.96	1.5	2.1	1.3	1.1
3101	*serpine1, atf3, junba*	"Karan"	3.2	54.0	75.0	0.137	0.007	0.53	7.96	1.1	2.5	1.0	1.2
1000	*isg15*	"Farajallah"	5.1	72.4	75.0	0.113	0.008	0.08	17.17	0.9	2.9	0.8	1.4
3161	*socs3b, timp2b, CR855311.1, clu*	"Farajallah"	2.6	63.9	74.7	0.141	0.007	0.06	4.89	1.1	2.5	1.0	1.2
17	*RF00020*	"Tashina"	2.5	75.0	62.6	0.140	0.007	0.72	5.47	1.1	2.5	1.0	1.2
2041	*si:ch211-125e6.5, si:ch211-125e6.14, zgc:172053*	"Vincent"	1.5	75.0	74.3	0.164	0.004	0.02	0.61	1.3	1.4	1.2	0.7
3157	*igfbp1b*	"Daniel"	2.7	73.6	75.0	0.136	0.009	0.08	5.83	1.1	3.2	1.0	1.6
3173	*pdzd3a*	"Talon"	$-$2.0	49.3	74.0	0.131	0.007	$-$0.05	$-$4.33	1.1	2.5	0.9	1.2

ratio_*l*__/*t*_: min(LC_50_)/min(EC_50_)_toxnode_; ratio_*m*__/*t*_: min(EC_50_)_morphological_/min(EC_50_)_toxnode_. Positive sum(CI) indicates up-regulation, while negative indicates down-regulation of respective toxnodes. logFC_max_ represents maximum logFC per node across all treatments in our experiments.

##### Taamira

Cluster "Taamira" is, among others, enriched for genes annotated with phase I functionalization and AA metabolism (Table S5). It contains 2 toxnodes, which are specifically induced by the COX inhibitors: node No. 1179 is most prominently affected with both diclofenac and naproxen exposures and contains the gene *cyp2k18*, coding for a phase I metabolic enzyme of the cytochrome P450 family, which was shown to be induced by different known hepatotoxicants in [49]; the neighbouring node No. 1118 contains a gene coding for Cyp2c9 (a paralogous enzyme of Cyp2k18) and the genes *abcc2* and *abcb5* coding for ABC transporter proteins. The membrane transporter Abcc2 is known to eliminate especially phase II biotransformation products including conjugated drugs from the cells [50]. The affected nodes in cluster "Taamira" have a fitted *t*_max_ between 44 and 54 hpe for diclofenac, which is ∼25 hours later compared to the regulation of biotransformation induced by diuron. For naproxen the fitted *t*_max_ of these nodes is falling between 69 and 75 hpe, and therefore another 15 hours later compared with diclofenac (cf. Table 1). The sensitivity of the nodes is comparably high for both compounds but higher for naproxen, with a ratio_*l*__/*t*_ of 6.1 compared with 2.3 for diclofenac.

##### Pauline

In cluster "Pauline", which is enriched, among others, for phase II biotransformation, glutathione transferase activity, and detoxification of reactive oxygen species (Tables S3 and S5), toxnode 2985 is specifically up-regulated by diclofenac and naproxen. It contains genes coding for metabolic enzymes such as *carbonyl reductase 1−like enzyme* (*cbr1l*), *dehydrogenase/reductase SDR family member 13−like 1* (*dhrs13l1*), *persulfide dioxygenase* (*ethe1*), *microsomal glutathione S-transferase* (*mgst3b*), and a sulfotransferase (*sult6b1*). With a *t*_max_ of 52.6 hpe (diclofenac) and 53.7 hpe (naproxen) it belongs to the earliest regulated toxnodes appearing in the fingerprints of both diclofenac and naproxen. Other toxnodes of this cluster, such as No. 3045, are as well induced with both COX inhibitors, even though not significantly with diclofenac, and contain genes such as *peroxiredoxin* 1 (*prdx1*) or glutathione S-transferases (*gsta2, gstp1*) and reductases (*gsr*). Most of these enzymes belong to the group of oxidoreductases, and the results of the over-representation analyses indicate their involvement in response to oxidative stress.

##### Deisy

Two of the most prominently induced toxnodes in diclofenac and naproxen fingerprints belong to cluster "Deisy": toxnode No. 1062 is up-regulated with a *t*_max_ of 46 hpe with diclofenac and 72 hpe with naproxen. It contains the genes for the 2 hormones leptin α (*lepa*) and parathyroid hormone 1a (*pth1a*); toxnode 1241 contains different variants of the heat-shock protein Hsp70 and is induced with *t*_max_ values of 62 and 64 hpe for diclofenac and naproxen, respectively. The induction of *HSP70* by NSAIDs has been shown before (e.g., [51]). Also, a change in leptin levels after diclofenac exposure has been reported [30]. The induction of *lepa* might be linked to the AA pathway [52], which is disturbed by the inhibition of COX [53]. Additionally, leptin levels are related to the state of energy metabolism [54], which indicates that the COX inhibitors might induce a change in energy metabolism in the ZFE. This is further corroborated by the induction of toxnode 1120 in the same cluster (not significant with diclofenac), containing the genes for *cocaine- and amphetamine-regulated transcript 3* (*cart3*) and *apoptosis facilitator Bcl-2-like protein 14* (*CABZ01020840.1*). Up-regulation of *cart3* has been associated with anorexigenic effects in response to stress in adult zebrafish [55], while the BCL2 family of proteins is known to regulate stress-induced apoptosis [56].

##### Karan/Farajallah

Further COX inhibitor−specific toxnodes (Table 1) belong, among others, to cluster "Karan" (Nos. 3100, 3101, 3039, 3040) and the cluster "Farajallah" (Nos. 3161, 1000). Cluster "Karan" is significantly enriched for MAP-kinase phosphatase activity (Tables S3−S5; e.g., gene *dusp1*), transcription factors of the AP1 family, and the toll-like receptor cascade (Tables S4 and S5; e.g., genes *fosab, jdp2d, atf3, junab, nfkbiaa*), as well as the regulation of cell death and cell cycle (Table S3; e.g., genes *cyr61, gadd45ba, junba*). Additionally, it is enriched for an inflammatory response to biotic stimulus, which is also true for cluster "Farajallah", which is, in line with that, also enriched for the complement cascade (Tables S3 and S5). Furthermore, toxnodes containing genes known to be involved in immune response are part of cluster "Karan" but only found to be affected by naproxen. For example, this is toxnode 3041, containing COX2b (here *ptgs2b*), *serum/glucocorticoid regulated kinase 1* (*sgk1*), *CCAAT enhancer binding protein β* (*cebpb*), which all have been shown to be involved in inflammation [57, 58].

##### Regulation of Karan/Farajallah by Deisy

Several genes and pathways observed to be up-regulated in cluster "Karan" have been reported to be regulated by leptin, which is induced comparatively early within the cluster "Deisy" (see above). For example, Leptin is known to induce mitogen-activated protein kinase (MAPK) cascades as well as the JAK/STAT signaling pathway (reviewed in [59]) and stimulate the expression of SOCS3 as feedback regulator as well as TIMP1 (reviewied in [60]). The stimulation of c-FOS genes by leptin mediated via the STAT3 pathway was also reported before [61]. Furthermore, leptin and parathyroid hormone (PTH) were shown to regulate COX-2 messenger RNA expression [62–64]. Therefore, we hypothesize that leptin is one of the key regulators of the responses in cluster "Karan", which are induced later than Deisy, with a *t*_max_ of 54−55 hpe with diclofenac exposure and 73−75 hpe with naproxen exposure.

The *t*_max_ values of the cluster "Farajallah" (64−72 hpe with diclofenac exposure, 75 hpe with naproxen exposure) indicate an even more downstream response induced after the induction of "Karan". With a ratio_*l*__/*t*_ between 0.9 and 1.5 (diclofenac) and 1.8 and 2.9 (naproxen) the sensitivity of responses in "Karan" and "Farajallah" is lower in comparison to the nodes in "Taamira" or "Deisy".

#### Common responses in all 3 compound fingerprints

With our analysis pipeline we identified 3 toxnodes as significantly induced with all 3 compounds, namely, node No. 2986 (cluster "Pauline"), containing 1 gene coding for the phase II enzyme Ugt1a; its potential regulator Nfe2l2b in node 1119 (cluster "Taamira"); and node 998 (cluster "Farajallah") containing an orthologue gene for Cathepsin S. The early induction of *nfe2l2b* (alias *NRF2B*), a master regulator of oxidative stress [65, 66], and the induction of its potential target gene *ugt1a* [67], hint to the induction of the oxidative stress response cascade in the ZFE. Furthermore, the dynamics of this induction (e.g., see Fig. 4) is different between the compounds and seems to follow the chemical uptake dynamics of the compounds, which we discuss in more detail below. Further nodes of these 3 clusters were induced with all 3 compounds.

#### Global sensitivity dynamics

We observed above that the sensitivity dynamics of selected toxnodes substantially differs between the investigated compounds. We analyzed whether there are global differences in sensitivity dynamics between the compounds by examining the distributions of parameter estimates for *t*_max_ and *S*_max_.

**Figure 7:**
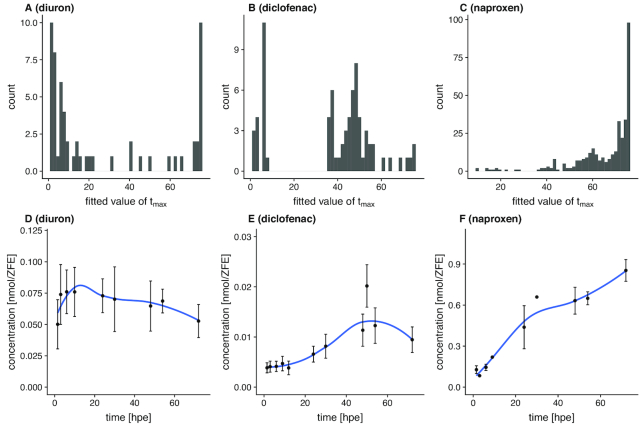
Sensitivity dynamics and toxicokinetics. A−C, Distribution of fitted parameter values among significantly regulated nodes for *t*_max_; D−F, measured internal concentration (mean ± standard error).

Diuron exhibits the most distinct early regulation of the 3 investigated compounds. This is also reflected in the distribution of estimates for *t*_max_ (Fig. 7A), showing 2 peaks at ∼1.5 and 75 hpe for all significantly affected toxnodes. The *S*_max_ of some affected nodes is calculated to be up to 2 orders of magnitude higher than the sensitivity for lethality (no morphological sublethal effects were observed for diuron exposure). The median ratio between morphological and toxicogenomic sensitivity of all significantly affected toxnodes is 2.8.

The response to diclofenac exposure shows 2 peaks at 7 and 50 hpe (Fig. 7B), which is 4 and 47 hours later, respectively, than the first peak of responses with diuron exposure. There are only a few toxnodes with a *t*_max_ later than 60 hpe. For diclofenac the ratio between morphological sensitivity and toxnode *S*_max_ is not >2.3 for any of the affected toxnodes. The median ratio is 1.4.

Naproxen clearly shows the latest response of the 3 substances, reflected by the distribution of *t*_max_ showing a small peak at ∼60 hpe and a high peak at the latest time point at 75 hpe. The ratio_m__/*t*_ shows a maximum of 6 for some of the toxnodes. The median ratio is 3.2.

#### Internal concentrations

The observed dynamics of transcriptional responses seems to be partially linked with the temporal pattern of internal chemical concentrations. Fig. 7 D−F depicts the increase of internal chemical concentration for the 3 compounds. It demonstrates different kinetics of chemical uptake in the ZFE. Whereas the highest internal dose of diuron is reached before 20 hpe (Fig. 7D), this peak is observed between 40 and 60 hpe with diclofenac (Fig. 7E) and not before the last observed time point at 72 hpe with naproxen (Fig. 7F). This matches with the observation that most affected toxnodes show a *t*_max_ < 20 hpe for diuron (Fig. 7A), between 40 and 60 hpe for diclofenac (Fig. 7B), and not before the last time point at 75 hpe for naproxen (Fig. 7C).

By comparing the CTR model fit depicted in Fig. 4 for the oxidative stress response marker *nfe2l2b* with the internal concentrations dynamics, we see a correlation of these results with *t*_max_ values of 8 hpe for diuron, 41 hpe for diclofenac, and 51 hpe for naproxen (cf. Figs 4 and 7D−F).

Overall, this shows that toxicogenomic sensitivity can be strongly influenced by toxicokinetic properties of the respective substances. The comparison of parameter values of the CTR model such as the *t*_max_ with additional information such as the internal dose dynamics also led to the identification of stage-specific, toxicokinetic-independent responses such as the down-regulation of the clusters "Trae" and "John".

## Discussion

The objective of this study was to improve comparability of toxicogenomic datasets by advancing the scope of inference for toxicogenomic fingerprints. Therefore, we developed and tested an experimental and data analysis pipeline for creating dynamic toxicogenomic fingerprints of chemicals. Here, we discuss the suggested approach as to the aspired comparability and scope for inference, as well as the added value with regard to the elucidation of molecular, cellular, or physiological effects of chemicals.

### Approach: map and model toxicogenomic responses

Our pipeline tackles 2 major challenges with regard to toxicogenomic analyses: first, to integrate and aggregate toxicogenomic datasets; and second, to integrate time and concentration dependence.

#### Integration and aggregation of toxicogenomic responses

A wealth of gene expression signatures is publicly available (e.g., GEO containing ∼2.7 million samples in October 2018), and efforts are increasing for gaining new insights by integrating large numbers of datasets. For example, the connectivity map approach, establishing links between similar gene expression profiles [14], was applied by Wang et al. [68] to explore similarities between toxicogenomic fingerprints in fish. This method can serve as a relatively simple and easily scalable approach to find similar profiles in a toxicogenomic database. However, the pairwise linking is based on qualitative lists of differentially expressed genes only, no explicit inclusion of time and concentration resolution is considered, and the outcome does not immediately aid in aggregating the responses of a single experiment; i.e., aggregation takes place on the level of metadata only.

Another approach for integrating toxicogenomic datasets is the inference of gene networks based on correlation or mutual information. These networks can be analyzed for modularity and interrogated for specific changes in nodes or edges after chemical perturbation. This was applied by Perkins et al. [13], for example, to reverse engineer adverse outcome pathways (AOPs) from mutual information networks, or by Woo et al. [69] to identify drug targets by analysing altered network interactions. However, comparability and integration of dependent variables such as time or concentration are still limited with these approaches.

A specific, more rigid network form is the SOM. The algorithm was developed by Kohonen [16]. It was first applied to gene expression data by Törönen et al. [70] and Tamayo et al. [71] and has been further developed and tested for aggregating tissue expression profiles [15, 72]. While SOMs have mainly been used to aggregate information from single datasets, we applied SOMs to integrate an extensive compilation of toxicogenomic datasets, and use the resulting grid afterwards to aggregate the fingerprints of single substances. A SOM shows limitations in capturing complex interactions compared to the more "flexible" networks in the aforementioned studies (i.e., connections between nodes are only formed between direct neighbours on the map). Therefore, some relevant interactions between transcriptomic units might get lost with the SOM. Additionally, co-expression of genes is not necessarily consistent across different perturbations. For example, when a compound binds to a transcription factor and thereby modulates its activity, co-expression with its target genes will significantly decrease [69]. This would not be captured in the dynamic toxicogenomic fingerprints as shown here. However, SOMs have the advantage of enabling visualization, interpretation, and comparing treatments on a whole-transcriptome scale, as well as reducing the number of analyzed entities. In our approach we could reduce the analysis space from ∼30,000 transcripts to 3,600 toxnodes or 118 clusters. Altogether, this allowed us to comprehensively analyse commonly and differentially regulated toxnodes and clusters in the ZTU and to derive functional hypotheses from this (see below). A common functionality of genes within some of the identified clusters was confirmed in our over-representation analysis (e.g., the clustering of crystallin or pancreas genes). Such a "recovery of the known" demonstrates the viability of the approach [14].

With the help of the SOM we can retrieve information about co-expression of genes from publicly available toxicogenomic datasets and include this information into our analysis. This way we can make use of these heterogeneous data and gain information for clustering the genes irrespective of differing experimental designs and occurrence of missing data. While the projection of toxicogenomic fingerprints on the ZTU increases accessibility and comparability of past findings (cf. Fig. S8) one should be aware of the limitations of such comparisons due to differences in experimental factors as discussed by Schüttler et al. [18]. Here, we show how the application of regression models could foster comparisons between studies with different exposure concentrations and time frames (see below).

In this context, the clustering of genes into toxnodes allows the combination of data aggregation with modelling of time and concentration dependence. The aggregation also improves model quality because data from several genes can be used for estimating a single parameter set (see below). The projection of toxicogenomic responses on a universal map fosters comparison between different profiles, which can be compared visually and quantitatively with the help of model parameters, as discussed below. While in our study the analysis focused on microarray data, the approach is not limited to microarray data and could also be applied to RNA sequencing data. This is also demonstrated in Fig. S8B, showing RNA sequencing data projected on the toxicogenomic universe. The supplementary methods file (supplementary_methods.html) demonstrates how to use our supplied R package toxprofileR and the toxicogenomic universe to infer toxicogenomic fingerprints of additional compounds.

#### Modelling of time and concentration dependence

A couple of studies have been published that investigated toxicogenomic fingerprints at varying exposure settings. Among those, only a few studies investigated the dynamics of responses. One example is the study by Alexeyenko et al. [19], who studied effect propagation at several time points after dioxin exposure in ZFEs and found changes in gene-gene interactions between different points in time. The application of several concentrations in toxicogenomic experiments is reported more frequently (e.g., [62, 73, 74]). Yet, all of the mentioned studies analyzed the different exposure conditions in isolation, thus only allowing qualitative statements about time- or concentration-dependent changes. In a study by Hermsen et al. [20] genes were clustered according to their concentration dependence across 7 different concentrations. Although this approach acknowledged concentration as a continuous variable, the description of concentration dependence remains observational in this study, also.

This was advanced in studies by Thomas et al. [21] and Smetanová et al. [22] that showed that concentration dependence of toxicogenomic responses can be captured by using regression modelling on significant responses applying a selection of different models. The individual description of responses using different regression models, however, limited the comparability.

In contrast to these studies ([21,22]), we fitted a uniform CTR model to all toxnodes in the toxicogenomic universe in order to derive node-specific parameter estimates. Subsequently, we used the confidence intervals of the fitted model for detection of statistical significance. This approach might have the limitation that we cannot capture each response as accurately as the aforementioned studies. For example, toxnodes showing a biphasic response on the concentration scale would not be accurately captured. Biphasic responses of gene expression have been reported to commonly occur in response to chemical exposure [22]. Especially the activation of steroid hormone receptors favors a biphasic response in gene expression [75]. This might limit the applicability of our model for endocrine-acting substances. Yet, our approach implicates advancements regarding comparability in several ways: first, there is a unique set of model parameters, whose estimates can be compared across different transcripts, toxnodes, or compound exposures. Second, model parameters are fitted independently from the statistical significance of changes between treatment and control; i.e., no preselection of genes is necessary. Therefore, model parameters can be compared between significantly and non-significantly regulated toxnodes. Moreover, significance of regulation is rather determined by a mechanistically motivated model (i.e., an adaptation of the Hill model), adding biological significance to a mere statistical treatment vs control comparison. Additionally, the aggregation of the responses of several transcripts into toxnodes enhanced the robustness of the model fits and implies that varying responses of single transcripts have a reduced impact on the aggregated outcome of a toxnode in the toxicogenomic fingerprint.

Furthermore, the time dependence of responses is not considered in previous approaches [21, 22]. The mobi-CTR model adapted in our study captures both concentration and time dependence of the responses. In this way, the model allows inference of a 3D, time- and concentration-resolved response pattern as shown in Fig. 4.

The application of the mobi-CTR model in the analysis of toxicogenomic responses has implications for the experimental design. Because response information is not based on pairwise comparisons (treatment vs control) but on time- and concentration-resolved response characteristics, we can use a dense sampling design with a few replicates only. This is in accordance with a study by Sefer et al. [76], which showed that in high-throughput testing dense sampling should be preferred over replicate sampling. Our findings reveal that the measurement of only 1 time point or a single concentration would not have been sufficient to identify toxnodes as being commonly regulated by all 3 substances as illustrated in Fig. 4.

**Figure 8:**
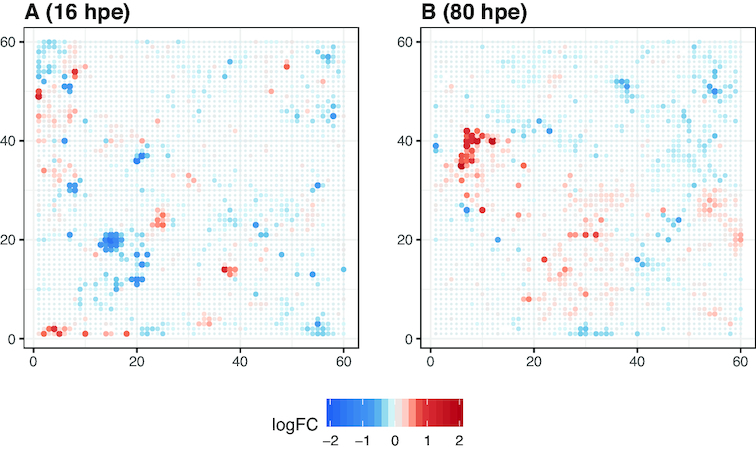
Predicted toxicogenomic fingerprints for diuron exposure at the environmentally relevant concentration 0.86 μmol/L. A, 16 hpe; B, 80 hpe. Both concentration and time point are outside the measured range in our case study.

Because it is not conceivable that the concentrations selected and measured in experiments will ever cover the whole range of concentrations relevant for estimating or interpreting environmental effects, measures for extrapolation become relevant. In this regard, the CTR model allows hypotheses and predictions to be built about toxicogenomic effects at conditions not measured. For example, with the help of the model we can predict for each toxnode in the ZTU which response we would expect for an environmentally relevant concentration of 0.86 μmol/L of diuron (concentration measured in [77]). This falls outside the concentration range measured in our study. In Fig. 8 the expected logFC values for 16 and 80 hpe are projected on the ZTU. Without investigating the profiles in detail, we can observe that the predictions show distinctly different profiles. A prominently down-regulated cluster can be seen in the early time point (blue spot bottom left, Fig.   8A) and a prominently up-regulated cluster in the late time point (red spot upper left, Fig. 8B). This demonstrates once more that one substance may induce distinctly different effect profiles depending on time and concentration. Furthermore, if such profiles were indeed measured in an environmental sample, we would only be able to link these profiles to a diuron exposure with dynamic and concentration-dependent information as supplied by the CTR model.

Certainly, regression models as they are used in our study also have clear limitations. So far the regression model describes responses for a specific exposure setting. Other experimental settings may require refining the model. When, e.g., temporal variation in exposure regimes becomes relevant for extrapolation, the development of models explicitly integrating kinetic and dynamic processes would be desirable (e.g., [78,79]). Data demands regarding time- and dose-resolved observations have restricted their application so far. We see our approach with regard to experimental design and analytical pipeline, therefore, as a step on the avenue to advanced dynamic modelling, which could further progress towards mechanistic models.

Finally, the improved comparability that we discuss here eases consistent interpretation in a toxicological context. As demonstrated for the response of toxnode 1119 in Fig. 4, toxnode responses cannot only be described qualitatively ("is regulated significantly after exposure to substance X") but also quantitatively with the help of estimated parameter values like *S*_max_ or *t*_max_ describing the concentration- or time-related response. This allows, for the first time, toxicogenomic processes to be linked with toxicokinetic measurements (as shown in Fig. 7), thus separating toxicokinetic from toxicodynamic processes. In our case study we found a significant impact of toxicokinetic properties of the substances on the dynamics of various toxicogenomic responses, which is discussed below in more detail. It also helped to identify those responses that seem to be independent of toxicokinetics and rather related to the developmental stage (e.g., regulation of clusters "Trae" or "John").

### Case study: compound effects in the zebrafish toxicogenomic universe

We found that different compounds with a known identical molecular target still show individual toxicogenomic response patterns on the ZFE transcriptome. This goes in line with earlier studies investigating toxicogenomic fingerprints, e.g., in rat liver tissues [9] or ZFEs [10], which reported toxicogenomic profiles to turn out specific for compound, concentration and exposure duration. Advancing from the compound and treatment-specific response barcodes for a few selected transcripts [10], we obtained concentration- and time-resolved, transcriptome-wide toxicogenomic fingerprints for each compound, which can be comprehensively compared between substances.

The clustering of genes into common toxnodes and clusters of the ZTU as illustrated above is indicative for jointly regulated processes, functionally related proteins, or tissue/cell type specificity of genes in the ZFE. Simultaneously, we obtain information about the compound-specific characteristics of the response from the estimated model parameters. In order to illustrate the added value of the approach, we discuss some of the insights we gained about unspecific toxicogenomic responses in the ZFE and common key responses induced by the 2 COX inhibitors.

#### Unspecific key responses

By combining information on model parameter values with functional information on nodes and clusters we can distinguish between specific and unspecific effects. For example, we identified cluster "Trae" and cluster "John" to be related to lens and pancreas development, respectively. While "Trae" is regulated early with diclofenac and diuron exposure, "John" is regulated late with diuron and naproxen exposure. The *t*_max_ for the significantly down-regulated nodes in these clusters were identical for the respective 2 compounds and independent from the different chemical uptake kinetics. This is in contrast to responses for which *t*_max_ was in line with internal substance kinetics (e.g., the induction of the transcription factor *nfe2l2b*). The *t*_max_ values of cluster "Trae" were estimated at ∼6−8 hpe, which equals 30−32 hpf. Cluster "Trae" contains >20 genes coding for crystallin protein subunits that are important for lens development in ZFE, which happens between 16 and 96 hpf [80, 81]. Those transcripts were found to be commonly down-regulated in response to various chemical exposures in earlier studies [18, 82], as well. Cluster "John" is down-regulated at 72 hpe and contains genes coding for different proteolytic enzymes of the pancreas. Indeed, the pancreas development in ZFE starts rather late, between 36 and 72 hpf [83], and a disruption of pancreas development in ZFE by exposures to different chemicals has also been reported in several studies before (e.g., [65,84, 85]).

By comparing our raw data of treatments and controls for these 2 clusters we found that assuming a delay or inhibition of development due to the chemical treatment may explain the down-regulation of transcriptional activity (Fig. S7). The combination of comparing our model parameters between the different exposures, the enrichment of genes in the ZTU, as well as the confirmation by available reports on down-regulation due to chemical exposures let us conclude that these effects on pancreas and lens development are potentially independent of the compound, indicating a developmental delay due to general stress. Whether these delays manifest in adverse outcome is potentially rather a matter of exposure concentration and time than of the specific mode of action of a compound.

#### Key responses induced by COX inhibitors

**Figure 9:**
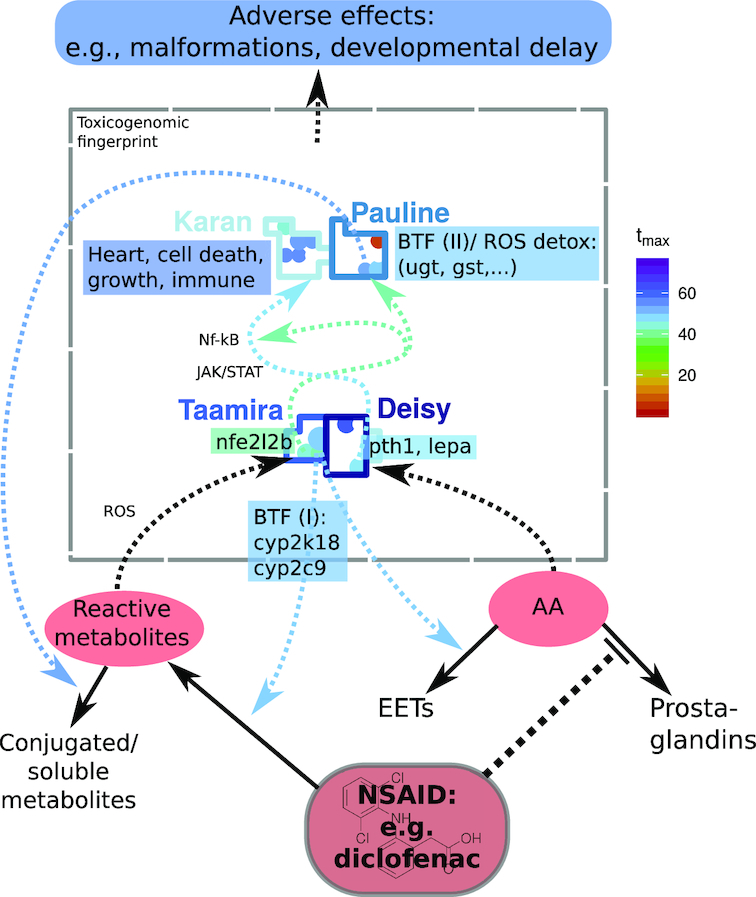
Hypothesized key responses of COX inhibitors in the toxicogenomic fingerprint of diclofenac. Putative causal connections between responses are indicated by dashed arrows; solid arrows indicate transformation reactions, and array color represents estimated *t*_max_ for diclofenac. AA: arachidonic acid; BTF: biotransformation (phase I/II); EETs: epoxyeicosatrienoic acids; NSAID: non-steroidal antiinflammatory drug; ROS detox: detoxification of reactive oxygen species. Note: the plot shows a simplified/idealized version of the toxicogenomic fingerprint, the indentified responses, and their connections.

Two of the investigated model compounds, diclofenac and naproxen, are known to affect the same molecular target, namely, COX. In order to identify key responses of this compound group we studied the patterns commonly evoked by these compounds. We discuss the identified key responses in the context of known molecular effects of COX inhibitors. Our findings and hypothesized molecular key responses are summarized and illustrated in Fig. 9.

##### Metabolism and biotransformation

A predominant response in the fingerprints of diclofenac and naproxen is the up-regulation of metabolic enzymes mainly in cluster "Taamira". For example, we observed an early and strong induction of *cyp2c9* and *cyp2k18* with a *t*_max_ of 48 hpe for diclofenac and 69 hpe for naproxen. We assume that these enzymes are involved in the phase I biotransformation (BTF1) of diclofenac and naproxen. This may lead to an increased production of reactive metabolites leading to liver injury [30]. Additionally, we hypothesize that an inhibition of COX, which transforms AA to prostaglandins [53], leads to an accumulation of AA. This might induce COX-independent branches of the AA pathway, e.g., the production of epoxyeicosatrienoic acids (EETs) [86]. This reaction is known to be catalyzed, among others, by enzymes of the CYP2C family [87]. Therefore, Cyp2c9 and Cyp2k18 may be involved in the biotransformation of the COX inhibitors diclofenac and naproxen themselves, but also in the production of EETs from AA being accumulated due to COX inhibition.

Besides induction of BTF1, glucuronosyltransferases *ugt1* and *ugt5* were induced earlier than many other significant responses in our study. These enzymes were shown to be involved in the phase II biotransformation (BTF2) of diclofenac in humans [88] and fish [89]. Additionally, they can lead to accumulation of acyl glucuronides and subsequent formation of protein adducts [90]. Together with an increase in reactive metabolites from BTF1 this is discussed to be a molecular key event in the AOP of COX inhibitors, leading to mitochondrial dysfunction, impairment of ATP synthesis, apoptosis, and tissue damage, and eventually causing liver and cardiovascular diseases (reviewed in [30]). The occurrence of the initial key events of biotransformation is indicated by the affected genes and toxnodes in our study. The observation of biotransformation being among the first observed key responses in time might indicate that later observed responses are mediated by metabolites instead of the parent compounds or by secondary effects such as an accumulation of AA.

##### Oxidative stress response

Next to biotransformation, glucuronosyltransferases are also associated with an oxidative stress response, which is another key response in the fingerprints of the COX inhibitors in our study. Genes associated with oxidative stress are mainly found in cluster "Pauline" and are potentially induced by the transcription factor NRF2, whose zebrafish orthologue *nfe2l2b* is induced early within cluster "Taamira" [65, 66]. AA-induced NRF2-dependent gene transcription has been reported in brain cells [91] whereas the induction of NRF2 has been shown to prevent toxicity of AA in human liver cells [92]. Next to *ugt1*, several genes for oxidoreductases and glutathione metabolism were specifically induced with the COX inhibitors in cluster "Pauline".

We confirmed the assignment of cluster "Pauline" to oxidative stress response with a dataset from the background data of the ZTU. Paraquat, a herbicide known to induce oxidative stress [93], was investigated using the ZFE by Driessen et al. [94]. We plotted the transcriptome response on the ZTU (Fig. S8A) and found cluster "Pauline" induced as well as 1 node in cluster "Bradley". Cluster "Bradley" is enriched for respiratory electron transport (Table S5). The induction of these clusters with paraquat exposure confirms the biological meaningfulness of the ZTU. However, it only provides a snapshot without evidence on potentially related nodes and clusters or any possibility of extrapolation. Nevertheless, it gives a strong indication of a key response of oxidative stress induced by diclofenac and naproxen. Indeed, oxidative stress has been discussed as an adverse effect of diclofenac and other NSAIDs before [95, 96].

##### Induction of regulatory hormones

Another key response in the COX inhibitor fingerprints was the up-regulation of the regulatory hormones Leptin α and Pth1a in cluster "Deisy". The induction of leptin together with *cart3* in the same cluster might be due to a stress-related change in energy metabolism [55] or due to AA accumulation [52]. Leptin induces a MAPK pathway (JAK/STAT), which is an activator of NF-κB [59, 97]. Furthermore, PTH was shown to regulate the ligand of NF-κB in mammalian osteocytes [98, 99]. Indeed, we identified the genes and nodes of the subsequently induced clusters "Karan" and "Farajallah" to be targets of these pathways [100] (also see Results section and Fig. 9). The interaction of COX inhibitors with these pathways as cyclooxygenase−independent effects was reported by Tegeder et al. [101]. Indeed, little is known about the molecular interactions of AA, leptin, and PTH. A role of AA in leptin signalling and hepatic energy metabolism has been described before [102]. Furthermore, it has been reported that leptin can induce the secretion of PTH (e.g., [103]). However, a strong co-expression of the 2 hormones as observed in our study has not been reported before and indicates a joint action in response to chemicals or stress in ZFE.

Interestingly, when projecting the toxicogenomic fingerprint of BDE-47, measured by Xu et al. [104], on the ZTU, we see a similar fingerprint as with naproxen and diclofenac (Fig. S8B). We find, among others, toxnode 1062 containing *lepa* and *pth1a*, as well as some other toxnodes of the clusters "Taamira" and "Karan" up-regulated with BDE-47. A study by [105] showed that polybrominated diphenyl ethers as well as polychlorinated biphenyls cause a release of AA in rat neurons. This might indicate that the obtained phenotypes, which were similar with BDE-47 exposure [106] to the ones we observed with the 2 COX inhibitors (Fig. S2), show that tail malformations, spinal curvature, small eyes, and edema are related or initially caused by a disturbance of the AA metabolic pathway.

In summary, using a comparative analysis of the knowledge gained from the ZTU combined with compound-specific parameter values from the CTR model, we could identify and characterize key responses to diuron, diclofenac, and naproxen exposures in the ZFE. Furthermore testable hypotheses about the sequence of key responses and their connections to toxicokinetic, toxicodynamic, or developmental processes were generated as illustrated in Fig. 9.

## Summary and Implications

With our experimental design and analysis pipeline we derived dynamic toxicogenomic fingerprints. The applied regression model allows inference on the concentration as well as the time scale to conditions not measured. It also proves helpful in separating toxicokinetic from toxicodynamic processes. The ZTU introduced here allows toxicogenomic fingerprints to be aggregated on a map. Taken together, this novel approach facilitates comparison between different fingerprints and different studies as well as between the responses in a single fingerprint. We see several implications that may arise from these results.

### 

#### The toxicogenomic universe as source for biological hypothesis building and gene selection for high-throughput approaches

We demonstrate in this study that the clustering of genes in the toxicogenomic universe can be used to derive biological hypotheses about co-expressed genes. Furthermore, the toxicogenomic universe can be used to support gene selection for reduced transcriptome approaches. There have been efforts to use reduced transcriptome arrays, implying much lower costs, which allows more extensive datasets to be obtained. A reduced mouse transcriptome array was recently used to measure perturbation profiles of >20,000 substances in different cell lines [107], generally demonstrating the power of high-throughput molecular approaches for large-scale assessments. Recently, a reduced array for the zebrafish transcriptome was also suggested [108] including selected genes to represent a range of biological pathways. This selection, however, was not based on zebrafish experimental data but focused on orthologues of genes known to be important in mammalian toxicology. Therefore, an alternative approach to design a reduced zebrafish array could comprise the selection of a representative gene for each toxnode of the ZTU or the selection of genes within a specific region of interest in the ZTU.

#### The CTR model to enhance molecular databases

The scope of functional annotation databases could substantially improve with the inclusion of quantitative exposure and effect information, e.g., as derived from concentration response relationships as shown in our study. We demonstrated that it is possible to quantitatively describe a majority of toxicogenomic responses with a universal regression model. This could be used to enhance annotation databases, such as GO [41], Molecular Signatures Database (MSigDB) [109], or Comparative Toxicogenomics Database [110], which, so far, focus on qualitative information about responses.

#### Dynamic toxicogenomic fingerprints for read-across and elucidation of adverse outcome pathway(s)

The dynamic toxicogenomic fingerprints foster read-across approaches between chemicals by providing enhanced comparability. This could improve application in chemical hazard assessment and effect-based environmental monitoring. The inference and comparison of toxicogenomic universes for different species could furthermore aid in cross-species extrapolation.

The fingerprints can also help in elucidating key events of an AOP. For this we can use previously derived functional knowledge about responding toxnodes or clusters to draw conclusions about the effects of other substances, as shown in Fig. S8. In this study we have shown the identification and comparative characterization of key responses for 2 COX inhibitors. We see this as a helpful starting point for informing the development of AOPs. In this regard, our approach might also help in mechanism-based risk assessment.

#### Molecular mixture toxicology

Finally, mixture assessment relevant in environmental monitoring becomes possible with the help of the suggested CTR model. In the context of environmental monitoring of substances, toxicogenomic fingerprints of environmental samples should in principle be suitable to be compared with fingerprints of single substances. However, it remains to be clarified whether (i) individual toxicogenomic fingerprints can also be recovered in a mixture context and (ii) how fingerprints of different substances combine in a qualitative and quantitative way. Existing mixture concepts such as concentration addition or independent action could guide the evaluation of mixture responses and the identification of additive or non-additive behaviour [111]. The dynamic fingerprints inferred here lend themselves for such hypothesis-based experimentation.

## Methods

We briefly outline the experimental procedure and analysis steps performed in our study. We additionally prepared an extensive supplementary Methods file containing detailed information about the experimental procedure (exposure, RNA extraction, measurement of toxicokinetics) and the data analysis pipeline (data import, quality control, creating the toxicogenomic universe, regression modelling). It also includes the R code used for generating the results shown in this study. The results have (in part) been computed at a high-performance computing cluster at the Helmholtz Centre for Environmental Research.

### Exposure of ZFEs to 3 model compounds

ZFEs were exposed to diuron, diclofenac sodium salt, and naproxen sodium salt in 5 different concentrations between LC_0.5_ and LC_25_ from 24 hpf. At 6 time points between 3 and 72 hpe RNA was extracted and the transcriptome was measured using Oaklabs (Berlin, Germany) Zebrafish XS Microarrays.

### Import, quality control, and preprocessing of data

The median fluorescence for each array spot was extracted by the Agilent Feature Extraction Software (Version 11.5.1.1). All further analysis was performed in R (Version 3.4.3 [112]).

Quality control was performed by checking density distributions and Euclidean distance between samples. Similar to the procedure recommended by Kauffmann and Huber [113] we checked 4 quality metrics: Kolmogorov-Smirnov test statistics, sum of all expression values per array, interquartile range (IQR), and Euclidean distance. Samples with 1 of the metrics outside of a range between 25% and 75% quantile ± 3 × IQR (1 × IQR for Euclidean distance) were removed from further analysis. Processed intensity values were normalized using the "cyclic loess" method.

After normalization all data were transformed by log_2_. Subsequently, the median expression values of replicate probes were calculated. If replicates of a probe were present on the array, only replicates that had not been flagged for poor quality during the feature extraction process (due to inhomogeneous spots or background) were considered. Laboratory batch effects in the diclofenac experiment were removed using the R package sva [114].

Transcript abundance changes drastically for many transcripts during the course of embryo development, even without exposure to a chemical (cf. Fig. S9). At this point the effect of the chemical was of main interest. Therefore, the developmental effect on the transcriptome was removed by normalizing all transcript-level values against the control of the respective time point. This resulted in logFC data for all experimental conditions.

### Inferring the toxicogenomic universe

The spline-smoothed logFC data from our experiments were combined with the logFC data from previously published ZFE microarray data. Data from public databases were selected, downloaded, and processed in a semi-automatic workflow, which is accessible via protocols.io [24]. All included microarray platforms were annotated to the most recent zebrafish genome (GRCz11), and Ensembl database Version 93 [25].

The Grubb’s test ([115], implemented in R package outliers) was used iteratively to remove outliers from the group of data points of each probe (points were removed until *P* ≥ 0.001). This resulted in 0.2−0.3% of measurements being removed for each substance. Then, a thin plate spline was fitted to the treatment conditions of each probe using the R package mgcv [116] and logFC values for each measurement condition were extracted. The R package kohonen [117] was used to train the SOM on a 60 × 60 rectangular grid. The initial learning rate was set comparably high in order to make the node codes quickly adjust to the assigned transcript behaviour. The properties of the map and the learning algorithm are summarized in Table 2.

**Table 2.** Properties of SOM learning

Parameter	Value
Learning rate	0.8–0.005
Neighborhood radius	40 to –40
Neighborhood function	Gaussian
Epochs	1,000
Distance function	Manhattan distance

The outcome of this step is a 60 × 60 grid of 3,600 toxnodes. Each gene present in our dataset is permanently assigned to 1 toxnode, while each toxnode contains genes that behave similarly across all exposure conditions. We used the R package mclust [118] to determine an optimal cluster number for subgrouping (see supplementary Methods for more details). The package randomNames [119] was used to automatically name the clusters.

For identification of over-represented annotations within the clusters, we used the package clusterProfiler [120] together with annotation from the databases ZFIN [38], InterPro [39], Reactome [40], and GO [41, 42]. We applied correction for multiple testing with the Benjamini-Hochberg method and a *P*-value cut-off of 0.05.

### Parameter estimation of mobi-CTR model

Normalized logFC data (not the spline fit) were used as input data for parameter estimation. Measured data from all probes assigned to one node of the SOM were used to estimate one parameter set for each node and substance (i.e., experimental replicates and transcriptional replicates/groups of transcripts were treated as belonging to a single distribution here). The Grubb’s test ([115], implemented in R package "outliers") was used iteratively to remove outliers from the group of data points (points are removed until *P* ≥ 0.001) in one node. This resulted in removal of 0.1−0.2% of data points for each substance. The extreme values across all samples and experimental conditions were determined for each node. Then, the dataset for each node was used to estimate parameters for the mobi-CTR using the shuffled complex evolution algorithm assuming up-regulation. This estimation procedure was repeated 3 times with 3 different random seeds and 10 complexes each. The best model was afterwards selected using AICC. The same procedure was repeated assuming down-regulation. The best up-regulation model and the best down-regulation model were again compared using AICC and the best-fit model subsequently used for a quantitative description of the node. We used the R implementation of the algorithm shuffled complex evolution (described in [121]) in the package hydromad [122]. For a global parameter estimation method like shuffled complex evolution, parameter boundaries should be defined carefully. To limit the fitted parameter values to a range that makes sense in the context of the experiment, boundaries were set as described in the supplementary Methods file.

### Fingerprint browser

To ease the exploration of the toxicogenomic fingerprints in the context of the toxicogenomic universe, we created an online application [47]. The app was created using R in combination with the package shiny [123].

## Supplementary Material

giz057_GIGA-D-19-00015_Original-Submission

giz057_GIGA-D-19-00015_Revision-1

giz057_GIGA-D-19-00015_Revision-2

giz057_Response_to_Reviewer_Comments_Original_Submission

giz057_Response_to_Reviewer_Comments_Revision-1

giz057_Reviewer_1_Report_Original_Submission -- Leo Lahti 2/27/2019 Reviewed

giz057_Reviewer_2_Report_Original_Submission -- Katharine Horzmann, DVM, Ph.D., MPH 2/27/2019 Reviewed

giz057_Reviewer_2_Report_Revision_1 -- Katharine Horzmann, DVM, Ph.D., MPH 3/24/2019 Reviewed

giz057_Reviewer_3_Report_Original_Submission -- Lisa Truong 3/3/2019 Reviewed

giz057_Reviewer_3_Report_Revision_1 -- Lisa Truong 3/20/2019 Reviewed

giz057_Supplementary_Figures

giz057_Supplementary_Methods

giz057_Supplementary_Tables

## Supplementary Methods

A supplementary Methods file was compiled containing extensive documentation about experimental and data analysis workflow including all R code needed to reproduce the results. The file was created with the help of knitR [124].

## Availability of source code and requirements

Functions used for the analysis were compiled into the R package toxprofileR, which is available via a git repository:

- Project name: toxprofileR
- Project home page: https://git.ufz.de/itox/toxprofileR
- Operating system(s): Platform independent
- Programming language: R (>3.4.3)
- License: GNU GPL Version 3
- RRID: SCR_017027

Furthermore, we provide an interactive online tool for exploration of the datasets obtained in our study:

- Project name: Toxicogenomic Fingerprint Browser
- Project home page: https://webapp.ufz.de/itox/tfpbrowser/
- Operating system(s): Platform independent
- Programming language: R (>3.4.3)
- License: GNU GPL Version 3
- RRID: SCR_017028

## Availability of supporting data and materials

The microarray data of this study have been deposited in NCBI’s GEO [125] and are accessible through GEO Series accession No. GSE109496 (https://www.ncbi.nlm.nih.gov/geo/query/acc.cgi?acc=GSE109496). The functions used for analyses and figures have been compiled in the R-package *toxprofileR*, which is available via (https://git.ufz.de/itox/toxprofileR/).

An interactive online tool for exploration of the datasets obtained in our study can be accessed via https://webapp.ufz.de/itox/tfpbrowser/.

## Additional files

Supplementary Methods. Extensive documentation about experimental and data analysis workflow including all R code needed to reproduce the results. Source files (R markdown) are provided in the GigaDB repository.

Figure S1. Experimental design for transcriptome experiments applied in our study

Figure S2. Toxicodynamic fingerprint for diuron projected on the toxicogenomic universe

Figure S3. Toxicodynamic fingerprint for diclofenac projected on the toxicogenomic universe

Figure S4. Histogram of AICC weights of fitted mobi-CTR model in comparison with null model (A: diuron; B: diclofenac; C: naproxen) and spline fit (D: diuron, E: diclofenac, F: naproxen)

Figure S5. A: Demonstration of significant effect level calculation for a toxnode

Figure S6. Morphological effects of ZFE exposure to A: diuron (29.44 μM), B: diclofenac (7.36 μM), C: naproxen (309.14 μM) for 3 different treatment periods (24−48, 24−72, 24−96 hpf).

Figure S7. Regulation of genes in the pancreas cluster "John" (A−C) and crystallin cluster "Trae"

Figure S8. Toxicogenomic fingerprints projected on the toxicogenomic universe

Figure S9. Multidimensional scaling plot of all transcriptome samples for diuron, diclofenac, and naproxen exposure between 24 and 96 hpf

Table S1. Table of datasets included in the toxicogenomic universe

Table S2. Toxnode and cluster assignment in the toxicogenomic universe

Table S3. Enriched GO annotation for toxnode clusters

Table S4. Enriched Interpro annotation for toxnode clusters

Table S5. Enriched Reactome annotation for toxnode clusters

Table S6. Enriched ZFIN annotation for toxnode clusters

Table S7. Significantly affected toxnode with diuron exposure

Table S8. Significantly affected toxnode with diclofenac exposure

Table S9. Significantly affected toxnode with naproxen exposure

## Abbreviations

AA: arachidonic acid; AHR: arylhydrocarbon receptor; AICC: small-sample Akaike information criterion; AOP: adverse outcome pathway; ATP: adenosine triphosphate; BTF1: phase I biotransformation; BTF2: phase II biotransformation; CAS RN: Chemical Abstracts Service Registry Number; COX: cyclooxygenase; CTR model: concentration and time-dependent response model; df: dilution factor; EET: epoxyeicosatrienoic acid; GEO: Gene Expression Omnibus; GO: Gene Ontology; hpe: hours post exposure; hpf: hours post fertilization; IQR: interquartile range; LC: lethal concentration; logFC: log_2_(fold change); MAPK: mitogen-activated protein kinase; MSigDB: Molecular Signatures Database; NCBI: National Center for Biotechnology Information; NF-κB: nuclear factor κ-light-chain-enhancer of activated B cells; NSAID: non-steroidal anti-inflammatory drug; PTH: parathyroid hormone; ROS detox: detoxification of reactive oxygen species; SOM: self-organizing map; ZTU: Zebrafish Embryo Toxicogenomic Universe; ZFE: zebrafish embryo; ZFIN: Zebrafish Model Organism Database.
