# Supplementary material for: Map and model—moving from observation to prediction in toxicogenomics
Source: Gigascience. 2019 May 29;8(6):giz057. doi: 10.1093/gigascience/giz057 (PMC6539241; doi:10.1093/gigascience/giz057)
Supplement: giz057_GIGA-D-19-00015_Revision-1 [file giz057_giga-d-19-00015_revision-1.pdf]

GigaScience
Map and Model - moving from observation to prediction in toxicogenomics
--Manuscript Draft--

**Manuscript Number:**	GIGA-D-19-00015R1
**Full Title:**	Map and Model - moving from observation to prediction in toxicogenomics
**Article Type:**	Research
**Funding Information:**	Deutsche Bundesstiftung Umwelt (AZ: 20014/350) — Mr. Andreas Schüttler<br>FP7 Environment (603437) — Mr. Andreas Schüttler, Dr. Wibke Busch
**Abstract:**	Background: Chemicals induce compound specific changes on the transcriptome of an organism (toxicogenomic fingerprints). This provides potential insights about the cellular or physiological responses to chemical exposure and adverse effects, which is needed in assessment of chemical related hazards or environmental health. In this regard, comparison or connection of different experiments becomes important when interpreting toxicogenomic experiments. Due to lack of capturing response dynamics, comparability is often limited. In this study, we aim to overcome these constraints.<br>Results: We developed an experimental design and bioinformatic analysis strategy to infer time and concentration-resolved toxicogenomic fingerprints. We projected the fingerprints to a universal coordinate system (toxicogenomic universe), based on a self-organizing map of toxicogenomic data retrieved from public databases. Genes clustering together in regions of the map indicate functional relation due to co-expression under chemical exposure. To allow for quantitative description and extrapolation of the gene-expression responses we developed a time and concentration-dependent regression model. We applied the analysis strategy in a microarray case study exposing zebrafish embryos to three selected model compounds including two cyclooxygenase inhibitors. After identification of key responses in the transcriptome we could compare and characterise their association to developmental, toxicokinetic, and toxicodynamic processes using the parameter estimates for affected gene clusters. Furthermore, we discuss an association of toxicogenomic effects with measured internal concentrations.<br>Conclusions: The design and analysis pipeline described here could serve as a blueprint for creating comparable toxicogenomic fingerprints of chemicals. It integrates, aggregates, and models time and concentration-resolved toxicogenomic data.
**Corresponding Author:**	Andreas Schüttler<br><br>GERMANY
**Corresponding Author Secondary Information:**	
**Corresponding Author's Institution:**	
**Corresponding Author's Secondary Institution:**	
**First Author:**	Andreas Schüttler
**First Author Secondary Information:**	
**Order of Authors:**	Andreas Schüttler
	Rolf Altenburger
	Madeleine Ammar
	Marcella Bader-Blukott
	Gianina Jakobs
	Johanna Knapp
	Janet Krüger
	Kristin Reiche

Powered by Editorial Manager® and ProduXion Manager® from Aries Systems Corporation

	Gi-Mick Wu
	Wibke Busch
**Order of Authors Secondary Information:**	
**Response to Reviewers:**	We thank the editor and the three reviewers for their constructive and valuable remarks.<br>We addressed the suggested revisions in our manuscript and supply specific point-by-point answers below.<br><br>###<br>Response to editor:<br><br>“please register any new software application in the SciCrunch.org database to receive a RRID (Research Resource Identification Initiative ID) number, and include this in your manuscript. This will facilitate tracking, reproducibility and re-use of your tool.”<br><br>Response: Agree - We have registered toxprofileR and the Toxicogenomic Fingerprint Browser at the SciCrunch.org database and added the RRIDs in the manuscript.<br><br>Changes made in the manuscript:<br>Added RRIDs in the section "Availability of source code and requirements".<br><br>###<br>Responses to reviewer #1:<br>1.1 "Some of the technical implementation details could be potentially omitted in the scientific reporting part, in order to make it easier to follow. However, I would not request changes to the work regarding this."<br><br>Response: We acknowledge the technical heaviness of our study and double checked the scientific reporting part for technical details that could be omitted in favor of readability. However, we did not find any parts which would be dispensable in our view.<br><br>Changes made in the manuscript:<br>none<br><br>--<br>1.2 "Adding the Rmd source files in the supplements should be sufficient for transparency"<br><br>Response: Agree<br><br>Changes made in the manuscript:<br>.Rmd source files were added together with a source file for the plots and tables in a .zip file in the supplementary data and referenced in the supporting data section<br><br>--<br>1.3 “If it possible, I would propose switching from GPL to MIT license”<br><br>Response: We completely agree with the reviewer, that a more permissive licence would be desirable. However the work is partly based on GPL licensed packages, why this option is unfortunately not available.<br><br>Changes made in the manuscript:<br>none<br><br>###<br>Responses to reviewer #2:<br><br>2.1 "One suggestion would be to make the type of information provided in the supplemental methods more explicit in the manuscript methods section."

Response: Agree

Changes made in the manuscript:
Addition to the Methods part: "[We additionally prepared an extensive supplementary methods file] (supplementary_methods.html). This file contains [detailed information about the experimental procedure] (exposure, RNA extraction, measurement of toxicokinetics) [and the data analysis pipeline] (data import, quality control, creating the toxicogenomic universe, regression modelling)[...]

2.2 "I believe that it is difficult to adequately control for the amount of variability in the publically available transcriptomic datasets, which is why the limitations of the analysis need to be clearly stated. Major limitations of the bioinformatic analysis strategy described include the lack of consideration of other factors that may cause variation in transcriptomic results. Zebrafish embryos are sensitive to environmental factors, such as incubation temperature, and in toxicological studies, the time exposure is initiated is thought to be important. Based on the description of methods, length of exposure was taken into consideration, but time of initial exposure in the integrated studies seems not to have been accounted for. As reported in the manuscript many developmental toxicity assays start exposure immediately after fertilization, although 4-6 hours post fertilization is also commonly seen. The exposures in this study start at 24 hours post fertilization, which may mean that early gene expression changes were missed due to the later dosing period. The difference in dosing time also makes it difficult to compare across post exposure time points, because embryos may be different at different stages of development."

Response: Clarification - We completely agree with the reviewer that there can be many different factors (incubation temperature, exposure start and duration, pH, light conditions, genetic background, etc.) which may influence the transcriptome profiles of zebrafish embryos to different degree. Some of these factors do differ substantially between the experiments (which we discussed in detail in Schüttler et al. 2017). In the presented approach, however, these factors should not influence the analysis. This is because we use fold-change data which is normalized to the control of the respective experiment. Moreover, we did not compare different studies here, but used the normalized gene expression information for clustering genes within the self-organizing-map approach. Certainly, more data and data from experiments with different settings, chemicals, etc. would potentially influence the result of this clustering. We tried to clarify this in the manuscript. Additionally, we acknowledged in the discussion that co-expression may also differ between different treatments.

Changes made in the manuscript:
Addition to Data analysis part: "All datasets were normalised against the respective control of the same experiment"
Addition to Discussion: "With the help of the SOM we can retrieve information about co-expression of genes from publicly available toxicogenomic datasets and include this information into our analysis irrespective of differing experimental designs and occurrence of missing data."
Addition to Discussion: "Additionally, co-expression of genes is not necessarily consistent across different perturbations [68]. This would not be captured in the dynamic toxicogenomic fingerprints as shown here."

2.3 "The authors describe an incubation temperature of 26 degrees Celsius for this study, but 28.5 degrees Celsius is a more common incubation temperature."

Response: We agree that incubation temperature may influence the development of the zebrafish embryos. However, we mainly expect the fish to develop slower in this temperature range, which should not influence the found toxicogenomic responses. In our study we chose an incubation temperature of 26 degrees celsius as it is used in OECD guideline No. 236 (OECD, 2013).

OECD (2013), Test No. 236: Fish Embryo Acute Toxicity (FET) Test, OECD Guidelines for the Testing of Chemicals, Section 2, OECD Publishing, Paris,

https://doi.org/10.1787/9789264203709-en.

Changes made in the manuscript:
none

2.4 "It should also be noted that the diuron exposure medium contained 0.1% methanol, and it does not appear that a 0.1% methanol solvent control was used in addition to a water control."

Response: Clarification - We clarified in the supplemental methods file, where we describe details of the exposure, that we indeed used solvent controls for the diuron exposure.

Changes made in the manuscript:
Addition in the supplemental methods file: "For the diuron exposure we used 0.1 % methanol as solvent control."

2.5
"Although the manuscript does provide very detailed descriptions of design and on the analysis of data from the case study, the limitations of the model are not well addressed."
"[...]more discussion is needed into the limitations of the model"
"As mentioned previously, I think the manuscript needs to better address limitations. Currently, the analysis and discussion are slightly on the overly positive side and need to be balanced."
"a weakness is the possible overgeneralization of different experimental design or environmental conditions associated with zebrafish embryos (incubation temperature, time exposure is initiated)"

Response:
Agree

Changes made in the manuscript:
i) Addition in discussion (Integration and aggregation of toxicogenomic responses): "A SOM shows limitations in capturing complex interactions compared to the more 'flexible' networks in the aforementioned studies (i.e. connections between nodes are only formed between direct neighbors on the map). Therefore some relevant interactions between transcriptomic units might get lost with the SOM. Additionally, co-expression of genes is not necessarily consistent across different perturbations [68]. This would not be captured in the dynamic toxicogenomic fingerprints as shown here. However,SOMs have the advantage of [...]"
ii) Addition in discussion (Modelling of time and concentration dependence): "For example, toxnodes showing a biphasic response on the concentration scale would not be accurately captured".
iii) Addition in discussion (Modelling of time and concentration dependence): "So far the regression model describes responses for a specific exposure setting. Other experimental settings may require refining the model."

2.6 "I would also like to see further discussion on some of the points in the Summary and Implications section. The "Dynamic toxicogenomic fingerprints for read-across and elucidation of adverse outcome pathway(s)" and "Molecular mixture toxicology" subsections could be further expanded: One of my questions from the "Dynamics" subsection is how can the existing Universe be used to evaluate a novel compound"

Response: Agree (also compare comment 3.2)

Changes made in the manuscript:
Addition to Implications: "For this we can use previously derived functional knowledge

about responding toxnodes or clusters to draw conclusions about the effects of other substances, as exemplarily shown in Figure S8."

Addition to Discussion: "The supplementary methods file (supplementary_methods.html) demonstrates how to use our supplied R-package toxprofileR and the toxicogenomic universe to infer toxicogenomic fingerprints of additional compounds."

2.7 "The "Molecular mixture" subsection lacks any discussion of interactions such as synergy, additive effects, or antagonistic effects. This section needs further discussion; alternatively, it would not hurt the manuscript if it were removed."

Response: Agree

Changes made in the manuscript:
Addition to Implications: "Existing mixture concepts such as concentration addition or independent action could guide the evaluation of mixture responses and the identification of additive or non-additive behaviour [109]"

2.8 "I also wonder about how well a reduced array with only one gene from each toxnode would perform and if it would have any utility. I could see reduced arrays within toxnodes being more useful for interrogation of specific mechanisms within a general effect."

Response: Clarification - We agree that the utility of reduced, comprehensive transcriptome arrays has to be proven. However, we observe an increased development and evaluation of reduced transcriptome arrays. We propose that the toxicogenomic universe could be helpful in the array design. We acknowledge the reviewers idea of reduced arrays withing toxnodes or clusters and added this to the manuscript.

Changes in the manuscript:
Addition to Implications: "[Therefore, an alternative approach to design a reduced zebrafish array could comprise the selection of a representative gene for each toxnode of the Zebrafish Embryo Toxicogenomic Universe (ZTU)] or the selection of genes within a specific region of interest in the ZTU."

2.9 "[...] it should be noted that no internal standard or spike in was used in the measurement of diuron, diclofenac, or naproxen in zebrafish embryo/larval tissues and it is not mentioned if the concentration of drug was confirmed in the exposure media"

"The toxicokinetics portion also is weaker than it could be due to only direct analysis of compounds only without accounting for internal or spiked controls or for active metabolites. These weaknesses should be acknowledged"

Response: Agree and clarification - we did not use a spike-in for calibration and clarified this now in the supplementary methods. We confirmed the concentration of drug in the exposure media and added this information to the supplementary methods file now.

Changes in the manuscript:
Addition in supplementary methods file (section toxicokinetics): "A calibration curve was acquired in the respective medium. No additional spike-in or internal standard was used for calibration."
Added plot with measured concentration in exposure medium to supplementary methods file.

##

###
Responses to reviewer #3:

3.1 "The generation of the toxicogenomic universe utilizes only microarrays, which is a predetermined set of probes. I realize the concept is like the Connectivity Maps from the L1000 platform, but how will this platform be expanded upon later in the future? The technology of microarray is slowly growing out of favor and no new probes will be added with the discovery of new genes. This should be discussed in the paper to explain what the next extension will be"

Response: We agree with the reviewer that the microarray technology may phase out eventually. However, our approach is independent of the technology used to measure the transcriptome. We added a statement in the discussion to clarify this.

Changes made in the manuscript:
Addition in Discussion: "While in our study the analysis focused on microarray data, the approach is not limited to microarray data and could also be applied on RNAseq data. This is also demonstrated in Figure S8B showing RNAseq data projected on the toxicogenomic universe."

3.2 "The toxicogenomic universe is provided with little explanation of how someone can adapt all this great work. It would be a disservice to not include an excerpt of how the field of toxicology can use it."

Response: Agree (compare comment 2.6). The R-package supplied with the paper should allow adaption of the toxicogenomic universe and the described methods for future studies. The supplementary methods file can be seen as a manual how to use this. We added a statement in the discussion to clarify this.

Changes made in the manuscript:
Addition to discussion: The supplementary methods file (supplementary_methods.html) demonstrates how to use our supplied R-package \textit{toxprofileR} and the toxicogenomic universe to infer toxicogenomic fingerprints."

3.3 "The shiny app is great to display your own results for the 3 chemicals, which has concentration and time data. However, how will it work with chemicals that are tested at only one concentration or one time point? This is a limitation should be explained."

Response: Clarification - As it is demonstrated in Figure S8 (also see comment 3.2) the toxicogenomic universe can also be used to project profiles of only one concentration or time point. With the help of the toxprofileR package time- and concentration dependent data can be processed to be viewed with the toxicogenomic fingerprint browser.

Changes made in the manuscript:
none

3.4 "However, how does this work if there non-monotonic responses? The chemical selected are known to have responses that are dose dependent, but not every chemical is this case. A discussion should be included on this."

Response: Agree

Changes made in the manuscript (see also Comment 2.5):
Addition in discussion (Modelling of time and concentration dependence): "For example, toxnodes showing a biphasic response on the concentration scale would not be accurately captured".

Powered by Editorial Manager® and ProduXion Manager® from Aries Systems Corporation

----

3.5 "AIC is used for the selection of the model - which is appropriate. Please provide some information on the range of AIC values. Also - was there a likelihood test conducted?"

Response: Clarification - Because AIC is only a relative measure of model fit for a given dataset, the absolute value itself is meaningless (Akaike, 1981). AIC values are only useful for comparing different models fitted one one dataset. We selected the models using AIC, which is the likelihood of a model penalized by its complexity. This also allowed us to compare non-nested models, which cannot be done using a Likelihood Ratio test. For these reasons, we chose not to conduct Likelihood Ratio tests. The range of relative AIC-weights is given in Figure S4.

Akaike, Hirotugu. (1981). Likelihood of a model and information criteria. Journal of Econometrics, 16(1), 3-14. doi: http://dx.doi.org/10.1016/0304-4076(81)90071-3

Changes in the manuscript: none

3.6 "Figure 8 is to small to see anything meaningful. The discussion around it is non-descript and hard to follow"

Response: Agree and Clarification - Figure 8 is not meant to show details of a fingerprint. We rewrote the discussion around Figure 8 to make this more clear.4

Changes in the manuscript:

Updated Figure 8

Changed paragraph discussing Figure 8: "As it is not conceivable that concentrations, selected and measured in experiments, will ever cover the whole range of concentrations relevant for estimating or interpreting environmental effects, measures for extrapolation become relevant. In this regard, the CTR-model allows building hypotheses and predictions about toxicogenomic effects at conditions not measured. For example, with the help of the model we can predict for each toxnode in the ZTU which response we would expect for an environmentally relevant concentration of 0.86 µmol/L of diuron [concentration measured in 75]. This is falling outside the concentration range measured in our study. In Figure 8 the expected logFCs for 16 and 80 hpe are projected on the ZTU. Without investigating the profiles in detail, we can observe that the predictions show distinctly different profiles. A prominently down-regulated cluster can be seen in the early time point (blue spot bottom left) and a prominently up-regulated cluster in the late time point (red spot upper left). This demonstrates once more that one substance may induce distinctly different effect profiles depending on time and concentration. Furthermore, if such profiles were indeed measured in an environmental sample, we would only be able to link these profiles to a diuron exposure with dynamic and concentration-dependent information as supplied by the CTR-model.

3.7 "In the paragraph: "To obtain an overview over the ZTU we grouped the 3600 toxnodes into 118 clusters", please include information on the range of nodes per cluster"

Response: Agree

Changes in the manuscript:
Addition to results part: "The resulting clusters contained between 3 and 93 toxnodes with an average of 30."

Powered by Editorial Manager® and ProduXion Manager® from Aries Systems Corporation

	3.8 "Figure 3- the size of the dot isn't even visible. This should be removed, as there is no point to this."<br><br>Response: Agree<br><br>Changes in the manuscript:<br>Changed figure accordingly<br><br>--<br><br>3.9 "modeling section - In regards to the first sentence - "consideration of finding specific" - that does this refer to and needs more clarification."<br><br>Response: Agree<br><br>Changes in the manuscript:<br>Changed sentence to "The analysis up to this step allows the consideration of findings for each exposure setting in isolation."<br><br>--<br><br>3.10 "Figure 6 legend - clarify "sum(CI)""<br><br>Response: Agree<br><br>Changes in the manuscript:<br>Added "Dotsize represents significant effect level (sumCI, compare Figure S5A)." to figure legend.
Additional Information:	
**Question**	**Response**
Are you submitting this manuscript to a special series or article collection?	No
**Experimental design and statistics**<br><br>Full details of the experimental design and statistical methods used should be given in the Methods section, as detailed in our Minimum Standards Reporting Checklist. Information essential to interpreting the data presented should be made available in the figure legends.<br><br>Have you included all the information requested in your manuscript?	Yes
**Resources**<br><br>A description of all resources used, including antibodies, cell lines, animals and software tools, with enough information to allow them to be uniquely identified, should be included in the	Yes

Methods section. Authors are strongly encouraged to cite Research Resource Identifiers (RRIDs) for antibodies, model organisms and tools, where possible.<br><br>Have you included the information requested as detailed in our Minimum Standards Reporting Checklist?	
**Availability of data and materials**<br><br>All datasets and code on which the conclusions of the paper rely must be either included in your submission or deposited in publicly available repositories (where available and ethically appropriate), referencing such data using a unique identifier in the references and in the “Availability of Data and Materials” section of your manuscript.<br><br>Have you have met the above requirement as detailed in our Minimum Standards Reporting Checklist?	Yes

Manuscript

Click here to view linked References

Click here to access/download;Manuscript;map_and_model_revision1.pdf

OXFORD

(GIGA)^n SCIENCE

GigaScience, 2019, 1–22

doi: xx.xxxx/xxxx
Submitted Manuscript
Paper

PAPER

Map and Model – moving from observation to prediction in toxicogenomics

Andreas Schüttler[1,2,*], Rolf Altenburger[1,2], Madeleine Ammar[1], Marcella Bader-Blukott[1], Gianina Jakobs[1], Johanna Knapp[1], Janet Krüger[1], Kristin Reiche[3], Gi-Mick Wu[4] and Wibke Busch[1]

[1]Department Bioanalytical Ecotoxicology, Helmholtz-Centre for Environmental Research – UFZ, Permoserstr. 15, 04318 Leipzig, Germany and [2]Institute for Environmental Research, RWTH Aachen, Worringerweg 1, 52074 Aachen, Germany and [3]Bioinformatics Unit, Department of Diagnostics, Fraunhofer Institute for Cell Therapy and Immunology, Perlickstr. 1, 04103 Leipzig, Germany and [4]DEVELOP, Helmholtz-Centre for Environmental Research – UFZ, Permoserstr. 15, 04318 Leipzig, Germany

[*]andreas.schuettler@ufz.de

Abstract

Background Chemicals induce compound specific changes on the transcriptome of an organism (toxicogenomic fingerprints). This provides potential insights about the cellular or physiological responses to chemical exposure and adverse effects, which is needed in assessment of chemical related hazards or environmental health. In this regard, comparison or connection of different experiments becomes important when interpreting toxicogenomic experiments. Due to lack of capturing response dynamics, comparability is often limited. In this study, we aim to overcome these constraints. **Results** We developed an experimental design and bioinformatic analysis strategy to infer time and concentration-resolved toxicogenomic fingerprints. We projected the fingerprints to a universal coordinate system (*toxicogenomic universe*), based on a self-organizing map of toxicogenomic data retrieved from public databases. Genes clustering together in regions of the map indicate functional relation due to co-expression under chemical exposure. To allow for quantitative description and extrapolation of the gene-expression responses we developed a time and concentration-dependent regression model. We applied the analysis strategy in a microarray case study exposing zebrafish embryos to three selected model compounds including two cyclooxygenase inhibitors. After identification of key responses in the transcriptome we could compare and characterise their association to developmental, toxicokinetic, and toxicodynamic processes using the parameter estimates for affected gene clusters. Furthermore, we discuss an association of toxicogenomic effects with measured internal concentrations. **Conclusions** The design and analysis pipeline described here could serve as a blueprint for creating comparable toxicogenomic fingerprints of chemicals. It integrates, aggregates, and models time and concentration-resolved toxicogenomic data.

Key words: risk assessment; environmental monitoring; Adverse Outcome Pathway (AOP); mode of action; 'omics time course; dose response; machine-learning; diuron; diclofenac; naproxen

Background

Chemical risk assessment and environmental monitoring are challenged to find ways of accounting for a large variety and quantity of chemicals [1], which are developed, used, and discharged by modern societies [2], and to which wildlife [3] and humans [4] are exposed during the course of their lifetimes. Hence, methodological innovation for an improved and

Compiled on: March 11, 2019.
Draft manuscript prepared by the author.

Key Points

i. Comparability between toxicogenomic experiments can be improved with the help of:

- the zebrafish toxicogenomic universe – a self-organising map (SOM) of various toxicogenomic datasets providing a common reference frame for biological interpretation, and
- a regression model allowing quantitative characterisation of biological responses and inference on a concentration and time scale.

ii. In a case study the dynamics of key responses (related to e.g. developmental delay, stress response and cyclooxygenase (COX) inhibition) could be identified and discriminated.

more comprehensive characterisation of human and environmental exposures to chemicals [5] and their related effects [6] is sought.

Offering comprehensive response detection, toxicogenomic methods are suggested for an improved assessment of chemical related hazards [7] or environmental health [8]. Because chemicals induce characteristic transcriptome changes (toxicogenomic fingerprints) in tissues [9] and whole organisms [10], 'omics approaches provide novel possibilities for exposure and effect diagnosis for ill-characterised chemicals and environmental samples [11, 12] and may extend the prediction of toxicity on the basis of mechanistic information [13]. In this regard, comparison or connection of different experiments becomes crucial for the interpretation of toxicogenomic observations [14].

When comparing gene expression profiles, the sheer amount of signals in an 'omics dataset poses a quest for comparison and extraction of relevant patterns [15]. The application of self-organising maps (SOMs), a machine learning method developed by Kohonen [16], has been shown to be valuable for the comparison of transcriptome profiles of different tissues [15] and cancer subtypes [17]. Here, we aimed at improving comparability of toxicogenomic fingerprints with the help of a SOM. This is not yet an established approach in toxicogenomics.

Furthermore, comparability between toxicogenomic datasets is typically limited due to substantial differences in study designs, e.g. with respect to selected exposure time and concentration [18]. In their pioneering studies investigating toxicogenomic fingerprints Hamadeh et al. [9] and Yang et al. [10] showed that responses vary with exposure time and concentration. This implies that comparative interpretations of toxicogenomic fingerprints undergo a risk of deriving ambiguous conclusions, when neglecting the concentration and time dependence of the reported responses. Additionally, this severely limits the scope for interpretation or prediction of effects for untested exposures in risk assessment or monitoring efforts. Therefore, comparability would require means for extrapolation. Studies that have analysed time or concentration-resolved toxicogenomic fingerprints applied correlation networks [e.g. 19], unsupervised clustering [e.g. 20], or a set of different regression models [e.g. 21, 22] to describe the responses. However, an integration of concentration *and* time dependence in one model has not yet been achieved for toxicogenomic responses. Therefore, in this study we strived for establishing a regression model capturing time and concentration dependence of toxicogenomic responses.

Taken together we aimed to *integrate*, *aggregate* and *model* dynamic toxicogenomic responses in order to obtain aggregated compound fingerprints, which can be extrapolated on the scale of exposure duration and concentration, and which are comparable between different compounds and studies.

To address the raised issues, we developed an analysis pipeline combining the algorithm of self-organising maps (SOMs) with a concentration and time-dependent response-model (CTR-model). With the SOM we integrated previously published toxicogenomic data to a reference frame which we called *toxicogenomic universe* and aggregated toxicogenomic fingerprints from single compounds to this reference frame to foster comparison between the fingerprints. A regression model was built to derive quantitative parameters for comparing response dynamics and extending the scope for inference.

In order to demonstrate the added value of the suggested approach, we performed an experimental case study and applied the pipeline on microarray data of the zebrafish embryo (*Danio rerio*) after exposure to three selected environmentally relevant contaminants. The experimental design covered six different exposure durations and five increasing compound concentrations. The three compounds were diclofenac and naproxen, two pharmaceuticals known to inhibit the enzyme cyclooxygenase (COX) in humans, and diuron, a herbicide known to target the arylhydrocarbon receptor (AHR) pathway in mammalian cells [23].

Besides gene-expression we also measured the internal concentrations of all three compounds after the exposure. Together with parameter estimates from the CTR-model this allowed to separate toxicodynamic from toxicokinetic responses. Finally, we discuss the suggested analysis pipeline for achieved progress in inferential statements on compounds effects, and outline further uses in the field of toxicogenomics.

Data description

In this study we integrated transcriptome data of the zebrafish embryo (ZFE) from public databases with transcriptome data from our own exposure experiments to infer a universal SOM. Based on this, we performed a case study further investigating the time and concentration-resolved toxicogenomic fingerprints from our exposure experiments. In this section we briefly describe the dataset used for generating the SOM and explain the experimental design and selection of model compounds for the case study.

Dataset for generating the toxicogenomic universe

For the generation of a toxicogenomic universe for the ZFE we used the toxicogenomic fingerprints of the model substances measured in this study in combination with previously published toxicogenomic fingerprints in the ZFE. Data were selected, downloaded and processed from Gene Expression Omnibus (GEO) and ArrayExpress in a semi-automatic workflow, which can be accessed via protocols.io (doi.org/10.17504/protocols.io.s24eggw). A summary of datasets included

in our study is provided in Table S1. The included microarray platforms were annotated to the most recent zebrafish genome (Genome Reference Consortium Zebrafish Build 11), and Ensembl gene annotation (Ensembl database release 93 [24]).

Case study

For our case study, investigating time and concentration-dependent toxicogenomic responses in the ZFE, we selected three environmentally relevant model compounds, namely diuron, diclofenac, and naproxen:

Diclofenac (CAS RN: 15307-79-6) is used as a pharmaceutical substance, often applied as a pain killer and to reduce inflammation. It belongs to the group of non-steroidal anti-inflammatory drugs (NSAIDs) and is a known inhibitor of both variants of the COX enyzme. COX produces prostaglandins, which act as inflammatory signalling molecules [reviewed in 25]. By inhibiting COX an inflammatory response is repressed. As environmental toxicant, diclofenac gained attention due to its toxicity in vultures, which has led to a significant decline in the vulture population in Pakistan [26]. Furthermore, it was identified as a priority pollutant in aquatic environments [e.g. 27]. Several toxicological studies were performed using aquatic organisms [reviewed in 28]. In fish, adverse effects of diclofenac on gill, liver, kidney and the gastrointestinal tract, as well as reduced egg growth and delay in hatching have been reported. Diclofenac has also been associated with drug-induced liver toxicity in response to the formation of reactive metabolites, mitochondrial dysfunction and impairment of ATP synthesis [29, 30].

Naproxen (CAS RN: 26159-34-2), like diclofenac, is widely applied as a COX inhibitor of the NSAID group. It has been detected in surface waters [31, 32, 27] and it was shown to lead to histopathological liver damage and pericardial edema in ZFEs [33].

The third compound used in this study was diuron (CAS RN: 330-54-1), a herbicide listed as a priority substance to be monitored under the European Water Framework Directive [34]. In plants, it is known to specifically inhibit the electron transfer from photosystem II. In mammalian cells, it was found to bind to the AHR [23]. In the ZFE, diuron has been reported to provoke sublethal effects on heartbeat and spontaneous movements [35]. We thus expected diuron to act differently compared to diclofenac and naproxen.

Experimental design

Exposure settings for our transcriptome measurements were designed to meet several requirements: We intended to follow compound specific toxicodynamic processes but also account for differences in toxicokinetics. Most importantly, results were meant to be comparable among the different compounds.

The exposure for a standard ZFE toxicity test starts immediately after fertilisation [36]. However, as we expect many unspecific effects when disturbing the first hours of development, we opted for an exposure period between 24 and 96 hours post fertilization (hpf). Time points of RNA-extraction during the exposure were 3, 6, 12, 24, 48, and 72 hours post exposure (hpe). The exposure concentrations were phenotypically anchored to the lethal concentration (LC) at 96 hpf/72 hpe. The LC_{25}, modelled from experimental observations (see supplementary methods) served as highest and the $LC_{0.5}$ as lowest exposure concentration with 6 equal dilution steps in between, with dilution steps 1, 2, 4, and 6 chosen for exposure (see Equation 1, Equation 2, Figure S1). The selected concentrations for transcriptome experiments can be found in the supplementary methods file.

$$\text{Dilution factor (df)} = \sqrt[6]{\frac{LC_{25}}{LC_{0.5}}} \quad (1)$$

$$\text{Exposure concentrations} = \frac{LC_{25}}{df^x}; x = 0, 1, 2, 4, 6 \quad (2)$$

Data and code availability

The microarray data of this study have been deposited in NCBI's Gene Expression Omnibus [37] and are accessible through GEO Series accession number GSE109496 (https://www.ncbi.nlm.nih.gov/geo/query/acc.cgi?acc=GSE109496). The functions used for analyses and figures have been compiled in the R-package *toxprofileR*, which is available via (https://git.ufz.de/itox/toxprofileR/).

Data analyses and Results

Our analysis aimed at obtaining aggregated dynamic toxicogenomic fingerprints from the measured transcriptome data. The key parts of the analysis workflow (depicted in Figure 1) are

- *integration* of previously published and new toxicogenomic datasets using a self-organising map (SOM) into the *Zebrafish Embryo Toxicogenomic Universe* (ZTU) (Figure 2);
- *aggregation* of compound toxicogenomic fingerprints by projection onto the ZTU (Figure 3);
- *modelling* of time and concentration-resolved responses using a regression model (Figure 4);
- *exploration* of the analysis results with the help of an interactive toxicogenomic fingerprint browser (Figure 5).

In the following, we will describe the analysis steps and the respective results in more detail. Subsequently, we report the results of a case study in which we applied the workflow.

Integration: The *Zebrafish Embryo Toxicogenomic Universe* (**ZTU**)

A compiled dataset of published toxicogenomic data was combined with data from our three single compound exposures to infer the *Zebrafish Embryo Toxicogenomic Universe* (ZTU) based on all currently retrievable toxicogenomic zebrafish embryo (ZFE) microarray data. All datasets were normalised against the respective control of the same experiment. The resulting dataset containing log_2(fold-changes) (*log*FCs) from 342 different treatments and for 29046 unique genes was used to infer a self-organising map (SOM) (Figure 2). This method organises genes into groups of co-regulated or co-expressed transcripts. Those groups are arranged on a two-dimensional grid in a way that similar behaving (i.e., co-expressed) groups end up in the same regions. Each coordinate on the map gets assigned a distinct group of genes. As the ZTU is derived from toxicogenomic data we call this coordinate *toxnode* with reference to the term *node* used in general network terminology (equivalent to the term *metagene* in Wirth et al. [15]). The outcome of this step is a 60×60 grid of 3600 toxnodes. Each gene present in our dataset is permanently assigned to one toxnode, while each node contains genes which behave similarly across all exposure conditions. The number of genes per toxnode ranges from one up to 54 genes with an average of 7 genes per node (Figure 2A).

To obtain an overview over the ZTU we grouped the 3600 toxnodes into 118 clusters (which we determined to be among the optimal cluster sizes, see supplmentary methods) with the help of k-means clustering. To enable easy description of the

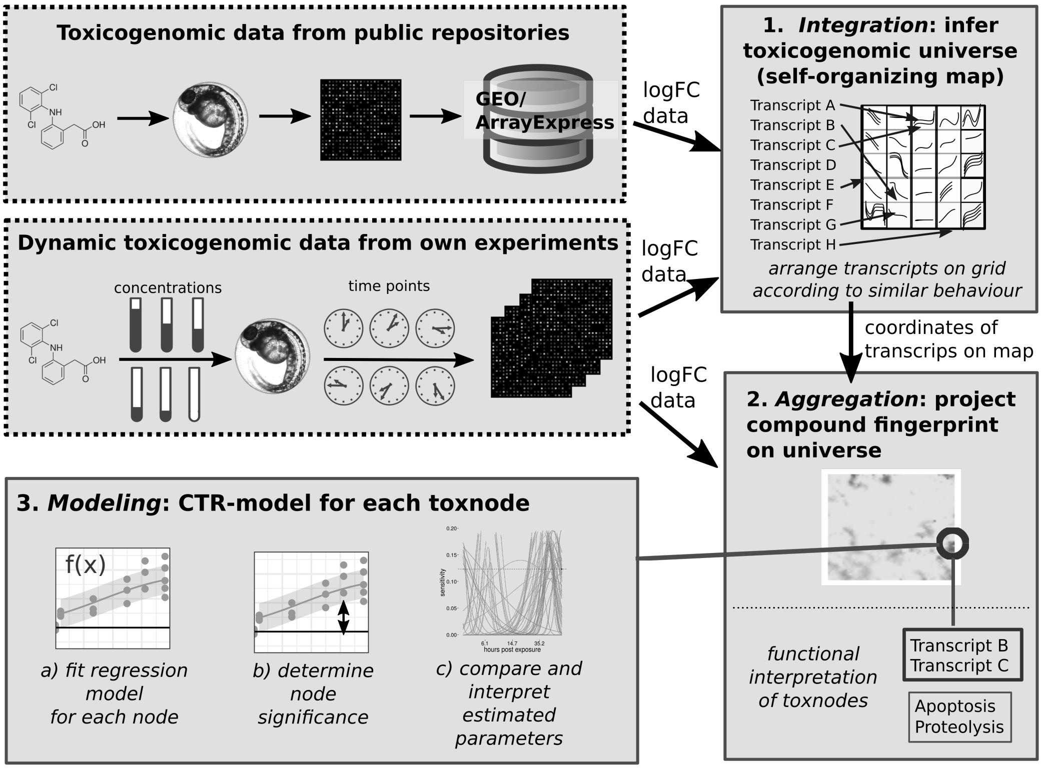

Figure 1. Flowchart of analysis pipeline to obtain dynamic toxicogenomic fingerprints.

clusters a random name was assigned to each cluster of nodes. The clustering is visualized in Figure 2B, and summarized in Table S2. The resulting clusters contained between 3 and 93 toxnodes with an average of 30.

The data integration and clustering with the help of the SOM and subsequent k-means clustering may help in biological interpretation of toxicogenomic responses in the ZFE. We performed an over-representation analysis for functional annotation terms from the databases ZFIN [38], InterPro [39], Reactome [40], and Gene Ontology (GO) [41, 42]. Biological annotations of at least one of the four databases are significantly enriched for 100 of 118 clusters in the ZTU (Table S3-S5). The clusters with the highest proportion of genes assigned to a common function in the four databases are cluster *Trae* containing mainly a set of different crystallin genes (InterPro domain: *Beta/gamma crystallin* in 37 of 52 genes, enriched with an adjusted p-value of 3×10^{-89}; ZFIN: *solid lens vesicle*, 15 of 52 genes, adj. p-value 5×10^{-11}), the cluster *Dakota* containing different vitellogenin genes (GO: *lipid transporter activity*, 5 of 10 genes, adj. p-value: 7×10^{-11}; ZFIN: *unfertilized egg*, 3 of 10 genes, adj. p-value: 6×10^{-6}; interpro: *vitellogenin, open beta-sheet*, 6 of 10 genes, adj. p-value: 9×10^{-19}), and cluster *Vincent*

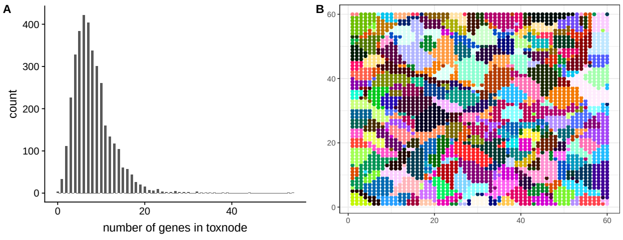

Figure 2. Response *integration*: The Zebrafish Embryo Toxicogenomic Universe (ZTU) comprising of 3600 toxnodes. A: Number of genes per toxnode. B: 118 clusters of toxnodes, each color represents a distinct cluster. For cluster assignments also compare Table S2.

containing genes enriched for the upstream regulator RUNX1 as well as for oxygen transport (Reactome: *RUNX1 regulates transcription of genes involved in differentiation of keratinocytes*, 9 of 42 genes, adj. p-value: 2×10^{-21}; GO: *oxygen transport*, 5 of 42 genes, adj. p-value: 9×10^{-11}).

Examples of further functional enrichments for toxnode clusters, which are affected and explained in detail later on in our case study, are cluster *John*, containing a set of genes expressed in the pancreas (ZFIN: *pancreas*, 14 of 87 genes, adj. p-value: 1×10^{-10}), cluster *Karan*, containing genes associated with cell death (GO: *regulation of cell death*, 10 of 56 genes, adj. p-value: 7×10^{-5}), cluster *Pauline*, containing genes associated with phase II biotransformation (Reactome: *Danio rerio: phase II - conjugation of compounds*, 10 of 56 genes, adj. p-value: 3×10^{-12}), and cluster *Taamira*, containing genes associated with the arachidonic acid pathway (GO: *arachidonic acid metabolic process*, 3 of 23 genes, adj. p-value: 2×10^{-5}).

Aggregation: Compound fingerprints projected on the ZTU

The ZTU retrieved in the previous step can be used as a universal coordinate system to project any exposure specific fingerprint. In Figure 3 this is exemplary shown for the treatment with naproxen (see Figures S2 and S3 for diuron and diclofenac treatments, respectively). Here, the average *log*FCs of each toxnode for the different exposure settings are shown. This allows us to obtain an impression of the response to exposure against a compound for the defined conditions.

We observe that the fingerprints show regulation in both directions (up and down-regulation). It also becomes obvious that fingerprints differ between exposure compound, duration, and concentration, but also show some commonalities. Unlike it might have been expected, diclofenac and naproxen – both known to inhibit the same enzyme – show distinctly different patterns in their toxicogenomic fingerprints.

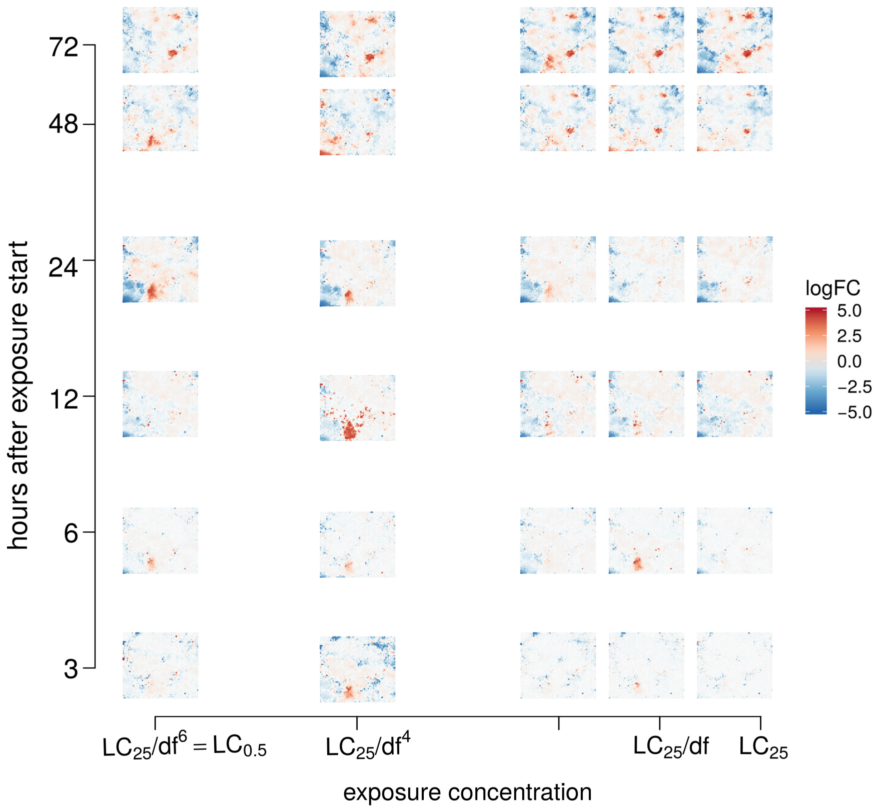

Figure 3. Response *aggregation*: Toxicodynamic fingerprint of naproxen projecting the responses of 30,000 transcripts on 3,600 nodes in the toxicogenomic universe. Shown is a grid of the mean *log*FC fingerprints for each sampled time point/exposure concentration. df: dilution factor (1.15 for naproxen) LC_{25}: exposure concentration at which 25% of embryos show lethal effects after 72 hours of exposure (309 µmol/L for naproxen)

These observations can already give some insight about the toxicogenomic responses, yet they only allow for anecdotal interpretations. For a more generalisable exploration, a modelling approach was deemed helpful and followed in the next step.

Modelling: Regression models for time and concentration dependent toxicogenomic responses

The analysis up to this step allows the consideration of findings for each exposure setting in isolation. In order to arrive at a more general and transferable response characterisation, which allows more than qualitative extrapolation and comparison between substances, we strived for a quantitative description of the measured transcriptional changes. Therefore, we implemented a regression model, capturing the toxicogenomic responses over concentration and time for different substances.

The concentration and time-dependent response-model (CTR-model) describes concentration dependence in a *mono*phasic and time dependence in a *bi*phasic manner. Therefore, we call it *mobi*-CTR-model, here. It is based on the Hill equation, a 3-parameter non-linear model, originally describing the binding of oxygen to haemoglobin as dependent on oxygen saturation [43]. Due to its flexibility on the one hand and physiological meaningfulness on the other hand, it was later on used in many applications [reviewed in 44] and also proposed for pharmacological dose response modeling [45]. One representation of the Hill-equation is provided in Equation 3. It is defined by the parameters $logFC_{max}$, *slope*, and X_{50}. The parameter $logFC_{max}$ is the maximum logarithmic fold change observed for the respective transcript or toxnode, the *slope* defines the steepness of the curve and X_{50} defines the concentration, for which the response (i.e., *log*FC) reaches half-maximum.

The progression of the response over time can be captured by a time dependent description of the parameter X_{50} in Equation 3. Empirically, we discovered that the dynamics of the reciprocal of X_{50} is in many cases accurately captured by the logarithmic Gaussian function (Equation 4). We call the reciprocal of X_{50} *sensitivity*, since a large value indicates a sensitive response. When inserting Equation 4 into Equation 3, we obtain a complete regression model describing the time *and* concentration dependent *log*FC after compound exposure (Equation 5):

$$logFC(c) = \frac{logFC_{max}}{1 + e^{-slope*(\log(c)-\log(X_{50}))}} \tag{3}$$

$$sensitivity(t) = \frac{1}{X_{50}(t)} = S_{max} * e^{-0.5*\left(\frac{log(t)-log(t_{max})}{S_{dur}}\right)^2} \tag{4}$$

$$\begin{aligned} logFC(c,t) = \\ & logFC_{max} / \Big[1 + exp\,(-slope * (\log(c) - \\ & \log(1/(S_{max} * exp(-0.5 * (log(t) - \\ & log(t_{max}))/S_{dur})^2)))\Big)\Big] + \epsilon, \\ & \epsilon \sim \mathcal{N}(0,\ \sigma^2), \end{aligned} \tag{5}$$

where $logFC_{max}$ corresponds to the maximum fold change of the respective node across all conditions, S_{max} is the maximum sensitivity ($1/EC_{50}$) of the gene, t_{max} is the point in time with maximum sensitivity, and S_{dur} represents a measure of duration of the sensitivity interval.

An exemplary model fit is shown in Figure 4 for toxnode #1119. This node is sensitive in response to the exposure against all three substances. The different dynamics are reflected in the parameter estimates. For example, t_{max} is substantially smaller for diuron (8.8 hpe), than for diclofenac (41.3

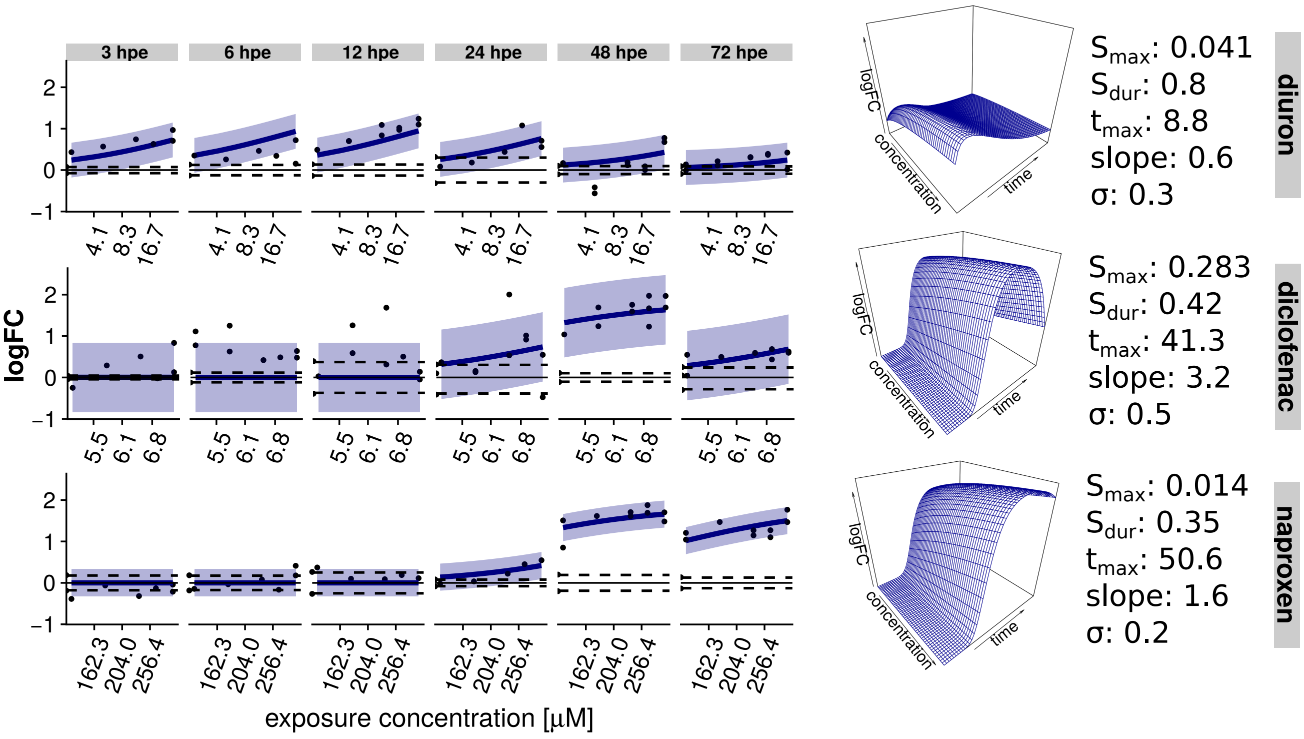

Figure 4. Response *modelling*: Model fit for response of toxnode #1119 - containing the gene for nfe2l2b - towards model compound exposure. The regression model allows a three dimensional interpolation of time and concentration dependence. Shaded areas indicate a 95% confidence interval; dashed lines indicate 2.5%/97.5% quantile of the respective controls.

hpe) and naproxen (50.6 hpe), reflecting an earlier response for the former. The smaller values of S_{dur} for diclofenac (0.42) and naproxen (0.35) in comparison to diuron (0.8) indicate a shorter time frame of sensitivity for this toxnode regarding both of the COX inhibitors. The values of S_{max} indicate that the toxnode responds much more sensitive to diclofenac exposure in comparison with the other two compounds.

The *mobi*-CTR-model was fitted to the measured responses (i.e, *logFC*s) of each toxnode arriving at a quantitative aggregation of time and concentration-dependent toxicogenomic responses. In contrast to Figures 3, S2, and S3, we can now aggregate the response information to one fingerprint, and accordingly one plot, per substance, by projecting the estimates for a parameter on the ZTU. These aggregated fingerprints then allow a systematic analysis as we will demonstrate in the case study below.

Quality of data description by fitted model. The model fitting algorithm converged for all nodes and thus provided viable parameter estimates. There is no trivial measure for goodness of fit for non-linear models (such as R^2 for linear models, compare [46]). Therefore, the quality of data description by the model was determined using the small sample Akaike information criterion (AIC_c)-weight compared to a null model. In the vast majority of cases the regression models are preferred over the null model (Figure S4 A-C). When comparing the regression models to the more flexible spline fit (Figure S4 D-F) which is assumed to offer the optimal data description here, there are (as could be expected) many toxnodes, for which the spline provides better data description. However, for roughly 20% of the nodes the *mobi*-CTR-models are even preferred over a spline fit, thus indicating a good description of the data by the model fit. In contrast to the CTR-model the spline fit does not offer much scope for inference. The major advantage of the CTR-model is that the parameters can be interpreted in a biological context.

Selection of significantly affected toxnodes. Typically, we only expect a small fraction of the toxnodes to show a statistically significant response after exposure to a specific compound. To judge whether a node shows a significant regulation in our exposure scenario, we compared the 95% confidence interval for the regression model fits with the 2.5% and 97.5% quantiles of control measurements. We selected those nodes with a sum of differences between these curves above or below zero (see Figure S5A for visualization). This resulted in a total number of 432 significantly affected nodes with 60 nodes for diuron, 73 nodes for diclofenac, and 353 nodes for naproxen exposure, meeting this criterion (Tables S7-S9). Eight nodes are regulated in both diuron and diclofenac exposures (one in different directions), 22 nodes in diuron and naproxen (6 in different directions), and 18 nodes in diclofenac and naproxen exposures (3 in different directions), three nodes are regulated in exposures of all three compounds (Figure S5B).

Exploration: Toxicogenomic fingerprint browser

To ease the exploration of the toxicogenomic fingerprints in the context of the ZTU, we created an online fingerprint browser (https://webapp.ufz.de/itox/tfpbrowser). A screenshot of the browser is shown in Figure 5. The browser allows visualising fingerprints of different exposure conditions and provides details about toxnode responses and genes that are assigned to the respective nodes. It is possible to select different substances and exposure conditions (Figure 5A) or to search for genes in the universe (Figure 5B). After treatment selection the respective fingerprint is shown (Figure 5C). When selecting a toxnode on the fingerprint or searching for a specific gene name, the CTR-model fit is shown (Figure 5D). Furthermore, the member genes and some functional annotation are displayed (Figure 5E).

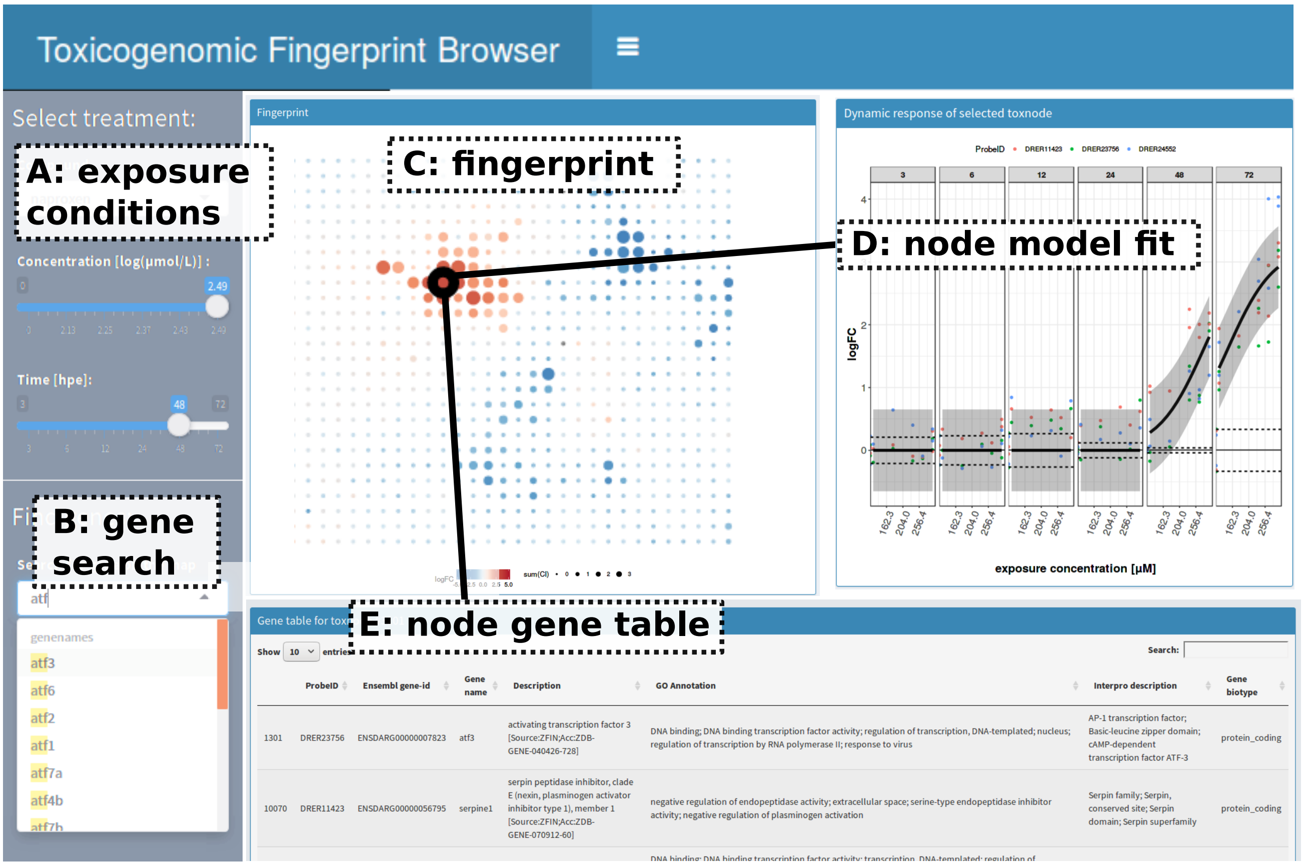

Figure 5. Response *exploration*: Screenshot of interactive toxicogenomic fingerprint browser (available via https://webapp.ufz.de/itox/tfpbrowser). This online tool allows to access visualizations of toxicogenomic fingerprints, model fits and detailed investigation of single toxnodes.

Case study: Investigation of toxicogenomic fingerprints of three model compounds

In order to demonstrate the added value of our approach, we conducted a case study and applied the described pipeline on toxicogenomic data of the three model compounds diuron, diclofenac, and naproxen. By applying a combination of fingerprint projection on the ZTU and regression modelling, as it was described above, we received quantitative, dynamic toxicogenomic fingerprints of the three compounds. This is exemplarily shown in Figure 6 where generalized representations of the three dynamic fingerprints are visualised by a projection of parameter estimates for t_{max} on the ZTU. In the figure each significantly affected toxnode is coloured according to the estimated t_{max}. The size of each dot indicates the extent of regulation for the measured conditions. Furthermore, some node clusters are highlighted which we will discuss below.

The figure shows that naproxen exposure affects considerably more toxnodes in the ZTU than exposures against diuron or diclofenac. We can identify commonly and differentially affected toxnodes and clusters on the map, e.g. cluster *John*, comprising many toxnodes affected late during exposures against diuron and naproxen, cluster *Trae* affected early by diuron and diclofenac, or cluster *Roman* only induced by diuron, as we will discuss in more detail below.

Generally, there are two kinds of information we can deduce from the dynamic toxicogenomic fingerprints: First, the clustering of genes into the same toxnode or region of the ZTU may indicate a common upstream regulator or common cellular process which the genes are involved in. Thus, the member genes of toxnodes affected by a compound exposure may provide qualitative functional information about the response. Second, the estimated model parameters provide quantitative information about the dynamics (i.e., t_{max}, S_{dur}) and the concentration dependence (i.e. S_{max}, *slope*) of the effects. Additionally, the ratio of $\frac{min(EC_{50})_{morphological}}{min(EC_{50})_{toxnode}}$ ($ratio_{m/t}$) indicates the concentration range that lies between effects on toxnode regulation and morphological effects observable under the microscope.

In this regard, we inspected the toxicogenomic fingerprints retrieved by the pipeline (lists of significantly affected toxnodes can be found in supplementary tables S7–S9). We explored the affected nodes and their model parameters in the fingerprint of diuron and compared this to the fingerprints of diclofenac and naproxen. In doing so, we specifically focused on effects which could be linked to an exposure against COX inhibitors.

Diuron

We found 60 toxnodes in 35 clusters to be significantly affected in the fingerprint of diuron (Figure 6A, Table S7). Here we will focus on the effects in cluster *Roman*, *Nikkii*, *Trae*, and *John*.

Roman. The most prominently affected node in the fingerprint of diuron is #818 in cluster *Roman*. It comprises of genes for phase I biotransformation enzymes of the cytochrome P450 family CYP1 (cyp1a, cyp1c1, cyp1c2). The t_{max} was fitted to 18.4 hpe. With a S_{dur} of 0.8 it belongs to the toxnodes with the most sustained response after diuron exposure. Diuron is known to bind to the AHR in mammalian cells [23] which is an upstream regulator of CYP1 genes. The strong induction of these genes in the toxicogenomic fingerprint indicates a persistent interaction of diuron with the AHR in ZFE.

Nikkii. Additionally, we observed toxnodes #2223 and #2283 to be up-regulated in the fingerprint. These nodes are assigned

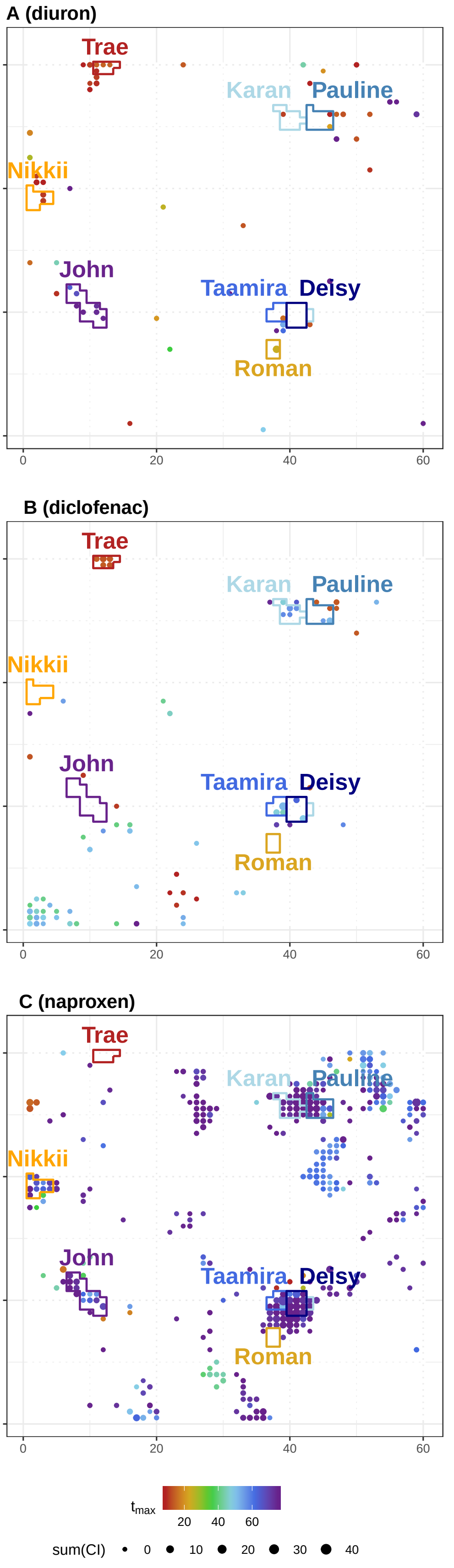

Figure 6. *Case study:* Fitted parameter values for time point of maximum sensitivity (t_{max}) of all significant toxnodes projected on toxicogenomic universe. Dotsize represents significant effect level (sumCI, compare Figure S5A). Selected clusters are highlighted in the plots and are discussed in the text.

to cluster *Nikkii*, which is enriched for genes involved in the phototransduction pathway (Table S5). Both toxnodes were induced early (t_{max} 3.3 and 3.8 hpe, respectively) with a high sensitivity compared to other nodes ($ratio_{m/t}$ > 10). Similar parameter estimates were found for the three significantly induced nodes of cluster *Robert* (#3431, #3550, #3549) and *Tiana* (#3310, #3370, #3371), which are enriched for retinal photoreceptor layer and the neuronal system (t_{max} between 1.5 and 3.8 hpe, $ratio_{m/t}$ > 10). This early induction of the phototransduction pathway was not observed with the other two compounds and may be connected to an observed increase of locomotor response after diuron exposure [35, 47]. Some of these nodes as well as some other nodes of the clusters *Nikki* and *Robert* were found to be down-regulated with naproxen, though significantly later and less sensitive with a t_{max} between 57 and 75 hpe and a $ratio_{m/t}$ < 1.5 (see also Figure 6).

Trae. Toxnodes #3551, #3552, and #3553 belong to cluster *Trae* and were down-regulated early after diuron exposure (t_{max} = 8 hpe for all three nodes). The same was observed with diclofenac where six nodes of cluster *Trae* were down-regulated with similar values for t_{max} between 6.1 and 7.3 hpe (Figure 6). This cluster did not appear to be affected in the naproxen fingerprint. As already mentioned above, cluster *Trae* is highly enriched for crystallin genes (Table S4).

John. In contrast to this early regulation, we found toxnodes #1151, #1328, #1149, #1211, #1092, and #1387, all belonging to cluster *John*, as down-regulated with diuron exposure with a t_{max} between 62 and 74 hpe indicating a late response. This cluster is significantly enriched for pancreatic enzymes (Table S6). We also observed these, as well as six additional nodes of the same cluster, to be down-regulated in the fingerprint of naproxen with similar values for t_{max} estimated between 53 and 75 hpe (Figure 6).

Diclofenac and Naproxen

By comparing the fingerprints of the three compounds we found 15 toxnodes to be significantly up- or down-regulated in the same direction in response to the two known COX inhibitors, naproxen and diclofenac, without showing a significant regulation in response to diuron. A selection of estimated CTR-model parameters for these nodes are summarized in Table 1. Similar as for diuron, the most prominently affected nodes after diclofenac and naproxen exposure contain genes for biotransformation enzymes (compare Tables S8-S9). Yet, the specific enzymes were in part different from the ones up-regulated with diuron exposure and were mainly located in the clusters *Taamira* and *Pauline* in the ZTU as opposed to cluster *Roman* under diuron exposure.

Taamira. Cluster *Taamira* is, among others, enriched for genes annotated with phase I functionalization and arachidonic acid metabolism (Table S5). It contains two toxnodes, which are specifically induced by the COX inhibitors: Node #1179 is most prominently affected with both diclofenac and naproxen exposures and contains the gene cyp2k18, coding for a phase I metabolic enzyme of the cytochrome P450 family which was shown to be induced by different known hepatotoxicants in Poon et al. [48]; the neighbouring node #1118 contains a gene coding for cyp2c9 (a paralogous enzyme of cyp2k18) and the genes abcc2 and abcb5 coding for ABC transporter proteins. The membrane transporter Abcc2 is known to eliminate especially phase II biotransformation products including conjugated drugs from the cells [49]. The affected nodes in cluster *Taamira* have a fitted t_{max} between 44 and 54 hpe for diclofenac, which is around 25 hours later compared to the regulation of biotransformation induced by diuron. For naproxen the fitted

t_{max} of theses nodes is falling between 69 and 75 hpe, and therefore another 15 hours later compared to diclofenac (compare Table 1). The sensitivity of the nodes is comparably high for both compounds, but higher for naproxen with a $ratio_{l/t}$ of 6.1 compared to 2.3 for diclofenac.

Pauline. In cluster *Pauline*, which is enriched, among others, for phase II biotransformation, glutathione transferase activity, and detoxification of reactive oxygen species (Table S3, S5), toxnode #2985 is specifically up-regulated by diclofenac and naproxen. It contains genes coding for metabolic enzymes like carbonyl reductase 1-like enzyme (cbr1l), dehydrogenase/reductase (SDR family member 13 like 1, dhrs13l1), persulfide dioxygenase (ethe1), microsomal glutathione S-transferase (mgst3b), and a sulfotransferase (sult6b1). With a t_{max} of 52.6 hpe (diclofenac) and 53.7 hpe (naproxen) it belongs to the earliest regulated toxnodes appearing in the fingerprints of both diclofenac and naproxen. Other toxnodes of this cluster, such as #3045, are as well induced with both COX inhibitors, even though not significantly with diclofenac, and contain genes such as peroxiredoxin (prdx1) or glutathione S-transferases (gsta2, gstp1) and reductases (gsr). Most of these enzymes belong to the group of oxidoreductases and the results of the over-representation analyses indicate their involvement in response to oxidative stress.

Deisy. Two of the most prominently induced toxnodes in diclofenac and naproxen fingerprints belong to cluster *Deisy*: Toxnode #1062 is up-regulated with a t_{max} of 46 hpe with diclofenac and 72 hpe with naproxen. It contains the genes for the two hormones leptin alpha (lepa) and parathyroid hormone 1a (pth1a); toxnode #1241 contains different variants of the heat-shock protein hsp70 and is induced with t_{max} values of 62 and 64 hpe for diclofenac and naproxen, respectively. The induction of hsp70 by NSAIDs has been shown before [e.g. 50]. Also, a change in leptin levels after diclofenac exposure has been reported [29]. The induction of leptin might be linked to the arachidonic acid pathway [51], which is disturbed by the inhibition of COX [52]. Additionally, leptin levels are related to the state of energy metabolism [53], which indicates that the COX inhibitors might induce a change in energy metabolism in the ZFE. This is further corroborated by the induction of toxnode #1120 in the same cluster (not significant with diclofenac), containing the genes for cocaine- and amphetamine-regulated transcript 3 (cart3) and apoptosis facilitator Bcl-2-like protein 14 (CABZ01020840.1). Up-regulation of cart3 has been associated with anorexigenic effects in response to stress in adult zebrafish [54], while the BCL2 family of proteins is known to

Table 1. Parameter estimates (mobi-CTR model) for toxnodes specifically affected by diclofenac and naproxen in our experiment. $ratio_{l/t}$: $min(LC_{50})/min(X_{50})_{toxnode}$; $ratio_{m/t}$: $min(EC_{50})_{morphological}/min(X_{50})_{toxnode}$. Positive sum(CI) indicate up-regulation, negative indicate down-regulation of respective toxnodes. $logFC_{max}$ represents maximum *logFC* per node across all treatments in our experiments.

tn#	genes	cluster	$logFC_{max}$	t_{max}		S_{max}		$sum(CI)$		$ratio_{l/t}$		$ratio_{m/t}$	
				DIC	*NPX*	*DIC*	*NPX*	*DIC*	*NPX*	*DIC*	*NPX*	*DIC*	*NPX*
1118	abcc2, CYP2C9, abcb5	Taamira	2.2	44.1	75.0	0.283	0.017	2.84	7.80	2.3	6.1	2.0	3.0
1179	cyp2k18	Taamira	4.8	48.9	69.0	0.283	0.017	12.02	34.12	2.3	6.1	2.0	3.0
2985	cbr1l, dhrs13l1, ethe1, mgst3b, sult6b1	Pauline	2.1	52.6	53.7	0.094	0.017	0.01	2.31	0.8	6.1	0.7	3.0
1062	lepa, pth1a	Deisy	3.6	46.1	72.0	0.229	0.017	5.38	8.60	1.9	6.1	1.6	3.0
1241	hsp70l, hsp70.1, hsp70.2	Deisy	3.3	61.6	64.0	0.149	0.017	2.35	10.39	1.2	6.1	1.1	3.0
3039	dusp1, cyr61, gadd45ba, sik1, nfkbiaa	Karan	1.7	55.2	72.9	0.146	0.005	0.07	0.11	1.2	1.8	1.0	0.9
3040	btg2, zgc:162730, tcima, ier2b, egr2a, jdp2b	Karan	2.1	55.6	75.0	0.148	0.007	0.09	1.33	1.2	2.5	1.1	1.2
3100	socs3a, fosab	Karan	3.3	53.8	73.0	0.180	0.006	2.59	2.96	1.5	2.1	1.3	1.1
3101	serpine1, atf3, junba	Karan	3.2	54.0	75.0	0.137	0.007	0.53	7.96	1.1	2.5	1.0	1.2
1000	isg15	Farajallah	5.1	72.4	75.0	0.113	0.008	0.08	17.17	0.9	2.9	0.8	1.4
3161	socs3b, timp2b, CR855311.1, clu	Farajallah	2.6	63.9	74.7	0.141	0.007	0.06	4.89	1.1	2.5	1.0	1.2
17	RF00020	Tashina	2.5	75.0	62.6	0.140	0.007	0.72	5.47	1.1	2.5	1.0	1.2
2041	si:ch211-125e6.5, si:ch211-125e6.14, zgc:172053	Vincent	1.5	75.0	74.3	0.164	0.004	0.02	0.61	1.3	1.4	1.2	0.7
3157	igfbp1b	Daniel	2.7	73.6	75.0	0.136	0.009	0.08	5.83	1.1	3.2	1.0	1.6
3173	pdzd3a	Talon	−2.0	49.3	74.0	0.131	0.007	−0.05	−4.33	1.1	2.5	0.9	1.2

regulate stress induced apoptosis [55].

Karan/Farajallah. Further COX inhibitor-specific toxnodes (Table 1) belong, among others, to cluster *Karan* (#3100, #3101, #3039, #3040) and the cluster *Farajallah* (#3161, #1000). Cluster *Karan* is significantly enriched for MAP-kinase phosphatase activity (Tables S3, S4, S5; e.g. gene dusp1), transcription factors of the AP1 family and the toll-like receptor cascade (Tables S4, S5; e.g. genes fosab, jdp2d, atf3, junab, nfkbiaa), as well as the regulation of cell death and cell cycle (Table S3; e.g. genes cyr61, gadd45ba, junba). Additionally, it is enriched for an inflammatory response to biotic stimulus which is also true for cluster *Farajallah* which is, in line with that, also enriched for the complement cascade (Tables S3, S5). Furthermore, toxnodes containing genes known to be involved in immune response are part of cluster *Karan* but only found to be affected by naproxen. For example, this is toxnode #3041, containing COX2b (here ptgs2b), serum/glucocorticoid regulated kinase 1 (sgk1), CCAAT enhancer binding protein beta (cebpb), which all have shown to be involved in inflammation [56, 57].

Regulation of Karan/Farajallah by Deisy. Several genes and pathways observed to be up-regulated in cluster *Karan* have been reported to be regulated by leptin, which is induced comparatively early within the cluster *Deisy* (see above). For example, leptin is known to induce mitogen-activated protein kinase (MAPK) cascades as well as the JAK/STAT signaling pathway [reviewed in 58] and stimulate the expression of socs3 as feedback regulator as well as timp1 [reviewied in 59]. The stimulation of c-fos genes by leptin mediated via the STAT3 pathway was also reported before [60]. Furthermore, leptin and parathyroid hormone (PTH) were shown to regulate COX-2 mRNA expression [61, 62, 63]. Therefore, we hypothesise that leptin is one of the key regulators of the responses in cluster *Karan* which are induced later than *Deisy* with a t_{max} of 54-55 hpe with diclofenac and 73-75 hpe with naproxen.

The t_{max} values of the cluster *Farajallah* (64-72 hpe with diclofenac, 75 hpe with naproxen) indicate an even more downstream response induced after the induction of Karan. With a $ratio_{l/t}$ between 0.9 and 1.5 (diclofenac) and 1.8 and 2.9 (naproxen) the sensitivity of responses in *Karan* and *Farajallah* is lower in comparison to the nodes in *Taamira* or *Deisy*.

Common responses in all three compound fingerprints

With our analysis pipeline we identified three toxnodes as significantly induced with all three compounds, namely node #2986 (cluster *Pauline*), containing one gene coding for the phase II enzyme ugt1a, its potential regulator nfe2l2b in node #1119 (cluster *Taamira*), and node #998 (cluster *Farajallah*) containing an orthologue gene for cathepsin S. The early induction of nfe2l2b (*alias* nrf2b), a master regulator of oxidative stress [64, 65], and the induction of its potential target gene ugt1a [66], hint to the induction of the oxidative stress response cascade in the ZFE. Furthermore, the dynamics of this induction (for an example see Figure 4) is different between the compounds and seem to follow the chemical uptake dynamics of the compounds which will be discussed in more detail below. Further nodes of these three clusters were induced with all three compounds.

Global sensitivity dynamics

We observed above, that the sensitivity dynamics of selected toxnodes substantially differs between the investigated compounds. We analysed, whether there are global differences in sensitivity dynamics between the compounds by examining the distributions of parameter estimates for t_{max} and S_{max}.

Diuron exhibits the most distinct early regulation of the three investigated compounds. This is also reflected in the distribution of estimates for t_{max} (Figure 7A) showing two peaks at around 1.5 hpe and 75 hpe for all significantly affected toxnodes. The S_{max} of some affected nodes is calculated to be up to two orders of magnitude higher than the sensitivity for lethality (no morphological sublethal effects were observed for diuron). The median ratio between morphological and toxicogenomic sensitivity of all significantly affected toxnodes is 2.8.

The response towards diclofenac exposure shows two peaks at 7 hpe and 50 hpe (Figure 7B), which is 4 and 47 hours later, respectively, in comparison to the first peak of responses with diuron exposure. There are only few toxnodes with a t_{max} later than 60 hpe. For diclofenac the ratio between morphological sensitivity and toxnode S_{max} is not larger than 2.3 for any of the affected toxnodes. The median ratio is 1.4.

Naproxen clearly shows the latest response of the three substances, reflected by the distribution of t_{max} showing a small peak at around 60 hpe and a high peak at the latest time point at 75 hpe. The $ratio_{m/t}$ shows a maximum of 6 for some of the toxnodes. The median ratio is 3.2.

Internal concentrations

The observed dynamics of transcriptional responses seem to be partially linked with the temporal pattern of internal chemical concentrations. Figure 7D-F depicts the increase of internal chemical concentration for the three compounds. It demonstrates different kinetics of chemical uptake in the ZFE. Whereas the highest internal dose of diuron is reached before 20 hpe (Figure 7D), this peak is observed between 40 and 60 hpe with diclofenac (Figure 7E) and not before the last observed time point at 72 hpe with naproxen (Figure 7F). This matches with the observation that most affected toxnodes show a t_{max} smaller than 20 hpe for diuron (Figure 7A), between 40 and 60 hpe for diclofenac (Figure 7B), and not before the last time point at 75 hpe for naproxen (Figure 7C).

By comparing the CTR-model fit depicted in Figure 4 for the oxidative stress response marker nfe2l2b with the internal concentrations dynamics, we see a correlation of these results with t_{max} values of 8 hpe for diuron, 41 hpe for diclofenac, and 51 hpe for naproxen (compare Figure 4 and Figure 7D-F).

Overall, this shows that toxicogenomic sensitivity can be strongly influenced by toxicokinetic properties of the respective substances. The comparison of parameter values of the CTR-model such as the t_{max} with additional information such as the internal dose dynamics also led to the identification of stage specific, toxicokinetic-independent responses such as the down-regulation of the clusters *Trae* and *John*.

Discussion

The objective of this study was to improve comparability of toxicogenomic datasets by advancing the scope of inference for toxicogenomic fingerprints. Therefore, we developed and tested an experimental and data analysis pipeline for creating dynamic toxicogenomic fingerprints of chemicals. Here, we discuss the suggested approach as to the aspired comparability and scope for inference, as well as the added value with regard to the elucidation of molecular, cellular, or physiological effects of chemicals.

Approach: Map and model toxicogenomic responses

Our pipeline tackles two major challenges with regard to toxicogenomic analyses: first, to integrate and aggregate toxicogenomic datasets; and second, to integrate time and concentration dependence.

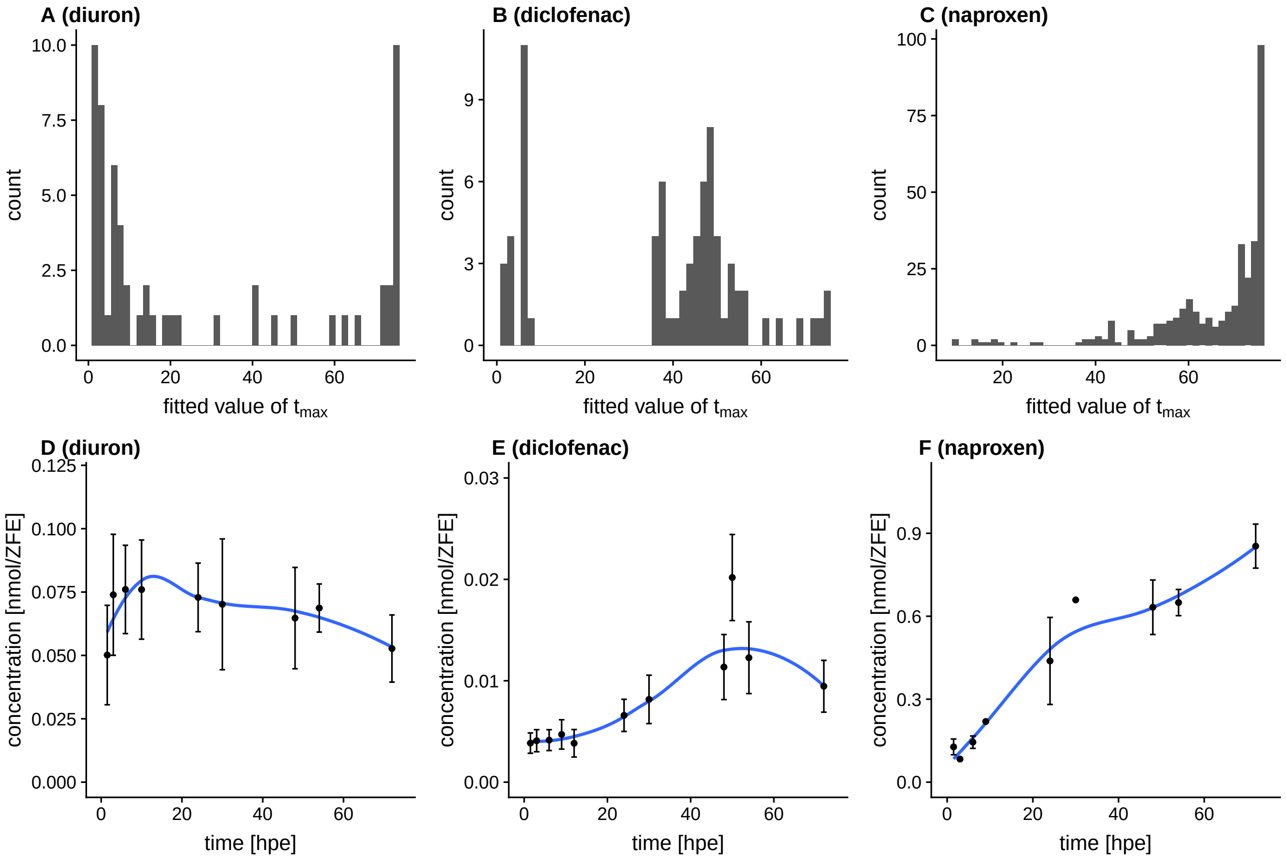

Figure 7. Sensitivity dynamics and toxicokinetics; A-C: Distribution of fitted parameter values among significantly regulated nodes for t_{max}; D-F measured internal concentration (mean ± standard error)

Integration and aggregation of toxicogenomic responses
A wealth of gene expression signatures is publicly available (e.g. Gene Expression Omnibus containing roughly 2.7 million samples in October 2018) and efforts are increasing for gaining new insights by integrating large numbers of datasets. For example, the connectivity map approach, establishing links between similar gene expression profiles [14] was applied by Wang et al. [67] to explore similarities between toxicogenomic fingerprints in fish. This method can serve as a relatively simple and easily scalable approach to find similar profiles in a toxicogenomic database. However, the pairwise linking is based on qualitative lists of differentially expressed genes only, no explicit inclusion of time and concentration resolution is considered, and the outcome does not immediately aid in aggregating the responses of a single experiment, i.e. aggregation takes place on the level of meta-data, only.

Another approach for integrating toxicogenomic datasets is the inference of gene networks based on correlation or mutual information. These networks can be analysed for modularity and interrogated for specific changes in nodes or edges after chemical perturbation. This was applied by Perkins et al. [13], for example, to reverse engineer adverse outcome pathways (AOPs) from mutual information networks, or by Woo et al. [68] to identify drug targets by analysing altered network interactions. However, comparability and integration of dependent variables such as time or concentration are still limited with these approaches.

A specific, more rigid network form is the self-organising map (SOM). The algorithm was developed by Kohonen [16]. It was first applied to gene expression data by Törönen et al. [69] and Tamayo et al. [70] and has been further developed and tested for aggregating tissue expression profiles by Wirth et al. [15, 71]. While SOMs have mainly been used to aggregate information from single datasets, we applied SOMs to *integrate* an extensive compilation of toxicogenomic datasets, and use the resulting grid afterwards to *aggregate* the fingerprints of single substances. A SOM shows limitations in capturing complex interactions compared to the more 'flexible' networks in the aforementioned studies (i.e. connections between nodes are only formed between direct neighbours on the map). Therefore some relevant interactions between transcriptomic units might get lost with the SOM. Additionally, co-expression of genes is not necessarily consistent across different perturbations [68]. This would not be captured in the dynamic toxicogenomic fingerprints as shown here. However, SOMs have the advantage of enabling visualisation, interpretation, and comparing treatments on a whole transcriptome scale, as well as reducing the number of analysed entities. With the help of the SOM we can retrieve information about co-expression of genes from publicly available toxicogenomic datasets and include this information into our analysis irrespective of differing experimental designs and occurrence of missing data In our approach we could reduce the analysis space from ~30,000 transcripts to 3,600 toxnodes or 118 clusters. Altogether, this allowed us to comprehensively analyse commonly and differentially regulated toxnodes and clusters in the Zebrafish Embryo Toxicogenomic Universe (ZTU) and to derive functional hypotheses from this (see below). A common functionality of genes within some of the identified clusters was confirmed in our overrepresentation analysis (e.g. the clustering of crystallin or pancreas genes). Such a 'recovery of the known' demonstrates the viability of the approach [14].

Furthermore, the clustering of genes into toxnodes allows the combination of data aggregation with modelling of time

and concentration dependence. The aggregation also improves model quality, since data from several genes can be used for estimating one parameter set (see below). The projection of toxicogenomic responses on a universal map fosters comparison between different profiles, which can be compared visually and quantitatively with the help of model parameters, as discussed below. While in our study the analysis focused on microarray data, the approach is not limited to microarray data and could also be applied on RNA sequencing data. This is also demonstrated in Figure S8B showing RNAseq data projected on the toxicogenomic universe. The supplementary methods file (supplementary_methods.html) demonstrates how to use our supplied R-package *toxprofileR* and the toxicogenomic universe to infer toxicogenomic fingerprints of additional compounds.

Modelling of time and concentration dependence

A couple of studies have been published which investigated toxicogenomic fingerprints at varying exposure settings. Among those, only few studies investigated the dynamics of responses. One example is the study by Alexeyenko et al. [19], who studied effect propagation at several time points after dioxin exposure in ZFEs and found changes in gene-gene interactions between different points in time. The application of several concentrations in toxicogenomic experiments is reported more frequently, e.g. by Driessen et al. [72], Chen et al. [61], or Sonnack et al. [73]. Yet, all of the mentioned studies analysed the different exposure conditions in isolation, thus only allowing qualitative statements about time or concentration dependent changes. In a study by Hermsen et al. [20] genes were clustered according to their concentration dependence across seven different concentrations. Although this approach acknowledged concentration as a continuous variable, the description of concentration dependence remains observational in this study, also.

This was advanced in studies by Thomas et al. [21] and Smetanová et al. [22] which showed that concentration dependence of toxicogenomic responses can be captured by using regression modelling on significant responses applying a selection of different models. The individual description of responses using different regression models, however, limited the comparability.

In contrast to these studies by Thomas et al. [21] and Smetanová et al. [22], we fitted a uniform concentration and time-dependent response-model (CTR-model) to all toxnodes in the toxicogenomic universe in order to derive node-specific parameter estimates. Subsequently, we used the confidence intervals of the fitted model for detection of statistical significance. This approach might have the limitation, that we cannot capture each response as accurately as the aforementioned studies. For example, toxnodes showing a biphasic response on the concentration scale would not be accurately captured. Yet, our approach implicates advancements regarding comparability in several ways: First, there is a unique set of model parameters, whose estimates can be compared across different transcripts, toxnodes, or compound exposures. Second, model parameters are fitted independently from the statistical significance of changes between treatment and control, i.e. no preselection of genes is necessary. Therefore, model parameters can be compared between significantly and non-significantly regulated toxnodes. Moreover, significance of regulation is rather determined by a mechanistically motivated model (i.e. an adaptation of the Hill-model), adding biological significance to a mere statistical treatment vs. control comparison. Additionally, the aggregation of the responses of several transcripts into toxnodes enhanced the robustness of the model fits and implies that varying responses of single transcripts have a reduced impact on the aggregated outcome of a toxnode in the toxicogenomic fingerprint.

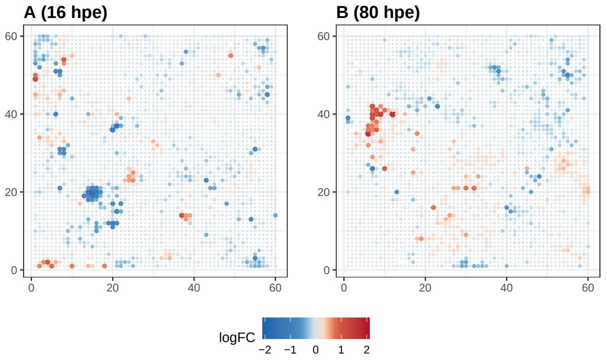

Figure 8. Predicted toxicogenomic fingerprints for diuron at the environmentally relevant concentration 0.86 µmol/L. A: 16 hpe; B: 80 hpe. Both, concentration and time point are outside the measured range in our case study.

Furthermore, the time dependence of responses is not considered in previous approaches [21, 22]. The *mobi*-CTR-model adapted in our study captures both concentration *and* time dependence of the responses. In this way, the model allows inference of a three-dimensional, time and concentration-resolved response pattern as it is shown in Figure 4.

The application of the *mobi*-CTR-model in the analysis of toxicogenomic responses has implications for the experimental design. Since response information is not based on pairwise comparisons (treatment vs. control) but on time and concentration-resolved response characteristics, we can use a dense sampling design with few replicates only. This goes in line with a study by Sefer et al. [74] which showed that in high-throughput testing dense sampling should be preferred over replicate sampling. Our findings reveal that the measurement of only one time point or a single concentration would not have been sufficient to identify toxnodes as being commonly regulated by all three substances as it is illustrated in Figure 4.

As it is not conceivable that concentrations, selected and measured in experiments, will ever cover the whole range of concentrations relevant for estimating or interpreting environmental effects, measures for extrapolation become relevant. In this regard, the CTR-model allows building hypotheses and predictions about toxicogenomic effects at conditions not measured. For example, with the help of the model we can predict for each toxnode in the ZTU which response we would expect for an environmentally relevant concentration of 0.86 µmol/L of diuron [concentration measured in 75]. This is falling outside the concentration range measured in our study. In Figure 8 the expected *logFC*s for 16 and 80 hpe are projected on the ZTU. Without investigating the profiles in detail, we can observe that the predictions show distinctly different profiles. A prominently down-regulated cluster can be seen in the early time point (blue spot bottom left) and a prominently up-regulated cluster in the late time point (red spot upper left). This demonstrates once more that one substance may induce distinctly different effect profiles depending on time and concentration. Furthermore, if such profiles were indeed measured in an environmental sample, we would only be able to link these profiles to a diuron exposure with dynamic and concentration-dependent information as supplied by the CTR-model.

Certainly, regression models as they are used in our study also have clear limitations. So far the regression model describes responses for a specific exposure setting. Other experimental settings may require refining the model. When e.g. temporal variation in exposure regimes become relevant for extrapolation, the development of models explicitly integrating kinetic and dynamic processes would be desirable [e.g. 76, 77]. Data demands regarding time and dose-resolved observations have restricted their application so far. We see our

approach with regard to experimental design and analytical pipeline, therefore, as a step on the avenue to advanced dynamic modelling, which could further progress towards mechanistic models.

Finally, the improved comparability, which was discussed here, eases a consistent interpretation in a toxicological context. As it was exemplarily demonstrated for the response of toxnode #1119 in Figure 4, toxnode responses cannot only be described qualitatively ('is regulated significantly after exposure to substance X') but also quantitatively with the help of estimated parameter values like S_{max} or t_{max} describing the concentration or time related response. This allows, for the first time, to link toxicogenomic processes with toxicokinetic measurements (as it was shown in Figure 7), thus separating toxicokinetic from toxicodynamic processes. In our case study we found a significant impact of toxicokinetic properties of the substances on the dynamics of various toxicogenomic responses which is discussed below in more detail. It also helped to identify those responses which seem to be independent on toxicokinetics and rather related to the developmental stage (e.g. regulation of clusters *Trae* or *John*).

Case study: Compound effects in the zebrafish toxicogenomic universe

We found that different compounds with a known identical molecular target still show individual toxicogenomic response patterns on the ZFE transcriptome. This goes in line with earlier studies investigating toxicogenomic fingerprints e.g. in rat liver tissues [9] or zebrafish embryos [10], which reported toxicogenomic profiles to turn out specific for compound, concentration and exposure duration. Advancing from the compound and treatment-specific response barcodes for a few selected transcripts [10], we obtained concentration and time-resolved, transcriptome-wide toxicogenomic fingerprints for each compound, which can be comprehensively compared between substances.

The clustering of genes into common toxnodes and clusters of the ZTU as illustrated above is indicative for jointly regulated processes, functionally related proteins, or tissue/cell type specificity of genes in the ZFE. Simultaneously, we obtain information about the compound-specific characteristics of the response from the estimated model parameters. In order to illustrate the added value of the approach, we will discuss some of the insights we gained about unspecific toxicogenomic responses in the ZFE and common key responses induced by the two COX inhibitors.

Unspecific key responses

By combining information on model parameter values with functional information on nodes and clusters we can distinguish between specific and unspecific effects. For example, we identified cluster *Trae* and cluster *John* to be related to lens and pancreas development, respectively. While *Trae* is regulated early with diclofenac and diuron, *John* is regulated late with diuron and naproxen. The t_{max} for the significantly down-regulated nodes in these clusters were identical for the respective two compounds and independent from the different chemical uptake kinetics. This is in contrast to responses, for which t_{max} was in line with internal substance kinetics (e.g. the induction of the transcription factor nfe2l2b). The t_{max} values of cluster *Trae* were estimated at around 6-8 hpe, which equals 30-32 hpf. Cluster *Trae* contains more than 20 genes coding for crystallin protein subunits which are important for lens development in ZFE, which happens between 16 hpf and 96 hpf [78, 79]. Those transcripts were found to be commonly down-regulated in response to various chemical exposures in earlier

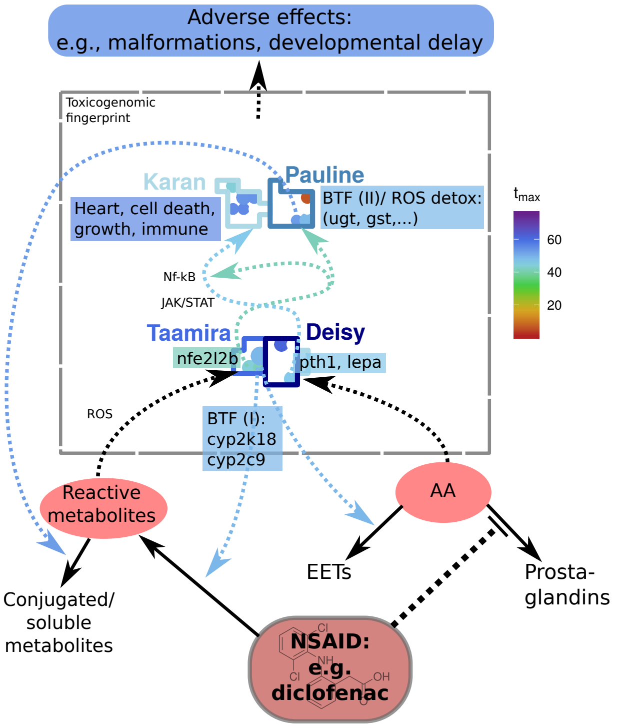

Figure 9. Hypothesised key responses of COX inhibitors in the toxicogenomic fingerprint of diclofenac. Putative causal connections between responses are indicated by dashed arrows; solid arrows indicate transformation reactions arraycolor represents estimated tmax for diclofenac; btf: biotransformation (phase I/II); ROS detox: detoxification of reactive oxygen species; AA: arachidonic acid. EETs: Epoxyeicosatrienoic acids; NSAID: non-steroidal antiinflammatory drug; Note: the plot shows a simplified/idealized version of the toxicogenomic fingerprint, the indentified responses, and their connections.

studies [80, 18], as well. Cluster *John* is down-regulated at 72 hpe and contains genes coding for different proteolytic enzymes of the pancreas. Indeed, the pancreas development in ZFE starts rather late between 36 and 72 hpf [81], and a disruption of pancreas development in ZFE by exposures against different chemicals has also been reported in several studies before [e.g. 64, 82, 83].

By comparing our raw data of treatments and controls for these two clusters we found that assuming a delay or inhibition of development due to the chemical treatment may explain the down-regulation of transcriptional activity (Figure S7). The combination of comparing our model parameters between the different exposures, the enrichment of genes in the ZTU, as well as the confirmation by available reports on down-regulation due to chemical exposures let us conclude that these effects on pancreas and lens development are potentially independent of the compound, indicating a developmental delay due to general stress. Whether these delays manifest in adverse outcome is potentially rather a matter of exposure concentration and time than of the specific mode of action of a compound.

Key responses induced by COX inhibitors

Two of the investigated model compounds, diclofenac and naproxen, are known to affect the same molecular target, namely COX. In order to identify key responses of this compound group we studied the patterns commonly evoked by these compounds. We discuss the identified key responses in the context of known molecular effects of COX inhibitors. Our findings and hypothesised molecular key responses are summarised and illustrated in Figure 9.

Metabolism and biotransformation. A predominant response in the fingerprints of diclofenac and naproxen is the up-regulation of metabolic enzymes mainly in cluster *Taamira*. For example, we observed an early and strong induction of cyp2c9 and cyp2k18 with a t_{max} of 48 hpe for diclofenac and 69 hpe for naproxen. We assume that these enzymes are involved in the phase I biotransformation (BTF(I)) of diclofenac and naproxen. This may lead to an increased production of reactive metabolites leading to liver injury [29]. Additionally, we hypothesise that an inhibition of COX, which transforms arachidonic acid (AA) to prostaglandins [52], leads to an accumulation of AA. This might induce COX-independent branches of the AA pathway, e.g. the production of epoxyeicosatrienoic acids (EETs) [84]. This reaction is known to be catalysed, among others, by enzymes of the cyp2c family [85]. Therefore, cyp2c9 and cyp2k18 may be involved in the biotransformation of the COX inhibitors diclofenac and naproxen themselves, but also in the production of EETs from AA being accumulated due to COX inhibition.

Besides induction of BTF(I), glucuronosyltransferases ugt1 and ugt5 were induced earlier than many other significant responses in our study. These enzymes were shown to be involved in the phase II biotransformation (BTF(II)) of diclofenac in humans [86] and fish [87]. Additionally, they can lead to accumulation of acyl glucuronides and subsequent formation of protein adducts [88]. Together with an increase in reactive metabolites from BTF(I) this is discussed to be a molecular key event in the adverse outcome pathway (AOP) of COX inhibitors, leading to mitochondrial dysfunction, impairment of ATP synthesis, apoptosis and tissue damage, and eventually causing liver and cardiovascular diseases [reviewed in 29]. The occurrence of the initial key events of biotransformation is indicated by the affected genes and toxnodes in our study. The observation of biotransformation being among the first observed key responses in time might indicate that later observed responses are mediated by metabolites instead of the parent compounds or by secondary effects such as an accumulation of AA.

Oxidative stress response. Next to biotransformation, glucuronosyltransferases are also associated with an oxidative stress response which is another key response in the fingerprints of the COX inhibitors in our study. Genes associated with oxidative stress are mainly found in cluster *Pauline*, and are potentially induced by the transcription factor NRF2, whose zebrafish orthologue nfe2l2b is induced early within cluster *Taamira* [64, 65]. AA-induced NRF2-dependent gene transcription has been reported in brain cells [89] whereas the induction of NRF2 has been shown to prevent toxicity of AA in human liver cells [90]. Next to ugt1, several genes for oxidoreductases and glutathione metabolism were specifically induced with the COX inhibitors in cluster *Pauline*.

We confirmed the assignment of cluster *Pauline* to oxidative stress response with a dataset from the background data of the ZTU. Paraquat, a herbicide known to induce oxidative stress [91], was investigated using the ZFE in Driessen et al. [92]. We plotted the transcriptome response on the ZTU (Figure S8A) and found cluster *Pauline* induced as well as one node in cluster *Bradley*. Cluster *Bradley* is enriched for respiratory electron transport (Table S5). The induction of these clusters with paraquat confirms the biological meaningfulness of the ZTU. However, it only provides a snapshot without evidence on potentially related nodes and clusters or any possibility of extrapolation. Nevertheless, it gives a strong indication of a key response of oxidative stress induced by diclofenac and naproxen. Indeed, oxidative stress has been discussed as a side effect of diclofenac and other NSAIDs before [93, 94].

Induction of regulatory hormones. Another key response in the COX inhibitor fingerprints was the up-regulation of the regulatory hormones leptin alpha and PTH1a in cluster *Deisy*. The induction of leptin together with cart3 in the same cluster might be due to a stress related change in energy metabolism [54] or due to AA accumulation [51]. Leptin induces a MAPK pathway (JAK/STAT) which is an activator of NF-kB [58, 95]. Furthermore, PTH was shown to regulate the ligand of NF-kB in mammalian osteocytes [96, 97]. Indeed, we identified the genes and nodes of the subsequently induced clusters *Karan* and *Farajallah* to be targets of these pathways [98] (also see results part and Figure 9). The interaction of COX inhibitors with these pathways as cyclooxygenase-independent effects were reported by Tegeder et al. [99]. Indeed, little is known about the molecular interactions of AA, leptin, and PTH. A role of AA in leptin signalling and hepatic energy metabolism has been described before [100]. Furthermore, it has been reported that leptin can induce the secretion of parathyroid hormone [e.g. 101]. However, a strong co-expression of the two hormones as observed in our study has not been reported before and indicates a joint action in response to chemicals or stress in ZFE.

Interestingly, when projecting the toxicogenomic fingerprint of BDE-47, measured by Xu et al. [102], on the ZTU, we see a similar fingerprint as with naproxen and diclofenac (Figure S8B). We find, among others, toxnode #1062 containing lepa and pth1a, as well as some other toxnodes of the clusters *Taamira* and *Karan* up-regulated with BDE-47. A study by Kodavanti and Derr-Yellin [103] showed that polybrominated diphenyl ethers as well as polychlorinated biphenyls cause a release of arachidonic acid in rat neurons. This might indicate that the obtained phenotypes, which were similar with BDE-47 exposures [104] to the ones we observed with the two COX inhibitors (Figure S2), showing tail malformations, spinal curvature, small eyes and edema, are related or initially caused by a disturbance of the AA metabolic pathway.

In summary, using a comparative analysis of the knowledge gained from the ZTU combined with compound specific parameter values from the CTR-model, we could identify and characterise key responses to diuron, diclofenac, and naproxen exposures in the ZFE. Furthermore testable hypotheses about the sequence of key responses and their connections to toxicokinetic, toxicodynamic or developmental processes were generated as it is illustrated in Figure 9.

Summary and Implications

With our experimental design and analysis pipeline we derived dynamic toxicogenomic fingerprints. The applied regression model allows inference on the concentration as well as the time scale to conditions not measured. It proves also helpful in separating toxicokinetic from toxicodynamic processes. The *Zebrafish Embryo Toxicogenomic Universe* (ZTU) introduced here allows to aggregate toxicogenomic fingerprints on a map. Taken together, this novel approach facilitates comparison between different fingerprints and different studies as well as between the responses in one fingerprint. We see several implications that may arise from these results:

The toxicogenomic universe as source for biological hypothesis building and gene selection for high-throughput approaches

We demonstrate in this study that the clustering of genes in the toxicogenomic universe can be used to derive biological hypotheses about co-expressed genes. Furthermore, the toxicogenomic universe can be used to support gene selection for reduced transcriptome approaches. There have been efforts to use reduced transcriptome arrays implying much lower costs,

which allows obtaining more extensive data sets. A reduced mouse transcriptome array was recently used to measure perturbation profiles of more than 20,000 substances in different cell lines [105] generally demonstrating the power of high-throughput molecular approaches for large scale assessments. Recently, also a reduced array for the zebrafish transcriptome was suggested [106] including selected genes to represent a range of biological pathways. This selection, however, was not based on zebrafish experimental data, but focused on orthologues of genes known to be important in mammalian toxicology. Therefore, an alternative approach to design a reduced zebrafish array could comprise the selection of a representative gene for each toxnode of the Zebrafish Embryo Toxicogenomic Universe (ZTU) or the selection of genes within a specific region of interest in the ZTU.

The CTR-model to enhance molecular databases

The scope of functional annotation databases could substantially improve with the inclusion of quantitative exposure and effect information, e.g. as derived from concentration response relationships as shown in our study. We demonstrated that it is possible to quantitatively describe a majority of toxicogenomic responses with a universal regression model. This could be used to enhance annotation databases, such as Gene Ontology [GO, 41], Molecular Signatures Database [MSigDB, 107], or Comparative Toxicogenomics Database [CTD, 108], which, so far, focus on qualitative information about responses.

Dynamic toxicogenomic fingerprints for read-across and elucidation of adverse outcome pathway(s)

The dynamic toxicogenomic fingerprints foster read-across approaches between chemicals by providing enhanced comparability. This could improve application in chemical hazard assessment and effect-based environmental monitoring. The inference and comparison of toxicogenomic universes for different species could furthermore aid in cross-species extrapolation.

The fingerprints can also help in elucidating key events of an adverse outcome pathway (AOP). For this we can use previously derived functional knowledge about responding toxnodes or clusters to draw conclusions about the effects of other substances, as exemplarly shown in Figure S8. In this study we have shown the identification and comparative characterization of key responses for two COX inhibitors. We see this as a helpful starting point for informing the development of AOPs. In this regard, our approach might also help in mechanism-based risk assessment.

Molecular mixture toxicology

Finally, mixture assessment relevant in environmental monitoring becomes possible with the help of the suggested CTR-model. In the context of environmental monitoring of substances, toxicogenomic fingerprints of environmental samples should in principle be suitable to be compared with fingerprints of single substances. However, it remains to be clarified if a) individual toxicogenomic fingerprints can be recovered also in a mixture context, and b) how fingerprints of different substances combine in a qualitative and quantitative way. Existing mixture concepts such as concentration addition or independent action could guide the evaluation of mixture responses and the identification of additive or non-additive behaviour [109]. The dynamic fingerprints inferred here lend themselves for such hypothesis-based experimentation.

Methods

We will briefly outline the experimental procedure and analysis steps performed in our study. We additionally prepared an extensive supplementary methods file supplementary_methods.html. This file contains detailed information about the experimental procedure (exposure, RNA extraction, measurement of toxicokinetics) and the data analysis pipeline (data import, quality control, creating the toxicogenomic universe, regression modelling). It also includes the R code used for generating the results shown in this study. A brief summary is given below. The results have (in part) been computed at a High-Performance Computing Cluster at the Helmholtz Centre for Environmental Research.

Exposure of zebrafish embryos to three model compounds

Zebrafish embryos were exposed to diuron, diclofenac sodium salt, and naproxen sodium salt in five different concentrations between $LC_{0.5}$ and LC_{25} from 24 hours post fertilization (hpf). At six time points between 3 and 72 hours post exposure (hpe) RNA was extracted and the transcriptome was measured using Oaklabs Zebrafish XS Microarrays.

Import, quality control and preprocessing of data

The median fluorescence for each array spot was extracted by the Agilent Feature Extraction Software (Version 11.5.1.1). All further analysis was performed in R (version 3.4.3 [110]).

Quality control was performed by checking density distributions and euclidean distance between samples. Similar as recommended by Kauffmann and Huber [111] we checked four quality metrics, which were Kolmogorov-Smirnov test statistics, sum of all expression values of one array, interquartile range (IQR), and euclidean distance. Samples with one of the metrics outside of a range between 25% and 75% quantile $\pm 3 * IQR$ ($1 * IQR$ for euclidean distance) were removed from further analysis. Processed intensity values were normalized using the *cyclic loess* method.

After normalization all data was transformed by log_2. Subsequently, the median expression values of replicate probes were calculated. If replicates of a probe were present on the array, only replicates which had not been flagged for poor quality during the feature extraction process (due to inhomogeneous spots or background) were considered. Laboratory batch effects in the diclofenac experiment were removed using the R-package *sva* [112].

Transcript abundance is changing quite drastically for many transcripts during the course of embryo development, even without exposure to a chemical (compare Figure S9). At this point the effect of the chemical was of main interest. Therefore, the developmental effect on the transcriptome was removed by normalising all transcript level values against the control of the respective time point. This resulted in log_2(fold-change) (*log*FC) data for all experimental conditions.

Infering the toxicogenomic universe

The spline smoothed *log*FC data from our experiments were combined with the *log*FC data from previously published ZFE microarray data. Data from public databases was selected, downloaded and processed in a semi-automatic workflow, which is accessible via protocols.io (dx.doi.org/10.17504/protocols.io.s24eggw). All included microarray platforms were annotated to the most recent zebrafish genome (GRCz11), and

Table 2. Properties of SOM learning

parameter	value
learning rate	0.8 – 0.005
neighborhood radius	40 – (-40)
neighborhood function	gaussian
epochs	1000
distance function	manhattan distance

Ensembl database version 93 [24].

The Grubb's test ([113], implemented in R-package *outliers*) was used iteratively to remove outliers from the group of data points of each probe (points were removed until $p >= 0.001$). This resulted in 0.2%-0.3% of measurements being removed for each substance. Then, a thin plate spline was fitted to the treatment conditions of each probe using the R-package *mgcv* [114] and *log*FC values for each measurement condition were extracted. The R-package *kohonen* [115] was used to train the self-organising map on a 60 × 60 rectangular grid. The initial learning rate was set comparably high in order to make the node codes quickly adjust to the assigned transcript behaviour. The properties of the map and the learning algorithm are summarized in Table 2.

The outcome of this step is one 60 × 60 grid of 3600 toxnodes. Each gene present in our dataset is permanently assigned to one toxnode, while each toxnode contains genes which behave similarly across all exposure conditions. We used the R-package *mclust* [116] to determine an optimal cluster number for sub-grouping (see supplementary methods for more details). The package *randomNames* [117] was used to automatically name the clusters.

For identification of over-represented annotations within the clusters, we used the package *clusterProfiler* [118] together with annotation from the databases ZFIN [38], InterPro [39], Reactome [40], and GO [41, 42]. We applied correction for multiple testing with the Benjamini-Hochberg method and a p-Value cut-off of 0.05.

Parameter estimation of mobi-CTR model

Normalized *log*FC data (not the spline fit) were used as input data for parameter estimation. Measured data from all probes assigned to one node of the SOM were used to estimate one parameter set for each node and substance (i.e., experimental replicates and transcriptional replicates/groups of transcripts were treated as belonging to one distribution here). The Grubb's test ([113], implemented in R-package *outliers*) was used iteratively to remove outliers from the group of data points (points are removed until p>=0.001) in one node. This resulted in removal of 0.1%-0.2% of data points for each substance. The extreme values across all samples and experimental conditions were determined for each node. Then, the dataset for each node was used to estimate parameters for the *mobi*-CTR using the shuffled complex evolution (SCE) algorithm assuming up-regulation. This estimation procedure was repeated 3 times with 3 different random seeds and 10 complexes each. The best model was afterwards selected using AIC_c. The same procedure was repeated assuming down-regulation. The best up-regulation model and the best down-regulation model were again compared using AIC_c and the best fit model subsequently used for a quantitative description of the node. We used the R implementation of the algorithm shuffled complex evolution (SCE, described in [119]) in the package *hydromad* [120]. For a global parameter estimation method like SCE, parameter boundaries should be defined carefully. To limit the fitted parameter values to a range that makes sense in the context of the experiment, boundaries were set as described in the supplementary methods file.

Fingerprint browser

To ease the exploration of the toxicogenomic fingerprints in the context of the toxicogenomic universe, we created an online application https://webapp.ufz.de/itox/tfpbrowser. The app was created using R in combination with the package *shiny* [121].

Supplementary methods

A supplementary methods file was compiled containing extensive documentation about experimental and data analysis workflow including all R code needed to reproduce the results. The file was created with the help of knitR [122].

Acknowledgements

We would like to acknowledge Silke Aulhorn, Bettina Seiwert, and Martin Krauss for their support with chemical analytics, as well as David Leuthold, Janna Kuhlmann, Daniela Taraba, and Susanne Schmidt for their support with the exposure experiments. Additionally, we thank Henry Wirth for the fruitful discussion about self-organizing maps, Stefan Krämer for valuable feedback on the manuscript, and Jana Schor for an introduction into Rmarkdown. We would like to thank the administration and support staff of EVE and WOMBAT, Thomas Schnicke, Ben Langenberg, Christian Krause, Sven Petruschke, Martin Abbrent, Michael Garbe, and Martin Sand, for keeping everything running, supporting us with our scientific computing needs, and setting up the online access of the Fingerprint Browser.

Availability of source code and requirements

Functions used for the analysis were compiled into the R-package *toxprofileR* which is available via a git repository:

- Project name: toxprofileR
- Project home page: https://git.ufz.de/itox/toxprofileR
- Operating system(s): Platform independent
- Programming language: R (>3.4.3)
- License: GNU GPL version 3
- RRID: SCR_017027

Furthermore, we provide an interactive online tool for exploration of the datasets obtained in our study:

- Project name: Toxicogenomic Fingerprint Browser
- Project home page: https://webapp.ufz.de/itox/tfpbrowser/
- Operating system(s): Platform independent
- Programming language: R (>3.4.3)
- License: GNU GPL version 3
- RRID: SCR_017028

Availability of supporting data and materials

The data set supporting the results of this article is available in the Gene Expression Omnibus repository, [GSE109496].

An interactive online tool for exploration of the datasets obtained in our study can be accessed via https://webapp.ufz.de/itox/tfpbrowser/

Additionally, there are three supplementary files available:

- supplementary_methods.html – extensive documentation about experimental and data analysis workflow including all R code needed to reproduce the results. Source files (R markdown) are available in supplementary_methods_source.zip
- supplementary_figures.pdf – all supplementary figures which were referenced in the paper
- supplementary_tables.zip – supplementary tables with cluster information and significant toxnodes for each substance

Declarations

List of abbreviations

AA arachidonic acid
AHR arylhydrocarbon receptor
AIC$_c$ small sample Akaike information criterion
AOP adverse outcome pathway
BTF(I) phase I biotransformation
BTF(II) phase II biotransformation
COX cyclooxygenase
CTD Comparative Toxicogenomics Database
CTR-model concentration and time-dependent response-model
EET epoxyeicosatrienoic acid
GEO Gene Expression Omnibus
GO Gene Ontology
hpe hours post exposure
hpf hours post fertilization
IQR interquartile range
LC lethal concentration
logFC log_2(fold-change)
MAPK mitogen-activated protein kinase
MSigDB Molecular Signatures Database
NSAID non-steroidal anti-inflammatory drug
PTH parathyroid hormone
SCE shuffled complex evolution
SOM self-organising map
ZTU Zebrafish Embryo Toxicogenomic Universe
ZFE zebrafish embryo

Consent for publication

Not applicable

Competing Interests

The authors declare that they have no competing interests.

Funding

This work was supported by the German Federal Environmental Foundation Scholarship Program (DBU, AZ: 20014/ 350) and the European Union 7th Framework Programme project SOLUTIONS (grant agreement no. 603437)

Author's Contributions

- Conceptualization: AS, RA, WB
- Data curation: AS
- Formal analysis: AS
- Funding acquisition: AS, RA, WB
- Investigation: AS, GJ, JKn, JKr, MA, MBB, WB
- Project administration: WB
- Software: AS
- Supervision: GMW, KR, WB
- Visualization: AS
- Writing - original draft: AS, RA, WB
- Writing - review & editing: AS, GJ, GMW, JKn, JKr, KR, MA, MBB, RA, WB

References

1. Birnbaum LS, Burke TA, Jones JJ. Informing 21st-Century Risk Assessments with 21st-Century Science. Environmental Health Perspectives 2016 Apr;124(4):A60–3. https://ehp.niehs.nih.gov/doi/10.1289/ehp.1511135.
2. Hendriks AJ. How To Deal with 100,000+ Substances, Sites, and Species: Overarching Principles in Environmental Risk Assessment. Environmental Science & Technology 2013 Apr;47(8):3546–3547. http://pubs.acs.org/doi/10.1021/es400849q.
3. Miller TH, Bury NR, Owen SF, MacRae JI, Barron LP. A review of the pharmaceutical exposome in aquatic fauna. Environmental Pollution 2018 Aug;239:129–146. https://linkinghub.elsevier.com/retrieve/pii/S0269749117352375.
4. Jiang C, Wang X, Li X, Inlora J, Wang T, Liu Q, et al. Dynamic Human Environmental Exposome Revealed by Longitudinal Personal Monitoring. Cell 2018 Sep;175(1):277–291.e31. https://linkinghub.elsevier.com/retrieve/pii/S0092867418311218.
5. Rappaport SM, Barupal DK, Wishart D, Vineis P, Scalbert A. The Blood Exposome and Its Role in Discovering Causes of Disease. Environmental Health Perspectives 2014 Aug;122(8):769–774. https://ehp.niehs.nih.gov/doi/10.1289/ehp.1308015.
6. Escher BI, Hackermüller J, Polte T, Scholz S, Aigner A, Altenburger R, et al. From the exposome to mechanistic understanding of chemical-induced adverse effects. Environment International 2017 Feb;99:97–106. https://linkinghub.elsevier.com/retrieve/pii/S0160412016309187.
7. European Food Safety Authority, Aguilera J, Aguilera-Gomez M, Barrucci F, Cocconcelli PS, Davies H, et al. EFSA Scientific Colloquium 24 – 'omics in risk assessment: state of the art and next steps. EFSA Supporting Publications 2018 Nov;15(11). http://doi.wiley.com/10.2903/sp.efsa.2018.EN-1512.
8. Bahamonde PA, Feswick A, Isaacs MA, Munkittrick KR, Martyniuk CJ. Defining the role of omics in assessing ecosystem health: Perspectives from the Canadian environmental monitoring program: Omics for ecosystem health. Environmental Toxicology and Chemistry 2016 Jan;35(1):20–35. http://doi.wiley.com/10.1002/etc.3218.
9. Hamadeh HK, Bushel PR, Jayadev S, Martin K, DiSorbo O, Sieber S, et al. Gene Expression Analysis Reveals Chemical Specific Profiles. Toxicological Sciences 2002;67:219–231.
10. Yang L, Kemadjou JR, Zinsmeister C, Bauer M, Legradi J, Müller F, et al. Transcriptional profiling reveals barcode-like toxicogenomic responses in the zebrafish embryo. Genome Biology 2007;8(10):R227. http://genomebiology.biomedcentral.com/articles/10.1186/gb-2007-8-10-r227.
11. Nuwaysir EF, Bittner M, Trent J, Barrett JC, Afshari CA. Microarrays and toxicology: The advent of toxicogenomics. Molecular Carcinogenesis 1999 Mar;24(3):153–159. http://doi.wiley.com/10.1002/%28SICI%291098-2744%28199903%2924%3A3%3C153%3A%3AAID-MC1%3E3.0.CO%3B2-P.
12. Snape JR, Maund SJ, Pickford DB, Hutchinson TH. Ecotoxicogenomics: the challenge of integrating genomics into aquatic and terrestrial ecotoxicology. Aquatic Toxicol-

ogy 2004 Apr;67(2):143–154. http://linkinghub.elsevier.com/retrieve/pii/S0166445X03002510.

13. Perkins EJ, Chipman JK, Edwards S, Habib T, Falciani F, Taylor R, et al. Reverse engineering adverse outcome pathways. Environmental Toxicology and Chemistry 2011 Jan;30(1):22–38. http://doi.wiley.com/10.1002/etc.374.
14. Lamb J, Crawford ED, Peck D, Modell JW, Blat IC, Wrobel MJ, et al. The Connectivity Map: Using Gene-Expression Signatures to Connect Small Molecules, Genes, and Disease. Science 2006 Sep;313(5795):1929–1935. http://science.sciencemag.org/content/313/5795/1929.
15. Wirth H, Löffler M, von Bergen M, Binder H. Expression cartography of human tissues using self organizing maps. BMC Bioinformatics 2011;12(1):306. http://bmcbioinformatics.biomedcentral.com/articles/10.1186/1471-2105-12-306.
16. Kohonen T. Self-organized formation of topologically correct feature maps. Biological Cybernetics 1982;43(1):59–69. http://link.springer.com/10.1007/BF00337288.
17. Hopp L, Lembcke K, Binder H, Wirth H. Portraying the Expression Landscapes of B-CellLymphoma-Intuitive Detection of Outlier Samples and of Molecular Subtypes. Biology 2013 Dec;2(4):1411–1437. http://www.mdpi.com/2079-7737/2/4/1411.
18. Schüttler A, Reiche K, Altenburger R, Busch W. The Transcriptome of the Zebrafish Embryo After Chemical Exposure: A Meta-Analysis. Toxicological Sciences 2017 Jun;157(2):291–304. https://academic.oup.com/toxsci/article-lookup/doi/10.1093/toxsci/kfx045.
19. Alexeyenko A, Wassenberg DM, Lobenhofer EK, Yen J, Linney E, Sonnhammer ELL, et al. Dynamic Zebrafish Interactome Reveals Transcriptional Mechanisms of Dioxin Toxicity. PLOS ONE 2010 May;5(5):e10465. https://journals.plos.org/plosone/article?id=10.1371/journal.pone.0010465.
20. Hermsen SAB, Pronk TE, van den Brandhof EJ, van der Ven LTM, Piersma AH. Concentration-Response Analysis of Differential Gene Expression in the Zebrafish Embryotoxicity Test Following Flusilazole Exposure. Toxicological Sciences 2012 May;127(1):303–312. https://academic.oup.com/toxsci/article-lookup/doi/10.1093/toxsci/kfs092.
21. Thomas RS, Allen BC, Nong A, Yang L, Bermudez E, Clewell HJ, et al. A Method to Integrate Benchmark Dose Estimates with Genomic Data to Assess the Functional Effects of Chemical Exposure. Toxicological Sciences 2007 Jul;98(1):240–248. https://academic.oup.com/toxsci/article-lookup/doi/10.1093/toxsci/kfm092.
22. Smetanová S, Riedl J, Zitzkat D, Altenburger R, Busch W. High-throughput concentration-response analysis for omics datasets: Concentration-response analysis for omics datasets. Environmental Toxicology and Chemistry 2015 Sep;34(9):2167–2180. http://doi.wiley.com/10.1002/etc.3025.
23. Zhao B, Baston DS, Hammock B, Denison MS. Interaction of diuron and related substituted phenylureas with the Ah receptor pathway. Journal of Biochemical and Molecular Toxicology 2006 May;20(3):103–113. http://doi.wiley.com/10.1002/jbt.20126.
24. Zerbino DR, Achuthan P, Akanni W, Amode MR, Barrell D, Bhai J, et al. Ensembl 2018. Nucleic Acids Research 2018 Jan;46(D1):D754–D761. https://academic.oup.com/nar/article/46/D1/D754/4634002.
25. Ricciotti E, FitzGerald GA. Prostaglandins and Inflammation. Arteriosclerosis, Thrombosis, and Vascular Biology 2011 May;31(5):986–1000. https://www.ahajournals.org/doi/10.1161/ATVBAHA.110.207449.
26. Oaks JL, Gilbert M, Virani MZ, Watson RT, Meteyer CU, Rideout BA, et al. Diclofenac residues as the cause of vulture population decline in Pakistan. Nature 2004 Feb;427(6975):630–633. http://www.nature.com/articles/nature02317.
27. Busch W, Schmidt S, Kühne R, Schulze T, Krauss M, Altenburger R. Micropollutants in European rivers: A mode of action survey to support the development of effect-based tools for water monitoring. Environmental Toxicology and Chemistry 2016 Aug;35(8):1887–1899. http://doi.wiley.com/10.1002/etc.3460.
28. Lonappan L, Brar SK, Das RK, Verma M, Surampalli RY. Diclofenac and its transformation products: Environmental occurrence and toxicity - A review. Environment International 2016 Nov;96:127–138. https://linkinghub.elsevier.com/retrieve/pii/S0160412016304159.
29. Lee EH, Oh JH, Selvaraj S, Park SM, Choi MS, Spanel R, et al. Immunogenomics reveal molecular circuits of diclofenac induced liver injury in mice. Oncotarget 2016 Mar;7(12). http://www.oncotarget.com/fulltext/7698.
30. Syed M, Skonberg C, Hansen SH. Mitochondrial toxicity of diclofenac and its metabolites via inhibition of oxidative phosphorylation (ATP synthesis) in rat liver mitochondria: Possible role in drug induced liver injury (DILI). Toxicology in Vitro 2016 Mar;31:93–102. https://linkinghub.elsevier.com/retrieve/pii/S0887233315300217.
31. Tixier C, Singer HP, Oellers S, Müller SR. Occurrence and fate of carbamazepine, clofibric acid, diclofenac, ibuprofen, ketoprofen, and naproxen in surface waters. Environmental Science & Technology 2003 Mar;37(6):1061–1068.
32. Verenitch SS, Lowe CJ, Mazumder A. Determination of acidic drugs and caffeine in municipal wastewaters and receiving waters by gas chromatography–ion trap tandem mass spectrometry. Journal of Chromatography A 2006 May;1116(1-2):193–203. http://linkinghub.elsevier.com/retrieve/pii/S0021967306005218.
33. Li Q, Wang P, Chen L, Gao H, Wu L. Acute toxicity and histopathological effects of naproxen in zebrafish (Danio rerio) early life stages. Environmental Science and Pollution Research 2016 Sep;23(18):18832–18841. http://link.springer.com/10.1007/s11356-016-7092-4.
34. European Parliament, Council of the European Union, DIRECTIVE 2000/60/EC OF THE EUROPEAN PARLIAMENT AND OF THE COUNCIL of 23 October 2000 establishing a framework for Community action in the field of water policy. Official Journal of the European Communities; 2000.
35. Velki M, Di Paolo C, Nelles J, Seiler TB, Hollert H. Diuron and diazinon alter the behavior of zebrafish embryos and larvae in the absence of acute toxicity. Chemosphere 2017 Aug;180:65–76. https://linkinghub.elsevier.com/retrieve/pii/S0045653517305404.
36. OECD. Test No. 236: Fish Embryo Acute Toxicity (FET) Test. OECD Guidelines for the Testing of Chemicals, Section 2, OECD; 2013. https://www.oecd-ilibrary.org/environment/test-no-236-fish-embryo-acute-toxicity-fet-test_9789264203709-en.
37. Edgar R. Gene Expression Omnibus: NCBI gene expression and hybridization array data repository. Nucleic Acids Research 2002 Jan;30(1):207–210. https://academic.oup.com/nar/article-lookup/doi/10.1093/nar/30.1.207.
38. Howe DG, Bradford YM, Conlin T, Eagle AE, Fashena D, Frazer K, et al. ZFIN, the Zebrafish Model Organism Database: increased support for mutants and transgenics. Nucleic Acids Research 2012 Oct;41(D1):D854–D860. http://academic.oup.com/nar/article/41/D1/D854/1074339/ZFIN-the-Zebrafish-Model-Organism-Database.
39. Mitchell AL, Attwood TK, Babbitt PC, Blum M, Bork P, Bridge A, et al. InterPro in 2019: improving coverage, classification and access to protein sequence annotations. Nu-

cleic Acids Research 2018 Nov;https://academic.oup.com/nar/advance-article/doi/10.1093/nar/gky1100/5162469.

40. Fabregat A, Jupe S, Matthews L, Sidiropoulos K, Gillespie M, Garapati P, et al. The Reactome Pathway Knowledgebase. Nucleic Acids Research 2018 Jan;46(D1):D649–D655.

41. Ashburner M, Ball CA, Blake JA, Botstein D, Butler H, Cherry JM, et al. Gene Ontology: tool for the unification of biology. Nature Genetics 2000 May;25(1):25–29. http://www.nature.com/doifinder/10.1038/75556.

42. Gene Ontology Consortium. Expansion of the Gene Ontology knowledgebase and resources. Nucleic Acids Research 2017 Jan;45(D1):D331–D338. https://academic.oup.com/nar/article-lookup/doi/10.1093/nar/gkw1108.

43. Hill AV. The possible effects of the aggregation of the molecules of haemoglobin on its dissociation curves. The Journal of Physiology 1910;40:i–vii.

44. Goutelle S, Maurin M, Rougier F, Barbaut X, Bourguignon L, Ducher M, et al. The Hill equation: a review of its capabilities in pharmacological modelling. Fundamental & Clinical Pharmacology 2008 Dec;22(6):633–648. http://doi.wiley.com/10.1111/j.1472-8206.2008.00633.x.

45. Wagner JG. Kinetics of pharmacologic response I. Proposed relationships between response and drug concentration in the intact animal and man. Journal of Theoretical Biology 1968 Aug;20(2):173–201. http://linkinghub.elsevier.com/retrieve/pii/0022519368901884.

46. Spiess AN, Neumeyer N. An evaluation of R2 as an inadequate measure for nonlinear models in pharmacological and biochemical research: a Monte Carlo approach. BMC Pharmacology 2010;10(1):6. http://bmcpharma.biomedcentral.com/articles/10.1186/1471-2210-10-6.

47. Leuthold D, Klüver N, Altenburger R, Busch W. Can Environmentally Relevant Neuroactive Chemicals Specifically Be Detected with the Locomotor Response Test in Zebrafish Embryos? Environmental Science & Technology 2019 Jan;53(1):482–493. http://pubs.acs.org/doi/10.1021/acs.est.8b04327.

48. Poon KL, Wang X, Lee SGP, Ng AS, Goh WH, Zhao Z, et al. Transgenic Zebrafish Reporter Lines as Alternative *In Vivo* Organ Toxicity Models. Toxicological Sciences 2017 Jan;156(1):133–148. https://academic.oup.com/toxsci/article-lookup/doi/10.1093/toxsci/kfw250.

49. Jedlitschky G, Hoffmann U, Kroemer HK. Structure and function of the MRP2 (ABCC2) protein and its role in drug disposition. Expert Opinion on Drug Metabolism & Toxicology 2006 Jun;2(3):351–366. http://www.tandfonline.com/doi/full/10.1517/17425255.2.3.351.

50. Mortaz E, Redegeld FA, Bloksma N, Dunsmore K, Denenberg A, Wong HR, et al. Induction of HSP70 is dispensable for anti-inflammatory action of heat shock or NSAIDs in mast cells. Experimental Hematology 2006 Apr;34(4):414–423. https://www.exphem.org/article/S0301-472X(06)00003-8/abstract.

51. Fain JN, Leffler CW, Cowan GSM, Buffington C, Pouncey L, Bahouth SW. Stimulation of leptin release by arachidonic acid and prostaglandin E2 in adipose tissue from obese humans. Metabolism 2001 Aug;50(8):921–928. http://linkinghub.elsevier.com/retrieve/pii/S0026049501293111.

52. Zarghi A, Arfaei S. Selective COX-2 Inhibitors: A Review of Their Structure-Activity Relationships. Iranian journal of pharmaceutical research: IJPR 2011;10(4):655–683.

53. Fried SK, Ricci MR, Russell CD, Laferrère B. Regulation of Leptin Production in Humans. The Journal of Nutrition 2000 Dec;130(12):3127S–3131S. https://academic.oup.com/jn/article/130/12/3127S/4686231.

54. Cortés R, Teles M, Oliveira M, Fierro-Castro C, Tort L, Cerdá-Reverter JM. Effects of acute handling stress on short-term central expression of orexigenic/anorexigenic genes in zebrafish. Fish Physiology and Biochemistry 2018 Feb;44(1):257–272. https://doi.org/10.1007/s10695-017-0431-7.

55. Pihán P, Carreras-Sureda A, Hetz C. BCL-2 family: integrating stress responses at the ER to control cell demise. Cell Death and Differentiation 2017 Sep;24(9):1478–1487. http://www.nature.com/doifinder/10.1038/cdd.2017.82.

56. Burgon J, Robertson AL, Sadiku P, Wang X, Hooper-Greenhill E, Prince LR, et al. Serum and Glucocorticoid Regulated Kinase 1 (SGK1) Regulates Neutrophil Clearance During Inflammation Resolution. Journal of immunology (Baltimore, Md : 1950) 2014 Feb;192(4):1796–1805. https://www.ncbi.nlm.nih.gov/pmc/articles/PMC3921102/.

57. Rahman SM, Janssen RC, Choudhury M, Baquero KC, Aikens RM, Houssaye BAdl, et al. CCAAT/Enhancer-binding Protein beta (C/EBP beta) Expression Regulates Dietary-induced Inflammation in Macrophages and Adipose Tissue in Mice. Journal of Biological Chemistry 2012 Oct;287(41):34349–34360. http://www.jbc.org/content/287/41/34349.

58. Procaccini C, Lourenco EV, Matarese G, La Cava A. Leptin signaling: A key pathway in immune responses. Current Signal Transduction Therapy 2009 Jan;4(1):22–30.

59. Nanjappa V, Raju R, Babylakshmi Muthusamy, Jyoti Sharma, Joji Kurian Thomas, Pachakkil A Haridas Nidhina, et al. A Comprehensive Curated Reaction Map of Leptin Signaling Pathway. Journal of Proteomics & Bioinformatics 2011;04(09):181–189. https://www.omicsonline.org/a-comprehensive-curated-reaction-map-of-leptin-signaling-pathway-jp[illegible]1000188.php?aid=1924.

60. Kaszubska W, Falls HD, Schaefer VG, Haasch D, Frost L, Hessler P, et al. Protein tyrosine phosphatase 1B negatively regulates leptin signaling in a hypothalamic cell line. Molecular and Cellular Endocrinology 2002 Sep;195(1-2):109–118.

61. Chen Q, Li C, Gong Z, Chan ECY, Snyder SA, Lam SH. Common deregulated gene expression profiles and morphological changes in developing zebrafish larvae exposed to environmental-relevant high to low concentrations of glucocorticoids. Chemosphere 2017 Apr;172:429–439. http://www.sciencedirect.com/science/article/pii/S0045653517300383.

62. Inoue W, Poole S, Bristow AF, Luheshi GN. Leptin induces cyclooxygenase-2 via an interaction with interleukin-1beta in the rat brain. The European Journal of Neuroscience 2006 Oct;24(8):2233–2245.

63. Maciel FM, Sarrazin P, Morisset S, Lora M, Patry C, Dumais R, et al. Induction of cyclooxygenase-2 by parathyroid hormone in human osteoblasts in culture. The Journal of Rheumatology 1997 Dec;24(12):2429–2435.

64. Timme-Laragy AR, Karchner SI, Franks DG, Jenny MJ, Harbeitner RC, Goldstone JV, et al. Nrf2b, Novel Zebrafish Paralog of Oxidant-responsive Transcription Factor NF-E2-related Factor 2 (NRF2). Journal of Biological Chemistry 2012 Feb;287(7):4609–4627. http://www.jbc.org/lookup/doi/10.1074/jbc.M111.260125.

65. Ma Q. Role of Nrf2 in Oxidative Stress and Toxicity. Annual Review of Pharmacology and Toxicology 2013 Jan;53(1):401–426. http://www.annualreviews.org/doi/10.1146/annurev-pharmtox-011112-140320.

66. Yueh MF, Tukey RH. Nrf2-Keap1 Signaling Pathway Regulates Human UGT1A1 Expression *in Vitro* and in Transgenic *UGT1* Mice. Journal of Biological Chemistry 2007 Mar;282(12):8749–8758. http://www.jbc.org/lookup/doi/10.1074/jbc.M610790200.

67. Wang RL, Biales AD, Garcia-Reyero N, Perkins EJ, Vil-

leneuve DL, Ankley GT, et al. Fish connectivity mapping: linking chemical stressors by their mechanisms of action-driven transcriptomic profiles. BMC Genomics 2016 Jan;17(1):84. https://doi.org/10.1186/s12864-016-2406-y.

68. Woo JH, Shimoni Y, Yang WS, Subramaniam P, Iyer A, Nicoletti P, et al. Elucidating Compound Mechanism of Action by Network Perturbation Analysis. Cell 2015 Jul;162(2):441–451. https://linkinghub.elsevier.com/retrieve/pii/S0092867415006996.
69. Törönen P, Kolehmainen M, Wong G, Castrén E. Analysis of gene expression data using self-organizing maps. FEBS Letters 1999 May;451(2):142–146. http://doi.wiley.com/10.1016/S0014-5793%2899%2900524-4.
70. Tamayo P, Slonim D, Mesirov J, Zhu Q, Kitareewan S, Dmitrovsky E, et al. Interpreting patterns of gene expression with self-organizing maps: Methods and application to hematopoietic differentiation. Proceedings of the National Academy of Sciences 1999 Mar;96(6):2907–2912. http://www.pnas.org/cgi/doi/10.1073/pnas.96.6.2907.
71. Wirth H, von Bergen M, Binder H. Mining SOM expression portraits: feature selection and integrating concepts of molecular function. BioData Mining 2012 Dec;5(1). http://biodatamining.biomedcentral.com/articles/10.1186/1756-0381-5-18.
72. Driessen M, Kienhuis AS, Vitins AP, Pennings JLA, Pronk TE, Brandhof EJvd, et al. Gene expression markers in the zebrafish embryo reflect a hepatotoxic response in animal models and humans. Toxicology Letters 2014 Oct;230(1):48–56. https://linkinghub.elsevier.com/retrieve/pii/S0378427414010947.
73. Sonnack L, Klawonn T, Kriehuber R, Hollert H, Schäfers C, Fenske M. Concentration dependent transcriptome responses of zebrafish embryos after exposure to cadmium, cobalt and copper. Comparative Biochemistry and Physiology Part D: Genomics and Proteomics 2017 Dec;24:29–40. http://www.sciencedirect.com/science/article/pii/S1744117X17300588.
74. Sefer E, Kleyman M, Bar-Joseph Z. Tradeoffs between Dense and Replicate Sampling Strategies for High-Throughput Time Series Experiments. Cell Systems 2016 Jul;3(1):35–42. http://www.sciencedirect.com/science/article/pii/S2405471216302125.
75. Moncada A. Environmental Fate of Diuron. Sacramento, CA: California Department of Pesticide Regulation; 2004.
76. Felmlee MA, Morris ME, Mager DE. Mechanism-Based Pharmacodynamic Modeling. In: Reisfeld B, Mayeno AN, editors. Computational Toxicology, vol. 929 Totowa, NJ: Humana Press; 2012.p. 583–600. http://link.springer.com/10.1007/978-1-62703-050-2_21.
77. Jager T, Heugens EHW, Kooijman SALM. Making Sense of Ecotoxicological Test Results: Towards Application of Process-based Models. Ecotoxicology 2006 Apr;15(3):305–314. http://link.springer.com/10.1007/s10646-006-0060-x.
78. Greiling TMS, Clark JI. Early lens development in the zebrafish: A three-dimensional time-lapse analysis. Developmental Dynamics 2009 Sep;238(9):2254–2265. http://doi.wiley.com/10.1002/dvdy.21997.
79. Wagner DE, Weinreb C, Collins ZM, Briggs JA, Megason SG, Klein AM. Single-cell mapping of gene expression landscapes and lineage in the zebrafish embryo. Science 2018 Jun;360(6392):981–987. http://www.sciencemag.org/lookup/doi/10.1126/science.aar4362.
80. Hermsen SAB, Pronk TE, van den Brandhof EJ, van der Ven LTM, Piersma AH. Transcriptomic analysis in the developing zebrafish embryo after compound exposure: Individual gene expression and pathway regulation. Toxicology and Applied Pharmacology 2013 Oct;272(1):161–171. https://linkinghub.elsevier.com/retrieve/pii/S0041008X13002706.
81. Yee NS, Lorent K, Pack M. Exocrine pancreas development in zebrafish. Developmental Biology 2005 Aug;284(1):84–101. http://linkinghub.elsevier.com/retrieve/pii/S0012160605002897.
82. Jacobs HM, Sant KE, Basnet A, Williams LM, Moss JB, Timme-Laragy AR. Embryonic exposure to Mono(2-ethylhexyl) phthalate (MEHP) disrupts pancreatic organogenesis in zebrafish (Danio rerio). Chemosphere 2018 Mar;195:498–507. https://linkinghub.elsevier.com/retrieve/pii/S0045653517320647.
83. Sant KE, Jacobs HM, Borofski KA, Moss JB, Timme-Laragy AR. Embryonic exposures to perfluorooctanesulfonic acid (PFOS) disrupt pancreatic organogenesis in the zebrafish, Danio rerio. Environmental Pollution 2017 Jan;220:807–817. https://linkinghub.elsevier.com/retrieve/pii/S0269749116318437.
84. Imig J. Eicosanoids and renal vascular function in diseases. Clinical Science 2006 Jul;111(1):21–34. http://clinsci.org/lookup/doi/10.1042/CS20050251.
85. Yang L, Mäki-Petäjä K, Cheriyan J, McEniery C, Wilkinson IB. The role of epoxyeicosatrienoic acids in the cardiovascular system. British Journal of Clinical Pharmacology 2015 Jul;80(1):28–44.
86. Hargus SJ, Amouzedeh HR, Pumford NR, Myers TG, McCoy SC, Pohl LR. Metabolic activation and immunochemical localization of liver protein adducts of the nonsteroidal anti-inflammatory drug diclofenac. Chemical Research in Toxicology 1994 Aug;7(4):575–582.
87. Wang Y, Huang H, Wu Q. Characterization of the Zebrafish Ugt Repertoire Reveals a New Class of Drug-Metabolizing UDP Glucuronosyltransferases. Molecular Pharmacology 2014 May;86(1):62–75. http://molpharm.aspetjournals.org/cgi/doi/10.1124/mol.113.091462.
88. Boelsterli U. Diclofenac-induced liver injury: a paradigm of idiosyncratic drug toxicity. Toxicology and Applied Pharmacology 2003 Nov;192(3):307–322. http://linkinghub.elsevier.com/retrieve/pii/S0041008X03003685.
89. Lin TY, Cantley LC, DeNicola GM. NRF2 Rewires Cellular Metabolism to Support the Antioxidant Response. A Master Regulator of Oxidative Stress - The Transcription Factor Nrf2 2016 Dec;https://www.intechopen.com/books/a-master-regulator-of-oxidative-stress-the-transcription-factor-nrf nrf2-rewires-cellular-metabolism-to-support-the-antioxidant-respons
90. Gong P, Cederbaum AI. Transcription Factor Nrf2 Protects HepG2 Cells against CYP2E1 plus Arachidonic Acid-dependent Toxicity. Journal of Biological Chemistry 2006 May;281(21):14573–14579. http://www.jbc.org/lookup/doi/10.1074/jbc.M600613200.
91. McCarthy S, Somayajulu M, Sikorska M, Borowy-Borowski H, Pandey S. Paraquat induces oxidative stress and neuronal cell death; neuroprotection by water-soluble Coenzyme Q10. Toxicology and Applied Pharmacology 2004 Nov;201(1):21–31. http://www.sciencedirect.com/science/article/pii/S0041008X04002467.
92. Driessen M, Vitins AP, Pennings JLA, Kienhuis AS, Water Bvd, van der Ven LTM. A transcriptomics-based hepatotoxicity comparison between the zebrafish embryo and established human and rodent in vitro and in vivo models using cyclosporine A, amiodarone and acetaminophen. Toxicology Letters 2015 Jan;232(2):403–412. https://linkinghub.elsevier.com/retrieve/pii/S0378427414014726.
93. Galati G, Tafazoli S, Sabzevari O, Chan TS, O'Brien PJ. Idiosyncratic NSAID drug induced oxidative stress.

Chemico-Biological Interactions 2002 Nov;142(1):25–41. http://www.sciencedirect.com/science/article/pii/S0009279702000522.

94. Islas-Flores H, Gómez-Oliván LM, Galar-Martínez M, Colín-Cruz A, Neri-Cruz N, García-Medina S. Diclofenac-induced oxidative stress in brain, liver, gill and blood of common carp (Cyprinus carpio). Ecotoxicology and Environmental Safety 2013 Jun;92:32–38. http://www.sciencedirect.com/science/article/pii/S0147651313000419.
95. Dreyer MG, Juge-Aubry CE, Gabay C, Lang U, Rohner-Jeanrenaud F, Dayer JM, et al. Leptin activates the promoter of the interleukin-1 receptor antagonist through p42/44 mitogen-activated protein kinase and a composite nuclear factor kappaB/PU.1 binding site. Biochemical Journal 2003 Mar;370(2):591–599. http://biochemj.org/lookup/doi/10.1042/bj20021270.
96. Fu Q, Manolagas SC, O'Brien CA. Parathyroid Hormone Controls Receptor Activator of NF- B Ligand Gene Expression via a Distant Transcriptional Enhancer. Molecular and Cellular Biology 2006 Sep;26(17):6453–6468. http://mcb.asm.org/cgi/doi/10.1128/MCB.00356-06.
97. Wein MN. Parathyroid Hormone Signaling in Osteocytes. JBMR Plus 2018 Jan;2(1):22–30. http://doi.wiley.com/10.1002/jbm4.10021.
98. Du Z, Wei L, Murti A, Pfeffer SR, Fan M, Yang CH, et al. Non-conventional signal transduction by type 1 interferons: The NF-kB pathway. Journal of Cellular Biochemistry 2007 Dec;102(5):1087–1094. http://doi.wiley.com/10.1002/jcb.21535.
99. Tegeder I, Pfeilschifter J, Geisslinger G. Cyclooxygenase-independent actions of cyclooxygenase inhibitors. The FASEB Journal 2001 Oct;15(12):2057–2072. http://www.fasebj.org/doi/10.1096/fj.01-0390rev.
100. Cheng L, Yu Y, Zhang Q, Szabo A, Wang H, Huang XF. Arachidonic acid impairs hypothalamic leptin signaling and hepatic energy homeostasis in mice. Molecular and Cellular Endocrinology 2015 Sep;412:12–18. https://linkinghub.elsevier.com/retrieve/pii/S0303720715002269.
101. Lopez I, Pineda C, Raya AI, Rodriguez-Ortiz ME, Diaz-Tocados JM, Rios R, et al. Leptin directly stimulates parathyroid hormone secretion. Endocrine 2017 Jun;56(3):675–678. http://link.springer.com/10.1007/s12020-016-1207-z.
102. Xu T, Zhao J, Yin D, Zhao Q, Dong B. High-throughput RNA sequencing reveals the effects of 2,2,4,4-tetrabromodiphenyl ether on retina and bone development of zebrafish larvae. BMC Genomics 2015 Jan;16(1). https://www.ncbi.nlm.nih.gov/pmc/articles/PMC4312473/.
103. Kodavanti PRS, Derr-Yellin EC. Differential effects of polybrominated diphenyl ethers and polychlorinated biphenyls on [3H]arachidonic acid release in rat cerebellar granule neurons. Toxicological Sciences: An Official Journal of the Society of Toxicology 2002 Aug;68(2):451–457.
104. Zhao X, Ren X, Ren B, Luo Z, Zhu R. Life-cycle exposure to BDE-47 results in thyroid endocrine disruption to adults and offsprings of zebrafish (Danio rerio). Environmental Toxicology and Pharmacology 2016 Dec;48:157–167. http://www.sciencedirect.com/science/article/pii/S1382668916302587.
105. Subramanian A, Narayan R, Corsello SM, Peck DD, Natoli TE, Lu X, et al. A Next Generation Connectivity Map: L1000 Platform and the First 1,000,000 Profiles. Cell 2017 Nov;171(6):1437–1452.e17. https://linkinghub.elsevier.com/retrieve/pii/S0092867417313090.
106. Wang P, Xia P, Yang J, Wang Z, Peng Y, Shi W, et al. A Reduced Transcriptome Approach to Assess Environmental Toxicants Using Zebrafish Embryo Test. Environmental Science & Technology 2018 Jan;52(2):821–830. http://pubs.acs.org/doi/10.1021/acs.est.7b04073.
107. Liberzon A, Subramanian A, Pinchback R, Thorvaldsdottir H, Tamayo P, Mesirov JP. Molecular signatures database (MSigDB) 3.0. Bioinformatics 2011 Jun;27(12):1739–1740. https://academic.oup.com/bioinformatics/article-lookup/doi/10.1093/bioinformatics/btr260.
108. Davis AP, Grondin CJ, Johnson RJ, Sciaky D, King BL, McMorran R, et al. The Comparative Toxicogenomics Database: update 2017. Nucleic Acids Research 2017 Jan;45(D1):D972–D978. https://academic.oup.com/nar/article-lookup/doi/10.1093/nar/gkw838.
109. Altenburger R, Scholz S, Schmitt-Jansen M, Busch W, Escher BI. Mixture Toxicity Revisited from a Toxicogenomic Perspective. Environmental Science & Technology 2012 Mar;46(5):2508–2522. http://pubs.acs.org/doi/10.1021/es2038036.
110. R Core Team, R: A Language and Environment for Statistical Computing. Vienna, Austria: R Foundation for Statistical Computing; 2017. https://www.R-project.org/.
111. Kauffmann A, Huber W. Microarray data quality control improves the detection of differentially expressed genes. Genomics 2010 Mar;95(3):138–142. http://linkinghub.elsevier.com/retrieve/pii/S0888754310000042.
112. Leek JT, Johnson WE, Parker HS, Jaffe AE, Storey JD. The sva package for removing batch effects and other unwanted variation in high-throughput experiments. Bioinformatics 2012 Mar;28(6):882–883. https://academic.oup.com/bioinformatics/article-lookup/doi/10.1093/bioinformatics/bts034.
113. Grubbs FE. Sample Criteria for Testing Outlying Observations. The Annals of Mathematical Statistics 1950 Mar;21(1):27–58. http://projecteuclid.org/euclid.aoms/1177729885.
114. Wood SN. Thin plate regression splines. Journal of the Royal Statistical Society: Series B (Statistical Methodology) 2003 Feb;65(1):95–114. http://doi.wiley.com/10.1111/1467-9868.00374.
115. Wehrens R, Buydens LMC. Self- and Super-organizing Maps in *R* : The **kohonen** Package. Journal of Statistical Software 2007;21(5). http://www.jstatsoft.org/v21/i05/.
116. Scrucca L. On Some Extensions to GA Package: Hybrid Optimisation, Parallelisation and Islands Evolution. The R Journal 2017;8(1):205–233.
117. Betebenner DW. randomNames: Function for Generating Random Names and a Dataset; 2017. https://cran.r-project.org/package=randomNames.
118. Yu G, Wang LG, Han Y, He QY. clusterProfiler: an R Package for Comparing Biological Themes Among Gene Clusters. OMICS: A Journal of Integrative Biology 2012 May;16(5):284–287. http://www.liebertpub.com/doi/10.1089/omi.2011.0118.
119. Duan QY, Gupta VK, Sorooshian S. Shuffled complex evolution approach for effective and efficient global minimization. Journal of Optimization Theory and Applications 1993 Mar;76(3):501–521. http://link.springer.com/10.1007/BF00939380.
120. Andrews FT, Croke BFW, Jakeman AJ. An open software environment for hydrological model assessment and development. Environmental Modelling & Software 2011 Oct;26(10):1171–1185. http://linkinghub.elsevier.com/retrieve/pii/S1364815211001046.
121. Chang W, Cheng J, Allaire JJ, Xie Y, McPherson J. shiny: Web Application Framework for R; 2018. https://CRAN.R-project.org/package=shiny.
122. Xie Y. Dynamic documents with R and Knitr. Chapman & Hall/CRC the R series, Boca Raton: CRC Press, Taylor &

Francis; 2014.

Click here to access/download
Supplementary Material
supplementary_methods.html

Supplementary Figures

Click here to access/download
Supplementary Material
supplementary_figures.pdf

Table S1

Click here to access/download
Supplementary Material
TableS1_PublicData.txt

Table S2

Click here to access/download
Supplementary Material
TableS2_ClusterTable.txt

Table S3

Click here to access/download
Supplementary Material
TableS3_Enrichments_GO.txt

Table S4

Click here to access/download
Supplementary Material
TableS4_Enrichments_interpro.txt

Table S5

Click here to access/download
Supplementary Material
TableS5_Enrichments_reactome.txt

Table S6

Click here to access/download
Supplementary Material
TableS6_Enrichments_ZFIN.txt

Table S7

Click here to access/download
Supplementary Material
TableS7_significant_toxnodes_diuron.txt

Table S8

Click here to access/download
Supplementary Material
TableS8_significant_toxnodes_diclofenac.txt

Table S9

Click here to access/download
Supplementary Material
TableS9_significant_toxnodes_naproxen.txt

Supplementary Methods Source

Click here to access/download

Supplementary Material

supplementary_info.Rmd

Supplementary Methods Source

Click here to access/download
Supplementary Material
01ExperimentalProcedure.Rmd

Supplementary Methods Source

Click here to access/download
Supplementary Material
02LoadingPreprocessing.Rmd

Supplementary Methods Source

Click here to access/download
Supplementary Material
03CreateToxUniverse.Rmd

Click here to access/download
Supplementary Material
04RegressionModeling.Rmd

Supplementary Methods Source

Click here to access/download
Supplementary Material
bibliography.bib

Supplementary Methods Source

Click here to access/download
Supplementary Material
plots_tables.R
