# Supplementary material for: Map and model—moving from observation to prediction in toxicogenomics
Source: Gigascience. 2019 May 29;8(6):giz057. doi: 10.1093/gigascience/giz057 (PMC6539241; doi:10.1093/gigascience/giz057)
Supplement: giz057_Supplementary_Figures [file giz057_supplementary_figures.pdf]

Supplementary Figures for 'Map and Model – moving from observation to prediction in toxicogenomics'

Andreas Schüttler, Rolf Altenburger, Madeleine Ammar, Marcella Bader-Blukott, Gianina Jakobs, Johanna Knapp, Janet Krüger, Kristin Reiche, Gi-Mick Wu, Wibke Busch

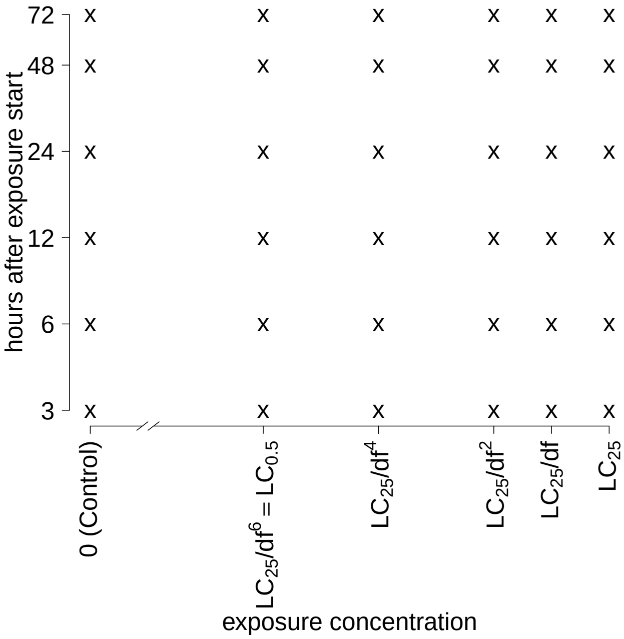

Figure S1: Experimental design for transcriptome experiments applied in our study. Each "x" indicates one sampling condition, df = compound specific dilution factor

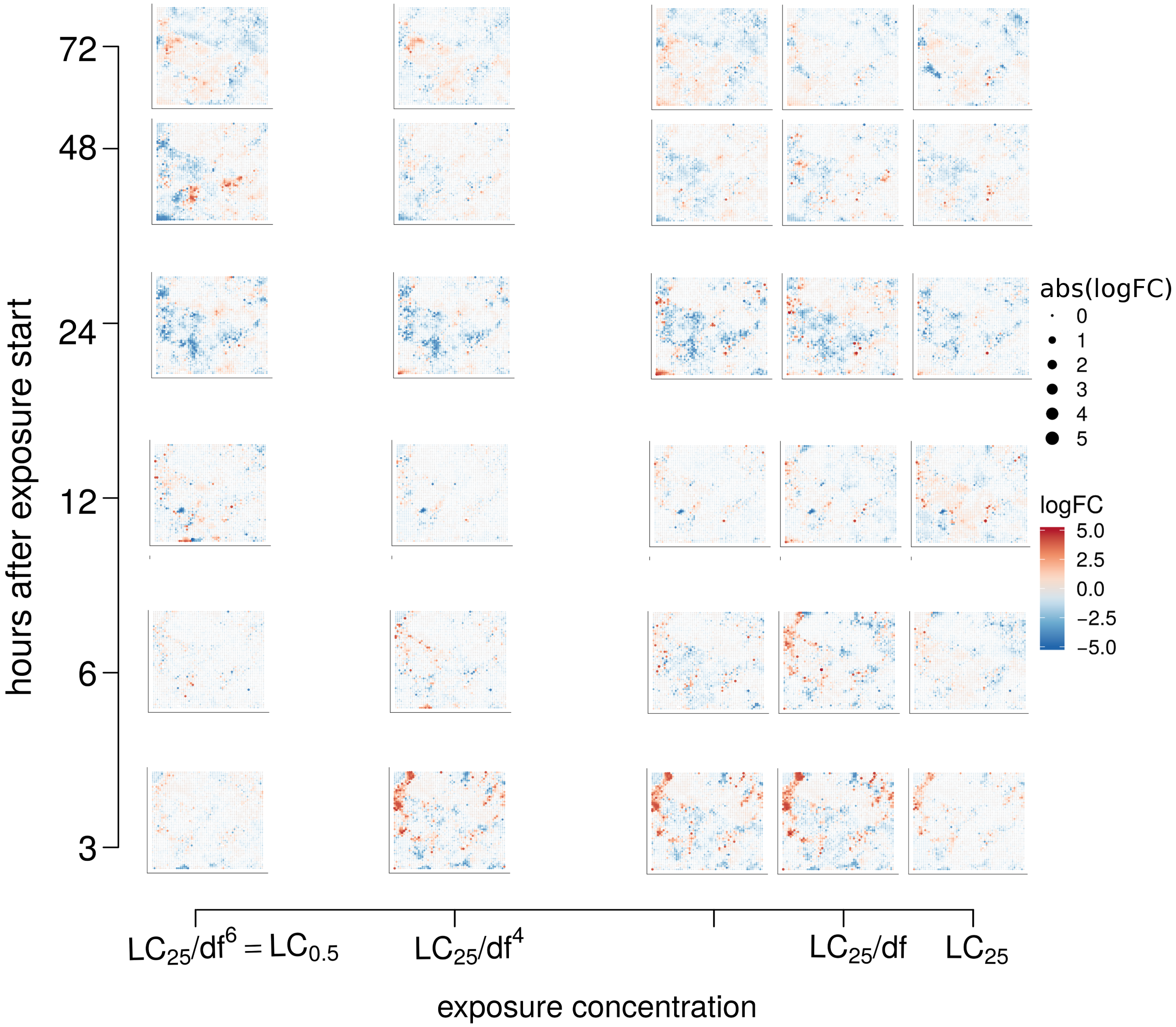

Figure S2: Toxicodynamic fingerprint for diuron projected on the toxicogenomic universe. Shown is the representative logFC fingerprint for each sampled time point/exposure concentration.

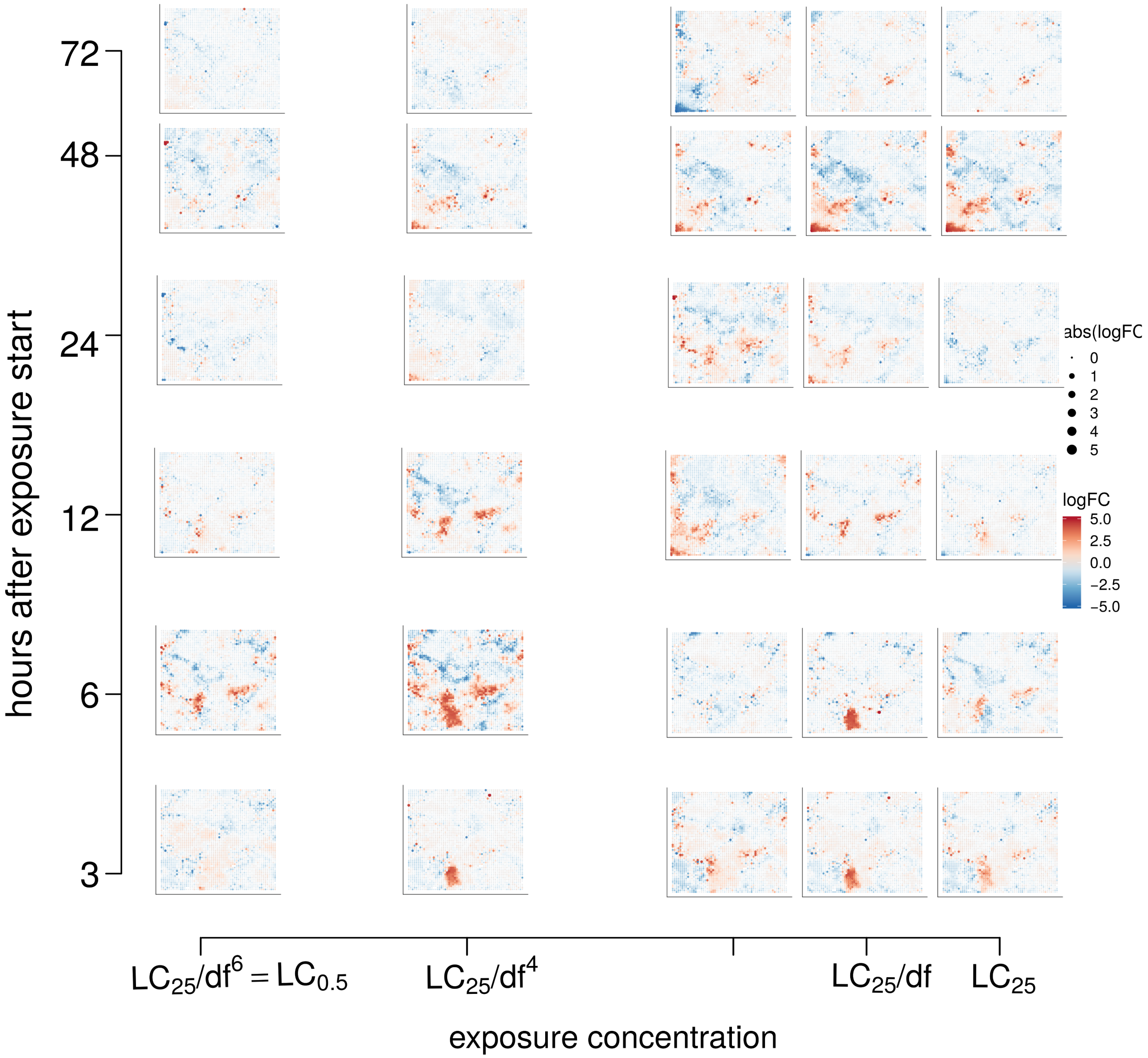

Figure S3: Toxicodynamic fingerprint for diclofenac projected on the toxicogenomic universe. Shown is the representative logFC fingerprint for each sampled time point/exposure concentration.

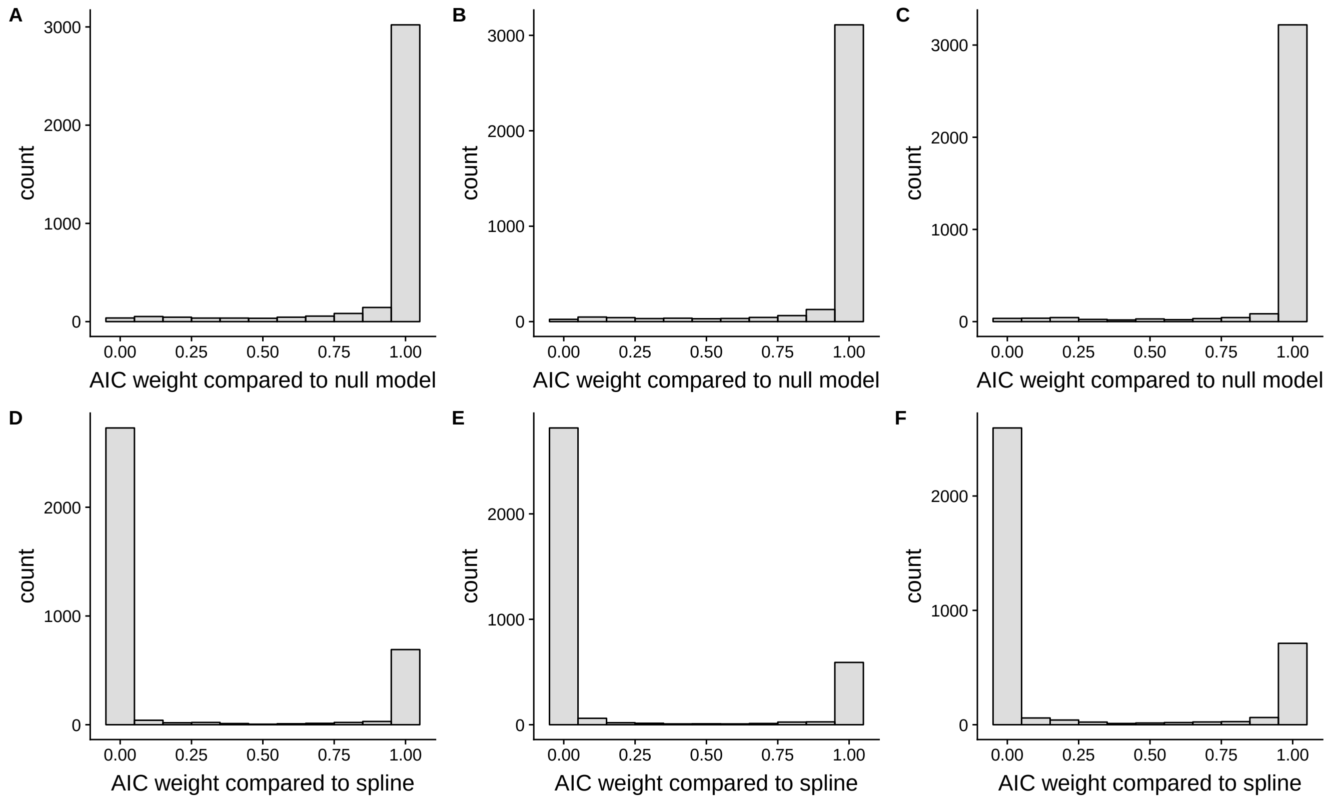

Figure S4: Histogram of AIC weights of fitted mobi-CTR model in comparison with null-model (A: diuron; B: diclofenac; C: naproxen) and spline fit (D: diuron, E: diclofenac, F: naproxen)

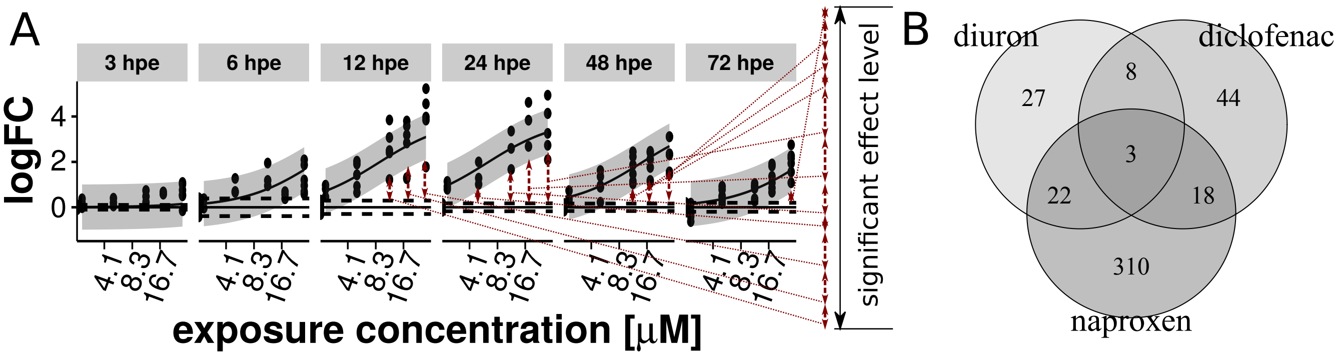

Figure S5: A: Demonstration of significant effect level calculation for a toxnode. B: Overlapping significant toxnodes for investigated model substances

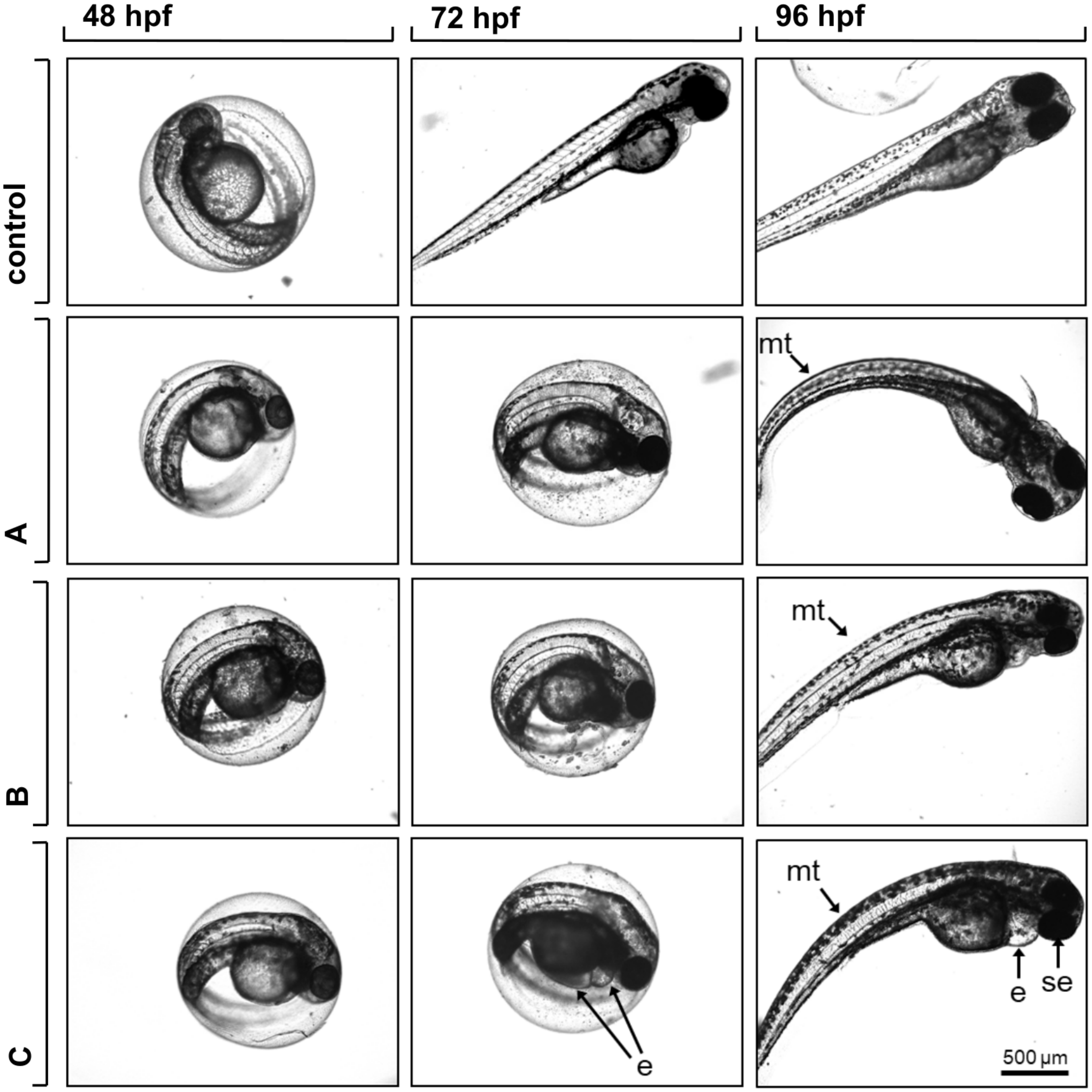

Figure S6: Morphological effects of zebrafish embryo exposure to A: diuron (29.44 μM), B:diclofenac (7.36 μM), C: naproxen (309.14 μM) for three different treatment periods (24-48 hpf, 24-72 hpf, 24-96 hpf). Observed effects are e: edema, mt: malformation of tail, se: smaller eyes.

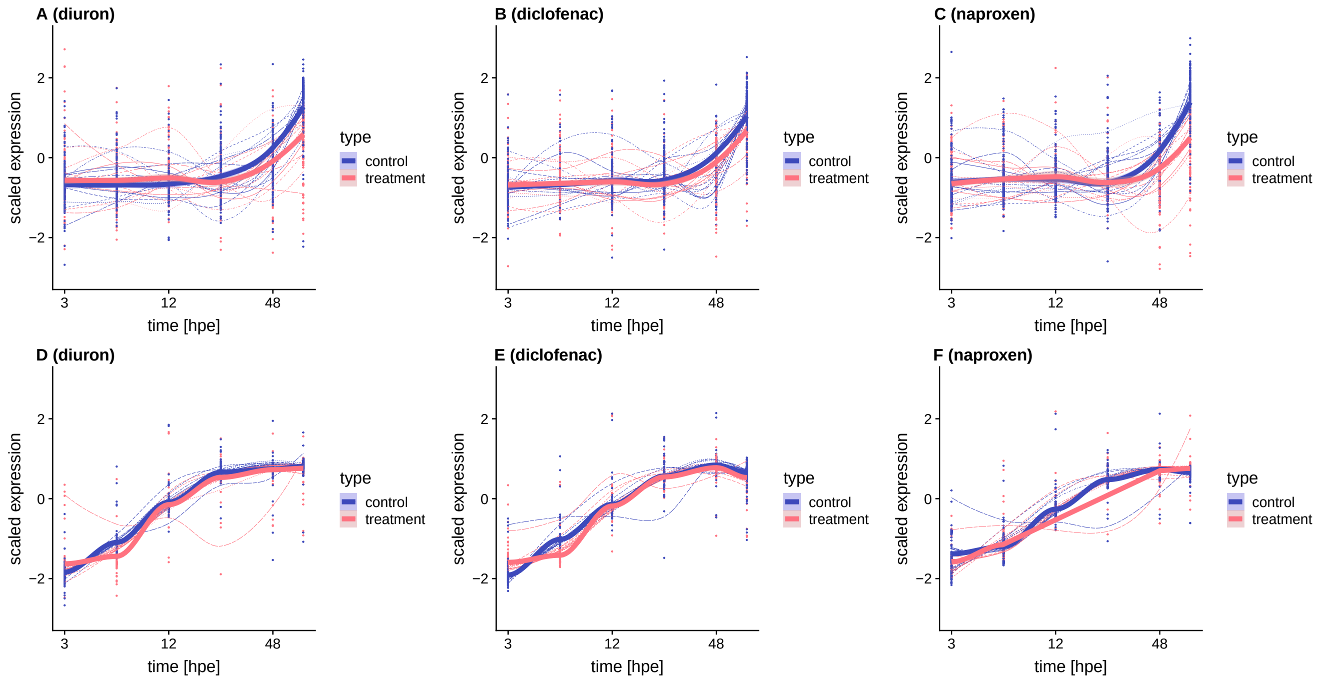

Figure S7: Regulation of genes in the Pankreas-Cluster "John" (A-C) and Crystallin-Cluster "Trae". log_2-Expression values for the highest measured concentrations were centered and scaled for each ProbeID. Thin lines represent spline fits for each Probe, while the thick lines represent a spline fit for all control respectively treatment measurements.

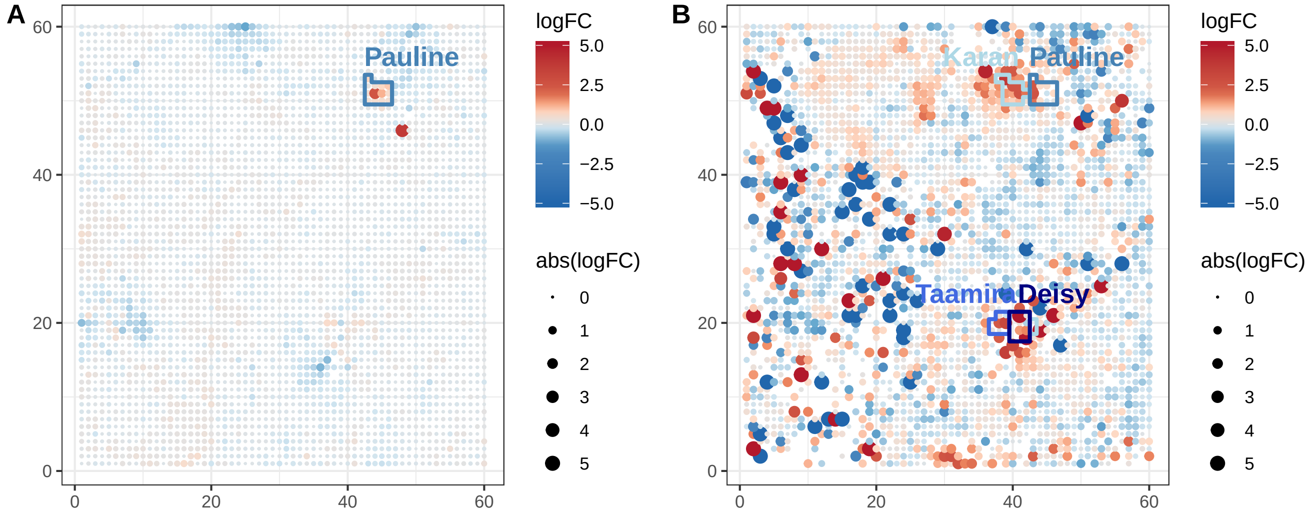

Figure S8: Toxicogenomic fingerprints projected on the toxicogenomic universe. A: paraquat; B: BDE-47.

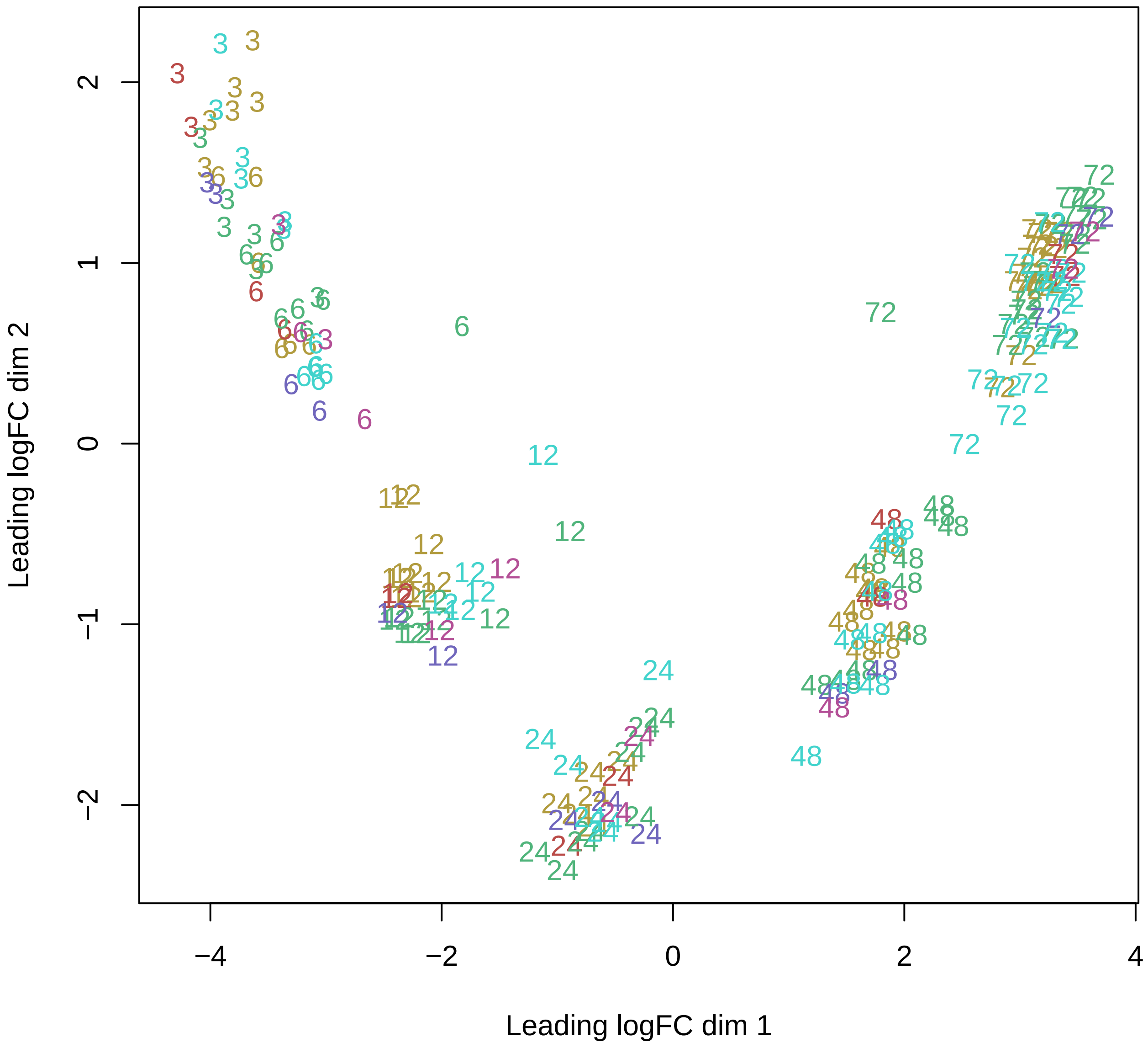

Figure S9: Multidimensional scaling plot of all transcriptome samples for diuron, diclofenac and naproxen exposure between 24 and 96 hours post fertilization. Numbers: exposure duration in hours; diuron controls (■); diuron treatments (■); diclofenac controls (■); diclofenac treatments (■); naproxen controls (■); naproxen treatments (■); the plot shows the relation between the samples and illustrates that samples cluster according to embryonic stage.
