# Supplementary material for: Map and model—moving from observation to prediction in toxicogenomics
Source: Gigascience. 2019 May 29;8(6):giz057. doi: 10.1093/gigascience/giz057 (PMC6539241; doi:10.1093/gigascience/giz057)
Supplement: giz057_Supplementary_Methods [file giz057_supplementary_methods.html]

Supplementary Methods for Map and Model � moving from observation to prediction in toxicogenomics


Code 

- Show All Code
- Hide All Code

# Supplementary Methods for �Map and Model � moving from observation to prediction in toxicogenomics�

#### *Andreas Sch�ttler, Rolf Altenburger, Madeleine Ammar, Marcella Bader-Blukott, Gianina Jakobs, Johanna Knapp, Janet Kr�ger, Kristin Reiche, Gi-Mick Wu, Wibke Busch*

# 

## About

In the study **Sch�ttler et al. (2019): Map and Model - moving from observation to prediction in toxicogenomics** dynamic toxicogenomic fingerprints of three selected substances on the zebrafish embryo were retrieved.

This document gives additional information about the experimental and the data analysis workflow. The document is created with the help of the package �knitr� (Xie 2017). It is structured into 6 parts:

1. *Experiment*: Information regarding experimental procedure
   - Exposure
   - Analysis of morphological data
   - RNA extraction and hybridization
   - Toxikokinetic measurements
2. *Data*: Loading and preprocessing the microarray data
   - Data import
   - Normalisation
   - Further data preprocessing
   - QC
   - Calculation of logFC
3. *Universe*: Creating the *�toxicogenomic universe�*
   - Spline smooth
   - Infer SOM
   - k-means clustering of nodes
4. *CTR-model*: Regression modeling/Identifying significantly regulated toxnodes
   - mobi-CTR model
   - Parameter boundaries
   - Model fitting
   - Identification of significant toxnodes
5. *Info*: Information about R-environment
6. *References*

The workflow is based on the custom R-package `toxprofileR`. The package is accessible on GitLab ( https://git.ufz.de/itox/toxprofileR). It mainly builds on the packages `limma`(Ritchie et al. 2015), `biomaRt`(Durinck et al. 2009), `kohonen`(Wehrens and Buydens 2007), and `hydromad`(Andrews, Croke, and Jakeman 2011). It currently is only suitable for analysis of Oaklabs Zebrafish XS Microarrays.

### Installation of *toxprofileR*

Before we can run the workflow we need to install the package `toxprofileR`. We can install the most recent version directly from the GitLab repository. The `devtools` package is required for this.

```
devtools::install_git("https://git.ufz.de/itox/toxprofileR.git")
```

## Experiment

### Experimental procedure

#### Exposure

Wild-type adult zebrafish, originally received from OBI petshop (Leipzig, Germany) were kept in 120 L fish tanks containing carbon-filtered tap water at 26 �C under a 10h:14h dark-light cycle. Eggs were collected approximately 1 hour after light onset and inspected using a light microscope. Fertilized eggs were incubated at 26 �C, while unfertilized eggs were discarded. For lethality experiments, which were conducted to find adequate exposure concentrations for transcriptome experiments, three embryos with an age of 24 hours post fertilization (hpf) were distributed into a 7.5 mL GC vials, each containing 6 mL exposure or control medium. Regarding treatment conditions three technical replicates and regarding control conditions six technical replicates were analysed. Vials were sealed and incubated in a climate chamber shaking (climate chamber: V�tsch 1514, V�tsch Industrietechnik GmBH (Balingen-Frommern); shaker: Edmund B�hler SM-30 Control, 26 �C, 75 rpm, 12h:12h light:dark) till 96 hpf. At 48 hpf, 72 hpf and 96 hpf sublethal and lethal effects were recorded. For transcriptome experiments 20 embryos per replicate were transferred to two 20 mL GC vials at 24 hpf. A volume of 18 mL of exposure medium was added to each vial and vials were sealed and incubated in a climate chamber (same settings as above) until sampling. Embryos were exposed to a range of 5 different concentrations of the toxicant and RNA was extracted at 3, 6, 12, 24, 48, 72 hours after start of exposure (hpe). Three substances were used for exposure experiments: diuron (CAS:330-54-1 ; purity: 99.6%; charge: #SZBB265XV ; supplier: Fluka ), diclofenac sodium salt (CAS:15307-79-6 ; purity: n.A.; charge: #BCBP9916V ; supplier: Sigma ), and naproxen sodium salt (CAS: 26159-34-2; purity: 98-102%; charge: #MKBV4690V ; supplier: Sigma). Exposure medium for all experiments was prepared by dissolving the substance in freshly prepared ISO-water (ISO 7346-3: 79.99 mM CaCl2�2H2O, 20.00 mM MgSO4�7H2O, 30.83 mM NaHCO3, 3.09 mM KCl; pH 7.4, oxygenized) (diclofenac, naproxen) or in 0.1 % methanol (CAS:67-56-1 ; purity: HPLC Grade, charge:, supplier: J.T. Baker) as solubilising agent (diuron). For the diuron exposure we used 0.1 % methanol as solvent control.

#### Analysis of morphological data

To determine an accurate value for \(LC\_{25}\) and \(LC\_{0.5}\), concentration response relationships were modeled from experimental data using established toxicological models (Scholze et al. 2001). Three models, namely *logit*, *weibull* and *generalized logit* were fit to experimental data using maximum likelihood estimation. Afterwards the best fitting model was selected by comparing the \(AIC\_c\). The best fitting model for each substance was used to determine the \(LC\_{25}\) and \(LC\_{0.5}\).

##### Diuron

```
library("toxprofileR")

substance <- "diuron"
designtime <- 48  # diuron response is not time dependent, so we chose the first time point as design time point

responsedf <- read.csv(paste0("./data/", substance, "/ApicalData/data_lethal_", 
    substance, "_aggregated.csv"), header = T, sep = "\t", quote = "")
apicalfit <- toxprofileR::fit_apical(responsedf = responsedf, plot = T, designtime = designtime)
save(apicalfit, file = paste0("./data/", substance, "/ApicalData/apicalfit.Rd"))
```

Figure 1: Morphological effects of diuron exposure

From the fitted model, the concentrations for the transcriptome measurements are derived: Concentrations for diuron : 5.9; 9.9; 16.7; 21.6; 28.1 �mol/L

##### Diclofenac

```
library("toxprofileR")

substance <- "diclofenac"
designtime <- 96

responsedf <- read.csv(paste0("./data/", substance, "/ApicalData/data_lethal_", 
    substance, "_aggregated.csv"), header = T, sep = "\t", quote = "")
apicalfit <- toxprofileR::fit_apical(responsedf = responsedf, plot = T, designtime = designtime)
save(apicalfit, file = paste0("./data/", substance, "/ApicalData/apicalfit.Rd"))
```

Figure 2: Morphological effects of diclofenac exposure

From the fitted model, the concentrations for the transcriptome measurements are derived: Concentrations for diclofenac : 5.1; 5.8; 6.5; 6.9; 7.4 �mol/L

##### Naproxen

```
library("toxprofileR")

substance <- "naproxen"
designtime <- 96

responsedf <- read.csv(paste0("./data/", substance, "/ApicalData/data_lethal_", 
    substance, "_aggregated.csv"), header = T, sep = "\t", quote = "")
apicalfit <- toxprofileR::fit_apical(responsedf = responsedf, plot = T, designtime = designtime)
save(apicalfit, file = paste0("./data/", substance, "/ApicalData/apicalfit.Rd"))
```

Figure 3: Morphological effects of naproxen exposure

From the fitted model, the concentrations for the transcriptome measurements are derived:

Concentrations for naproxen : 169.5; 206.5; 251.6; 277.7; 306.5 �mol/L `

#### RNA extraction and hybridization

At observation time points, two vials of embryos (20 in total) were transferred into Eppendorf tubes. RNA was extracted by addition of Trizol and homogenization using a T10 basic Ultra-Turrax (IKA, Werke GmbH & Co. KG, Germany) for 20 sec at maximum speed. RNA isolation was performed using a pipetting robot (Microlab Star, Hamilton Life Science Robotics, Germany) following the manual provided for Total RNA Extraction Kit MagMAX 96 for microarrays and conducted in a 96-well plate. Quality of isolated RNA was assessed using a Bioanalyzer (Agilent 2100 Technologies, Waldborn) and the Agilent RNA 6000 Nano Kit. RNA samples were used for further processing if RIN values derived from ribosomal RNA absorption adopted values > 7 and calculated concentrations exceeded 25 ng/�L. All RNA samples were diluted to a concentration level of 25 ng/�L(10 �L in total) by addition of RNAse free water. Thereof, 2.3 �L (57 ng RNA) were utilised as starting amount of RNA for spike mix preparation.

Transcript abundance was measured with microarray analysis using Oaklabs ArrayXS Zebrafish microarray slides (XS-200104, oak-labs.com, Germany). Microarray experiments were performed using the Agilent Low Input Quick Amp WT Labeling Kit according to the Agilent One-Color Microarray-Based Exon Analysis Protocol (V2.0, August 2015). This protocol included introduction of spike-in RNA, RNA transcription and amplification into complementary DNA, and cDNA transcription and amplification into cRNA with simultaneous incorporation of Cy3 (fluorescently labelled cytidine nucleotide). The cRNA was fragmented and hybridized to Oaklabs ArrayXS Zebrafish microarray slides using the Agilent Hybridization kit and protocol as well as Agilent hybridization oven and chambers. Subsequently, microarray slides were washed and scanned with the Agilent High-Resolution Microarray Scanner according to the Agilent protocol. Intensity values were extracted from captured images using Agilent Feature Extraction Software (Version 11.5.1.1). RNA samples in the Diclofenac experiment were partly processed by Oaklabs GmbH (Henningsdorf, Germany). Potential batch effect, caused by handling the samples in two different laboratories was accounted for during data analysis.

#### Toxicokinetics

For the determination of internal chemical concentrations 3 replicates of 9 embryos per replicate were exposed and harvested after the different respective exposure durations. Embryos that were not hatched at the time point of harvest were not dechorionated for this experiment. Embryos were transferred in a 2 mL safe lock Eppendorf tube, washed with medium and dried from remaining water using a microliter pipette. The embryos were shock-frozen with liquid nitrogen and stored at -80�C until extraction. In dependence on the substance either acetonitrile, methanol-water-mix or pure water (see table below) was added to each sample respectively after homogenizing with a glass rod. The homogenate underwent 5 minutes sonication in an ultrasonic bath (Sonorex RK 512 H ; Bandelin Electronic) and 4 hours incubation at 20�C and 1000 rpm (Thriller; Peqlab) and were finally centrifuged 10 minutes at 10000 rpm and 4�C (Model 2K15; Sigma). The supernatants were separated from the matrix and used for the chemical analysis of the internal chemical concentrations. The chemical analyses were performed either on a HPLC system (Merck Hitachi � La Chrom) with diode array (DAD; model L7450) or ultraviolet-detector (UV; model L-7400). A calibration curve was acquired in the respective medium. No additional spike-in or internal standard was used for calibration.

| Procedure | Diuron | Diclofenac sodium | Naproxen sodium |
| --- | --- | --- | --- |
| Solvent extraction | 100 % acetonitrile | 20/80 % - methanol / water | 100 % pure water |
| Extraction volume | 1000 �L | 200 �L | 500 �L |
| Chemical analysis | HPLC � DAD | HPLC � UV | HPLC - DAD |
| Mobile phase | 30% acetonitrile / 70% water | 35% acetonitrile / 65% 10 mM formic acid sodium salt (pH=6) | 70% 20 mM phosphate buffer (pH=7) / 30% water |
| Flow rate | 0.5 mL/min | 0.5 mL/min | 0.5 mL/min |
| Column | Merck LiChroCART 125-4 Merck LiChrospher 100/RP-18 endcapped (5 �m) | Merck LiChroCART 125-4 Merck LiChrospher 100/RP-18 endcapped (5 �m) | Merck LiChroCART 125-4 Merck LiChrospher 100/RP-18 endcapped (5 �m) |
| Column temperature | 25�C | 25�C | 25�C |
| Wavelength quantification | Ex 252 nm, Em 372 nm | 280 nm | Ex 230 nm, Em 400 nm |

```
library("dplyr")

for (substance in c("diuron", "diclofenac", "naproxen")) {
    exposurec <- read.csv(file = paste0("./data/", substance, "/tkData/exposure_concentrations_", 
        substance, ".csv"), header = T, sep = "\t", quote = "", as.is = T)
    
    exposurec_mean <- aggregate.data.frame(exposurec$concentration_umol_l, by = list(time_hpe = exposurec$time_hpe, 
        nominal_concentration_value = exposurec$nominal_concentration_value, 
        experiment_date = exposurec$experiment_date, comment = exposurec$comment), 
        FUN = mean, na.rm = T)
    
    
    
    colnames(exposurec_mean)[5] <- "concentration_umol_l"
    
    exposurec_se <- dplyr::summarise(group_by(exposurec_mean[!is.na(exposurec_mean$concentration_umol_l), 
        ], time_hpe, nominal_concentration_value), mean = mean(concentration_umol_l), 
        sd = sd(concentration_umol_l), N = length(concentration_umol_l), se = sd(concentration_umol_l)/sqrt(length(concentration_umol_l)))
    
    
    assign(x = paste0("exposurec_", substance, "_se"), value = exposurec_se)
    
    assign(x = paste0("exposurec_", substance), value = exposurec_mean)
    
    
}

library("cowplot")

exposurec_plot_diuron <- ggplot(data = exposurec_diuron[exposurec_diuron$nominal_concentration_value == 
    20, ]) + geom_smooth(aes(x = time_hpe, y = concentration_umol_l), se = F) + 
    geom_point(data = exposurec_diuron_se[exposurec_diuron_se$nominal_concentration_value == 
        20, ], aes(x = time_hpe, y = mean)) + geom_errorbar(data = exposurec_diuron_se[exposurec_diuron_se$nominal_concentration_value == 
    20, ], aes(x = time_hpe, ymin = mean - se, ymax = mean + se)) + ylim(c(0, 
    20)) + theme(plot.margin = unit(c(20.5, 5.5, 5.5, 5.5), "pt")) + ylab("concentration [�mol/L]") + 
    xlab("time [hpe]")  #+scale_x_log10(breaks = c(3,6,12,24,48,96), limits = c(1.5,96))

exposurec_plot_diclofenac <- ggplot(data = exposurec_diclofenac[exposurec_diclofenac$nominal_concentration_value == 
    6.6, ]) + geom_smooth(aes(x = time_hpe, y = concentration_umol_l), se = F) + 
    geom_point(data = exposurec_diclofenac_se[exposurec_diclofenac_se$nominal_concentration_value == 
        6.6, ], aes(x = time_hpe, y = mean)) + geom_errorbar(data = exposurec_diclofenac_se[exposurec_diclofenac_se$nominal_concentration_value == 
    6.6, ], aes(x = time_hpe, ymin = mean - se, ymax = mean + se)) + ylim(c(0, 
    8)) + theme(plot.margin = unit(c(20.5, 5.5, 5.5, 5.5), "pt")) + ylab("concentration [�mol/L]") + 
    xlab("time [hpe]")

exposurec_plot_naproxen <- ggplot(data = exposurec_naproxen[exposurec_naproxen$nominal_concentration_value == 
    135, ]) + geom_smooth(aes(x = time_hpe, y = concentration_umol_l), se = F) + 
    geom_point(data = exposurec_naproxen_se[exposurec_naproxen_se$nominal_concentration_value == 
        135, ], aes(x = time_hpe, y = mean)) + geom_errorbar(data = exposurec_naproxen_se[exposurec_naproxen_se$nominal_concentration_value == 
    135, ], aes(x = time_hpe, ymin = mean - se, ymax = mean + se)) + ylim(c(0, 
    160)) + # xlim(c(0,74)) +
theme(plot.margin = unit(c(20.5, 5.5, 5.5, 5.5), "pt")) + ylab("concentration [�mol/L]") + 
    xlab("time [hpe]")

plot_grid(exposurec_plot_diuron, exposurec_plot_diclofenac, exposurec_plot_naproxen, 
    labels = c("Diuron", "Diclofenac", "Naproxen"), ncol = 3)
```

Figure 4: Concentrations measured in exposure medium

## Data

### Data processing

This step consists of importing the raw-data into R, normalization, quality control, and calculation of \(logFC\).

#### Data import

First, we create a folder in our working directory called �data� and a folder for each substance tested, where we place the raw data-output from the feature-extraction software.

For each substance we need a tab-separated target file in `.csv` format containing the following columns:

- `FileName`: Names of the raw data file
- `sample_ID`: ID of experimental sample
- `substance`: Name of substance
- `concentration_level`: �Control�, �C1�, �C2�, �, �Cn� for increasing concentrations applied
- `concentration_umol_l`: Concentration of applied substance in �mol/L
- `time_hpe`: Time of measurement after exposure
- `type`: �treatment�, �control� or �recovery�
- `comment`: additional comments
- `scan_ID`: scanID (1 if there was only one scan)

Next we can use the function `importData` to import the raw data into R and remove outlier arrays.

Here we import data from exposure experiments with the three substances diuron, diclofenac and naproxen.

##### Diuron

```
library("toxprofileR")

substance <- "diuron"

targetfile = paste0("./data/", substance, "/targetsfile_", substance, ".csv")
datadir = paste0("./data/", substance, "/ArrayData/")

assign(paste("data", substance, sep = "_"), value = toxprofileR::importData(targetfile = targetfile, 
    datadir = datadir, output = T, removeOutliers = T, qc_coeff = c(ks = 3, 
        sum = 3, iqr = 3, q = 3, d = 1), qc_sum = 1))
```

```
## detected diuron_C2_72_recovery as outlier
```

```
data_diuron$targets
```

Figure 5: QC plots for diuron arrays

##### Diclofenac

```
library("toxprofileR")

substance <- "diclofenac"

targetfile = paste0("./data/", substance, "/targetsfile_", substance, ".csv")
datadir = paste0("./data/", substance, "/ArrayData/")

assign(paste("data", substance, sep = "_"), value = toxprofileR::importData(targetfile = targetfile, 
    datadir = datadir, output = T, removeOutliers = T, qc_coeff = c(ks = 3, 
        sum = 3, iqr = 3, q = 3, d = 1), qc_sum = 1))
```

```
## detected diclofenac_C5_6_treatment as outlier
## detected diclofenac_C1_48_treatment as outlier
## detected diclofenac_C3_72_treatment as outlier
## detected diclofenac_C3_72_treatment.1 as outlier
## detected diclofenac_C4_72_treatment as outlier
## detected diclofenac_C4_72_treatment.1 as outlier
```

```
data_diclofenac$targets
```

Figure 6: QC plots for diclofenac arrays

##### Naproxen

```
library("toxprofileR")

substance <- "naproxen"

targetfile = paste0("./data/", substance, "/targetsfile_", substance, ".csv")
datadir = paste0("./data/", substance, "/ArrayData/")

assign(paste("data", substance, sep = "_"), value = toxprofileR::importData(targetfile = targetfile, 
    datadir = datadir, output = T, removeOutliers = T, qc_coeff = c(ks = 3, 
        sum = 3, iqr = 3, q = 3, d = 1), qc_sum = 1))
```

```
## detected naproxen_C4_72_recovery as outlier
## detected naproxen_C5_12_treatment as outlier
## detected naproxen_C5_24_treatment as outlier
```

```
data_naproxen$targets
```

Figure 7: QC plots for naproxen arrays

Note: The data contains some additional measurements from recovery experiments which were not considered in this workflow.

#### Normalisation

Next, we normalise the data between the arrays. Here we normalize all three datasets together, using cyclic-loess normalisation. However normalisation of single datasets is also possible.

```
dslist <- list(data_diuron, data_diclofenac, data_naproxen)
dslist_norm <- toxprofileR::normalizeBatch(dslist = dslist)
```

Figure 8: Boxplots before and after normalization

#### Further data preprocessing

In the next step some further preprocessing is applied to the data. Measurements of duplicate probes are averaged (at the same time removing probes which are flagged of poor quality), a recent annotation of the array is added and batch correction is applied, if necessary. In our example, this was necessary for the diclofenac experiment, since the arrays were processed in two different labs.

```
data_proc_diuron <- toxprofileR::preprocess(dslist_norm[[1]], batchcorrect = F)

batch_diclofenac <- as.factor(as.numeric(dslist_norm[[2]]$targets$comment == 
    "Oaklabscan"))
data_proc_diclofenac <- toxprofileR::preprocess(dslist_norm[[2]], batchcorrect = T, 
    batch = batch_diclofenac)
```

```
## Found2batches
```

```
## Adjusting for35covariate(s) or covariate level(s)
```

```
## Standardizing Data across genes
```

```
## Fitting L/S model and finding priors
```

```
## Finding parametric adjustments
```

```
## Adjusting the Data
```

```
data_proc_naproxen <- toxprofileR::preprocess(dslist_norm[[3]], batchcorrect = F)

save(data_proc_diuron, file = "./data/diuron/data_proc_diuron.Rd")
save(data_proc_diclofenac, file = "./data/diclofenac/data_proc_diclofenac.Rd")
save(data_proc_naproxen, file = "./data/naproxen/data_proc_naproxen.Rd")
```

#### Quality control

In the end of data preprocessing we check the quality of the data with some analytical plots. We check the expression of control probes (dark corners, bright corners, spike ins) and the overall data distribution with a multidimensional scaling plot.

##### Diuron

```
toxprofileR::qc_plots(data_proc_diuron)
```

Figure 9: QC plots (after normalization) for diuron arrays

##### Diclofenac

```
toxprofileR::qc_plots(data_proc_diclofenac)
```

Figure 10: QC plots (after normalization) for diclofenac arrays

##### Naproxen

```
toxprofileR::qc_plots(data_proc_naproxen)
```

Figure 11: QC plots (after normalization) for naproxen arrays

#### Calculate logFC

In our experiment gene expression was measured at several time points. Since the embryo develops during that time there is a lot of change in gene expression due to development, we are not primarily interested in here. Therefore we normalize the expression values to the controls of each time point, therefore retrieving logFCs for each timepoint.

```
for (substance in c("diuron", "diclofenac", "naproxen")) {
    assign(paste("data", substance, "logFC", sep = "_"), value = toxprofileR::calc_logfc(get(paste0("data_proc_", 
        substance))))
    
    save(list = paste("data", substance, "logFC", sep = "_"), file = paste0("./data/", 
        substance, "/data_logfc_", substance, ".Rd"))
}
```

## Universe

### Create toxicogenomic universe

This part describes how the experimental data in combination with publicly available toxicogenomic data from the zebrafish embryo is used to infer a self-organizing map (SOM), here called the ***�toxicogenomic universe�***. The computational workflow of retrieval and normalization of public toxicogenomic datasets from Gene Expression Omnibus and ArrayExpress is published on *protocols.io* and accessible via https://dx.doi.org/10.17504/protocols.io.s24eggw.

#### Spline smooth

When using machine learning algorithms like the self-organizing map, there is no proper way of dealing with replicates and with interval data. This is why we decided to �use� this knowledge first by applying a spline-smooth on the data before feeding it into the self-organizing map. Therefore we first load the logFC data. For each gene and substance, outlier measurements are removed and afterwards a 3D-spline is fit.

```
library("toxprofileR")
for (substance in c("diuron", "diclofenac", "naproxen")) {
    
    load(file = paste0("./data/", substance, "/data_logfc_", substance, ".Rd"))
    
    assign(x = paste("data", substance, "logFC_smooth", sep = "_"), value = toxprofileR::spline_fit(get(paste("data", 
        substance, "logFC", sep = "_"))))
    
    save(list = paste("data", substance, "logFC_smooth", sep = "_"), file = paste0("./data/", 
        substance, "/data_logfc_smooth", substance, ".Rd"))
}
```

#### Load dataframe of public data

In a separate workflow we retrieved toxicogenomic data from public databases GEO and ArrayExpress. You can find the workflow for metadata retrieval, data download, annotation, normalization and processing online on https://dx.doi.org/10.17504/protocols.io.s24eggw.

```
load("./data/all/logFC_frame.Rd")
```

#### Infer self-organizing map

The logFC values which are spline-smoothed now (and do not contain any replicates any longer) are now fed into the SOM-algorithm. We save the resulting list in our project directory

```
tox_universe <- toxprofileR::create_tox_universe(dslist = list(data_diuron_logFC_smooth, 
    data_diclofenac_logFC_smooth, data_naproxen_logFC_smooth), logFC_frame = logFC_frame, 
    output = T, seed = 2312)
```

```
## create SOM
```

```
## 123473.735 sec elapsed
```

```
save(tox_universe, file = "./data/all/tox_universe.Rd")
```

#### Retrieve nodeframe

The part of the results which we will use most, is the assignment of genes to toxnodes. This is stored in the list entry `nodeframe`. We retrieve and save this as an extra file.

```
nodeframe <- tox_universe$nodeframe
save(nodeframe, file = "./data/all/universe_nodeframe.Rd")
```

#### Retrieve grid

Also often used will be the `grid`. We retrieve and save this as an extra file.

```
grid <- tox_universe$som_model$grid
save(grid, file = "./data/all/universe_grid.Rd")
```

#### Clustering

For the sake of lucidity, we add another clustering step and cluster the toxnodes by k-means clustering. First we find out, which number of clusters offers a good separation with the help of the mclust package

```
library("mclust")
set.seed(123)
d_clust_all <- Mclust(as.matrix(tox_universe$som_model$codes[[1]]), G = 10:360, 
    modelNames = c("EEI", "EII"))

plot.mclustBIC(d_clust_all$BIC)
```

We find out, that a clusternumber of 118 clusters offers a good separation for k-means clustering.

```
library("cowplot")
set.seed(123)
ztu_cluster <- kmeans(tox_universe$som_model$codes[[1]], centers = 118, iter.max = 20, 
    nstart = 10)

# plot the clustering
plotdata <- data.frame(x = tox_universe$som_model$grid$pts[, "x"], y = tox_universe$som_model$grid$pts[, 
    "y"], member = ztu_cluster$cluster)

clusterlist <- lapply(1:max(ztu_cluster$cluster), function(cluster) {
    x_median <- median(plotdata$x[plotdata$member == cluster])
    y_median <- median(plotdata$y[plotdata$member == cluster])
    return(c(x = x_median, y = y_median, cluster = cluster))
})

clusterframe <- as.data.frame(do.call("rbind", clusterlist))

clusterplot <- ggplot(plotdata, aes(x, y)) + geom_point(aes(colour = factor(member))) + 
    scale_color_manual(values = c("#b80052", "#e30066", "#ff0f69", "#ffb4bb", 
        "#a20037", "#ff305c", "#41000c", "#ff8185", "#740016", "#ff434e", "#200600", 
        "#ff614d", "#ff2124", "#ff4434", "#b6000c", "#890003", "#401100", "#d72300", 
        "#6c1e00", "#ff8b5a", "#b33b00", "#dc4b00", "#ffc6aa", "#ffaf7a", "#f47a00", 
        "#ff9c43", "#bd7600", "#452b00", "#855900", "#ffc74e", "#ffe2a3", "#d4ac00", 
        "#ffe181", "#786600", "#fff9ea", "#ffeb39", "#2d2800", "#aaaf00", "#7a8b00", 
        "#eaff5d", "#c3f700", "#517700", "#caff7a", "#192700", "#6dbe00", "#a3ff55", 
        "#173900", "#7bf42e", "#b6ff8e", "#016900", "#00c81b", "#008328", "#ccffcc", 
        "#00af41", "#00f25f", "#a6ffaf", "#001c06", "#019454", "#00361c", "#01c085", 
        "#8effd3", "#13ffd4", "#006254", "#009783", "#d8fff6", "#00352f", "#000d0b", 
        "#007f7e", "#85ffff", "#01e8ee", "#01b6be", "#006575", "#26dcff", "#03c5ef", 
        "#0299be", "#80d5ff", "#bfe7ff", "#003c52", "#22bdff", "#016293", "#279fff", 
        "#00355e", "#0069b2", "#bad1ff", "#005fb5", "#013b8b", "#b2b6ff", "#014fc6", 
        "#4f6fff", "#050021", "#00216d", "#515dff", "#000779", "#704af7", "#b48fff", 
        "#270ab6", "#200060", "#400097", "#a446f7", "#25003c", "#d172ff", "#8900ab", 
        "#f5afff", "#fbcdff", "#650067", "#ffecfd", "#d200d2", "#ff63f2", "#860076", 
        "#e500c7", "#5b0044", "#ff53c9", "#ec00b2", "#ff92d2", "#3c0029", "#ff72b5", 
        "#b3006b", "#860049")) + # geom_text(data = clusterframe,aes(label=cluster))+
labs(x = "", y = "") + theme_bw() + theme(legend.position = "none")

print(clusterplot)
```

```
# make table with clusternames
set.seed(123)
clusternames <- unique(randomNames::randomNames(500, which.names = "first"))
ztu_cluster_table <- data.frame(toxnode = 1:3600, x = tox_universe$som_model$grid$pts[, 
    "x"], y = tox_universe$som_model$grid$pts[, "y"], clustername = clusternames[ztu_cluster$cluster], 
    clusternr = ztu_cluster$cluster)
ztu_cluster_table_genes <- merge.data.frame(ztu_cluster_table, nodeframe, by = "toxnode", 
    sort = T, all = T)
save(list = c("ztu_cluster_table", "ztu_cluster_table_genes"), file = "./data/all/ztu_cluster_table.Rd")
```

#### Functional enrichment

Next, we perform a functional enrichment analysis of the clusters.

```
cluster_enrichment <- toxprofileR::enrich_clusters(cluster_table = ztu_cluster_table_genes)
save(cluster_enrichment, file = "./data/all/cluster_enrichment.Rd")
```

## CTR-model

### Regression modeling

In this part we describe how two different 3D-regression models are fit to measured logFC of toxnodes in the toxicogenomic universe. The workflow consists of

- setting parameter boundaries
- finding extrema values
- fitting regression models
- identification of significantly affected toxnodes

#### mobi-CTR model

The mobi-CTR model describes a sustained response on the concentration scale and a temporal response on the time scale. It is based on the �Hill equation�, a 3-parameter non-linear model, originally describing the binding of hemoglobin to oxygen dependent on oxygen saturation (Hill 1910). Due to its flexibility on the one hand, and physiological meaningfulness on the other hand, it was later on used in many applications (reviewed in Goutelle et al. 2008) and also proposed for pharmacological dose response modeling (Wagner 1968). One representation of the Hill-equation is shown below. It is defined by the parameters \(logFC\_{max}\), \(slope\), and \(X\_{50}\). The parameter \(logFC\_{max}\) is the maximum logarithmic fold change observed for the respective transcript, the \(slope\) defines the steepness of the curve and \(X\_{50}\) defines the concentration, where the response reaches half-maximum.

We observed that on time scale most responses showed a biphasic response. This progression can be captured by a time dependency of the parameter \(X\_{50}\). Empirically, we discovered that the dynamics of the reciprocal of \(X\_{50}\) is in many cases accurately captured by the logarithmic gaussian function. We call the reciprocal of \(X\_{50}\) �Sensitivity�, since a small \(X\_{50}\) indicates a sensitive response (i.e., the half-maximum is already reached at low concentrations). Thus, we get a full regression model describing the time and concentration dependent \(logFC\) after compound exposure: \[
\begin{align}
logFC(c) &= \dfrac{logFC\_{max}}{1+e^{-slope\*(\log(c)-\log(X\_{50}))}}\label{logFCc}\\
sensitivity(t) &= \frac{1}{X\_{50}(t)} = S\_{max}\*e^{-0.5\*(\frac{log(t)-log(t\_{max})}{S\_{dur}})^{2}}\label{Sensitivity}\\
logFC(c,t) &=\\ &logFC\_{max}/\left[1+exp\left(-slope\*(\log(c)-\right.\right.\\
&\log(1/(S\_{max}\*exp(-0.5\*(log(t)-\\
&\left.\left.log(t\_{max}))/S\_{dur})^{2})))\right)\right]+\epsilon,\\
&\epsilon \sim \mathcal{N}(0,\,\sigma^{2}),
\end{align}
\] where \(logFC\_{max}\) corresponds to the maximum fold change of the respective gene across all conditions, \(S\_{max}\) is the maximum sensitivity (\(1/EC\_{50}\)) of the gene, \(t\_{max}\) is the time-point of maximum sensitivity, and \(S\_{dur}\) represents a measure of duration of the sensitivity interval.

#### Parameter boundaries

In a global parameter optimization method like *shuffled complex evolution*, we need to carefully define the parameter boundaries. They will be dependent from the range of measured concentrations (\(concrange\) = highest - lowest measured concentration) and the dilution factor (\(DF\)), as well as from the measured time points.

##### Smax

The sensitivity can be below or above tested concentrations. It will be not discriminable from the data, how far it is above or below. Therefore \(S\_{max}\) should be limited to a small range above and below the tested concentration range.

###### minimum

\[\frac{1}{concrange\*max(\text{appl. concentration})}\]

###### maximum

\[\frac{concrange}{min(\text{appl. concentration})}\]

##### slope

###### maximum

The *maximum* slope we can unravel depends on the spacing of our concentrations. It would describe a change of effect from approximately 1% to 99% in between two measured concentrations. This implies an effect of 99% in the \(X\_{50}\) multiplied by the square root of the dilution factor (DF):

\[
\begin{align}
&&Effect &= \dfrac{logFC\_{max}}{1+\exp{\left(-slope\*(\log{c}-\log{X\_{50}})\right)}}&&\\
\Leftrightarrow && &= \dfrac{logFC\_{max}}{1+\exp{\left(\log{\left(c^{-slope}\right)}-\log{\left(X\_{50}^{-slope}\right)}\right)}}&&\\
\Leftrightarrow && &= \dfrac{logFC\_{max}}{1+\exp{\left(\log{\left(\frac{c^{-slope}}{X\_{50}^{-slope}}\right)}\right)}}&&\\
\Leftrightarrow && &= \dfrac{logFC\_{max}}{1+\left(\frac{c}{X\_{50}}\right)^{-slope}}&&\text{insert values}\\
&&0.99\*logFC\_{max}&=\dfrac{logFC\_{max}}{1+\left(\frac{X\_{50}\*\sqrt{DF}}{X\_{50}}\right)^{-slope}}&&\\
\Leftrightarrow && \sqrt{DF}^{-slope} &= \frac{1}{0.99}-1\\
\Leftrightarrow && slope &= -\dfrac{\log{\left(\frac{1}{0.99}-1\right)}}{\log{\left(\sqrt{DF}\right)}}
\end{align}
\]

###### minimum

For the minimum slope the calculation is similar. Here we expect, that a minimum slope that still can be unraveled by our measurements would describe a change of 1% effect across the measured concentration range. This implies an effect of 50.5% at a concentration of \(EC\_{50}\) multiplied by the square root of the concentration range. Therefore the minimum slope is defined by: \[-\frac{\log{(\frac{1}{0.505}-1)}}{\log{\sqrt{concrange}}}\]

##### \(S\_{dur}\)

###### minimum

The lowest \(S\_{dur}\) we can measure depends on the spacing of our time points. If an effect occurs only at one time point, there should be less than 1% effect at the preceding/succeeding measured time points. In our setting the smallest factor between two time points is 2/3 (between 48 and 72h):

\[
\begin{align}
&&Sensitivity &= S\_{max}\*\exp{\left(-0.5\*\left(\frac{\log{t}-\log{t\_{max}}}{S\_{dur}}\right)^2\right)}\\
&&0.01\*S\_{max}&=S\_{max}\*\exp{\left(-0.5\*\left(\frac{\log{2/3}}{S\_{dur}}\right)^2\right)}\\
\Leftrightarrow && \dfrac{\log{(2/3)}}{S\_{dur}} &= \sqrt{-2\*\log{0.01}}\\
\Leftrightarrow && S\_{dur} &= \dfrac{\log{(2/3)}}{\sqrt{-2\*\log{0.01}}}
\end{align}
\] This implies that the term \(\exp{(-0.5\*(\frac{log (48/72)}{S\_{dur}})^2)}\), which describes the time dependence of the sensitivity should be 0.01.

###### maximum

\[\frac{\log{72/3}}{\sqrt{-2\*\log{0.99}}}\]

##### \(t\_{max}\)

The highest (measured) sensitivity of a gene/toxnode can only be in the range of measured time points. The limits are set a bit wider to avoid boundary effects in model fitting.

###### minimum

1.5

###### maximum

75

#### Extrema

To get an estimate for the paramter \(logFC\_{max}\) in the CTR-model, we load the logFC data of all datasets and find extreme values. For each node the maximum and minimum \(logFC\) across all given experimental treatments is retrieved.

```
library("toxprofileR")

for (substance in c("diuron", "diclofenac", "naproxen")) {
    
    load(file = paste0("./data/", substance, "/data_logfc_", substance, ".Rd"))
    
}

dslist <- list(data_diuron_logFC, data_diclofenac_logFC, data_naproxen_logFC)

load(file = "./data/all/universe_nodeframe.Rd")

extrema <- toxprofileR::get_extrema_nodes(dslist = dslist, nodeframe = nodeframe)

save(extrema, file = "./data/all/node_extrema.Rd")
```

#### Fitting

Parameter estimation is performed using the *shuffled complex evolution* algorithm implemented in the `hydromad` package. It is based on the Nelder-Mead-Algorithm, but includes the generation and shuffling of several simplices generated from random points across the parameter space. This makes the algorithm robust and independent from starting values, which is why we selected it for parameter estimation of our toxnode models.

```
library("cowplot")

for (substance in c("diuron", "diclofenac", "naproxen")) {
    
    tcta_paramframe_som <- toxprofileR::fit_tcta(elist = get(paste("data", substance, 
        "logFC", sep = "_")), extrema = extrema, nodeframe = nodeframe, cluster = F)
    
    p1 <- ggplot(data = data.frame(AICweight = c(unlist(tcta_paramframe_som$AICw_up_hill)[unlist(tcta_paramframe_som$direction_hill == 
        1)], unlist(tcta_paramframe_som$AICw_down_hill)[unlist(tcta_paramframe_som$direction_hill == 
        -1)])), aes(AICweight)) + geom_histogram(col = "black", alpha = 0.2, 
        binwidth = 0.1) + xlab("AIC weight compared to null model") + theme(axis.text = element_text(size = 24), 
        axis.title = element_text(size = 28))
    
    p2 <- ggplot(data = data.frame(AICweight = unlist(tcta_paramframe_som$AICw_vspline_hill)), 
        aes(AICweight)) + geom_histogram(col = "black", alpha = 0.2, binwidth = 0.1) + 
        xlab("AIC weight compared to spline") + theme(axis.text = element_text(size = 24), 
        axis.title = element_text(size = 28))
    
    print(p1)
    print(p2)
    
    assign(x = paste0("tcta_paramframe_som_", substance), value = tcta_paramframe_som)
    save(list = paste0("tcta_paramframe_som_", substance), file = paste0("./data/", 
        substance, "/tcta_", substance, ".Rd"))
}
```

```
## 77221.75 sec elapsed
```

```
## 68325.344 sec elapsed
```

```
## 60162.049 sec elapsed
```

#### Significance

Finally, we can use the fitted model to identify significantly regulated toxnodes.

##### Diuron

```
library("toxprofileR")
load("./data/all/universe_nodeframe.Rd")

substance <- "diuron"


load(file = paste0("./data/", substance, "/data_logfc_", substance, ".Rd"))
load(file = paste0("./data/", substance, "/tcta_", substance, ".Rd"))
nodelist <- toxprofileR::create_nodelist(elist = get(paste("data", substance, 
    "logFC", sep = "_")), nodeframe = nodeframe)

# get CI diffs
assign(paste0("CI_diffs_", substance), toxprofileR::get_CI_diffs(tcta_frame = get(paste0("tcta_paramframe_som_", 
    substance)), nodelist = nodelist))

# get significance level
assign(paste0("sig_level", substance), toxprofileR::get_sig_level(CIdiffs = get(paste0("CI_diffs_", 
    substance)), model = "hill-gauss", siglevel = 0))

# save
save(list = c(paste0("CI_diffs_", substance), paste0("sig_level", substance)), 
    file = paste0("./data/", substance, "/CI_diff_", substance, ".Rd"))

n_up <- sum(get(paste0("sig_level", substance)) > 0)
n_down <- sum(get(paste0("sig_level", substance)) < 0)

message(paste("there are", n_up, "toxnodes up-regulated,\n and", n_down, "toxnodes down-regulated\n by", 
    substance, "\n\n"))
```

```
## there are 32 toxnodes up-regulated,
##  and 28 toxnodes down-regulated
##  by diuron
```

```
top10 <- data.frame(toxnode = order(abs(get(paste0("sig_level", substance))), 
    decreasing = T)[1:10])

top10$probe_ids <- unlist(lapply(top10$toxnode, function(toxnode) {
    paste(nodeframe$ProbeID[nodeframe$toxnode == toxnode & !is.na(nodeframe$ProbeID)], 
        collapse = ", ")
}))

annotation <- get(paste0("data_", substance, "_logFC"))$genes
top10$genenames <- unlist(lapply(top10$toxnode, function(toxnode) {
    paste(unique(annotation$external_gene_name[annotation$ProbeName %in% nodeframe$ProbeID[nodeframe$toxnode == 
        toxnode & !is.na(nodeframe$ProbeID)]]), collapse = ", ")
}))

assign(paste0("top10", substance), top10)
top10
```

##### Diclofenac

```
library("toxprofileR")
load("./data/all/universe_nodeframe.Rd")

substance <- "diclofenac"


load(file = paste0("./data/", substance, "/data_logfc_", substance, ".Rd"))
load(file = paste0("./data/", substance, "/tcta_", substance, ".Rd"))
nodelist <- toxprofileR::create_nodelist(elist = get(paste("data", substance, 
    "logFC", sep = "_")), nodeframe = nodeframe)

# get CI diffs
assign(paste0("CI_diffs_", substance), toxprofileR::get_CI_diffs(tcta_frame = get(paste0("tcta_paramframe_som_", 
    substance)), nodelist = nodelist))

# get significance level
assign(paste0("sig_level", substance), toxprofileR::get_sig_level(CIdiffs = get(paste0("CI_diffs_", 
    substance)), model = "hill-gauss", siglevel = 0))

# save
save(list = c(paste0("CI_diffs_", substance), paste0("sig_level", substance)), 
    file = paste0("./data/", substance, "/CI_diff_", substance, ".Rd"))

n_up <- sum(get(paste0("sig_level", substance)) > 0)
n_down <- sum(get(paste0("sig_level", substance)) < 0)

message(paste("there are", n_up, "toxnodes up-regulated,\n and", n_down, "toxnodes down-regulated\n by", 
    substance, "\n\n"))
```

```
## there are 53 toxnodes up-regulated,
##  and 20 toxnodes down-regulated
##  by diclofenac
```

```
top10 <- data.frame(toxnode = order(abs(get(paste0("sig_level", substance))), 
    decreasing = T)[1:10])

top10$probe_ids <- unlist(lapply(top10$toxnode, function(toxnode) {
    paste(nodeframe$ProbeID[nodeframe$toxnode == toxnode & !is.na(nodeframe$ProbeID)], 
        collapse = ", ")
}))

annotation <- get(paste0("data_", substance, "_logFC"))$genes
top10$genenames <- unlist(lapply(top10$toxnode, function(toxnode) {
    paste(unique(annotation$external_gene_name[annotation$ProbeName %in% nodeframe$ProbeID[nodeframe$toxnode == 
        toxnode & !is.na(nodeframe$ProbeID)]]), collapse = ", ")
}))

assign(paste0("top10", substance), top10)
top10
```

##### Naproxen

```
library("toxprofileR")
load("./data/all/universe_nodeframe.Rd")

substance <- "naproxen"


load(file = paste0("./data/", substance, "/data_logfc_", substance, ".Rd"))
load(file = paste0("./data/", substance, "/tcta_", substance, ".Rd"))
nodelist <- toxprofileR::create_nodelist(elist = get(paste("data", substance, 
    "logFC", sep = "_")), nodeframe = nodeframe)

# get CI diffs
assign(paste0("CI_diffs_", substance), toxprofileR::get_CI_diffs(tcta_frame = get(paste0("tcta_paramframe_som_", 
    substance)), nodelist = nodelist))

# get significance level
assign(paste0("sig_level", substance), toxprofileR::get_sig_level(CIdiffs = get(paste0("CI_diffs_", 
    substance)), model = "hill-gauss", siglevel = 0))

# save
save(list = c(paste0("CI_diffs_", substance), paste0("sig_level", substance)), 
    file = paste0("./data/", substance, "/CI_diff_", substance, ".Rd"))

n_up <- sum(get(paste0("sig_level", substance)) > 0)
n_down <- sum(get(paste0("sig_level", substance)) < 0)

message(paste("there are", n_up, "toxnodes up-regulated,\n and", n_down, "toxnodes down-regulated\n by", 
    substance, "\n\n"))
```

```
## there are 202 toxnodes up-regulated,
##  and 151 toxnodes down-regulated
##  by naproxen
```

```
top10 <- data.frame(toxnode = order(abs(get(paste0("sig_level", substance))), 
    decreasing = T)[1:10])

top10$probe_ids <- unlist(lapply(top10$toxnode, function(toxnode) {
    paste(nodeframe$ProbeID[nodeframe$toxnode == toxnode & !is.na(nodeframe$ProbeID)], 
        collapse = ", ")
}))

annotation <- get(paste0("data_", substance, "_logFC"))$genes
top10$genenames <- unlist(lapply(top10$toxnode, function(toxnode) {
    paste(unique(annotation$external_gene_name[annotation$ProbeName %in% nodeframe$ProbeID[nodeframe$toxnode == 
        toxnode & !is.na(nodeframe$ProbeID)]]), collapse = ", ")
}))

assign(paste0("top10", substance), top10)
top10
```

## Info

### Session Info

|  | x |
| --- | --- |
| version | R version 3.4.3 (2017-11-30) |
| system | x86\_64, linux-gnu |
| ui | X11 |
| language | (EN) |
| collate | en\_US.UTF-8 |
| tz | NA |
| date | 2019-01-11 |

| package | \* | version | date | source |
| --- | --- | --- | --- | --- |
| abind |  | 1.4-5 | 2016-07-21 | CRAN (R 3.4.3) |
| acepack |  | 1.4.1 | 2016-10-29 | CRAN (R 3.4.3) |
| AnnotationDbi | \* | 1.40.0 | 2018-02-14 | Bioconductor |
| assertthat |  | 0.2.0 | 2017-04-11 | CRAN (R 3.4.3) |
| backports |  | 1.1.2 | 2017-12-13 | CRAN (R 3.4.3) |
| base | \* | 3.4.3 | 2018-02-14 | local |
| base64enc |  | 0.1-3 | 2015-07-28 | CRAN (R 3.4.3) |
| bindr |  | 0.1.1 | 2018-03-13 | cran (@0.1.1) |
| bindrcpp |  | 0.2.2 | 2018-03-29 | cran (@0.2.2) |
| Biobase | \* | 2.38.0 | 2018-02-14 | Bioconductor |
| BiocGenerics | \* | 0.24.0 | 2018-08-07 | Bioconductor |
| bit |  | 1.1-12 | 2014-04-09 | CRAN (R 3.4.3) |
| bit64 |  | 0.9-7 | 2017-05-08 | CRAN (R 3.4.3) |
| bitops |  | 1.0-6 | 2013-08-17 | CRAN (R 3.4.3) |
| blob |  | 1.1.0 | 2017-06-17 | CRAN (R 3.4.3) |
| captioner | \* | 2.2.3.9000 | 2018-10-11 | Github (adletaw/captioner@5f2b435) |
| car |  | 3.0-2 | 2018-08-23 | cran (@3.0-2) |
| carData |  | 3.0-1 | 2018-03-28 | cran (@3.0-1) |
| cellranger |  | 1.1.0 | 2016-07-27 | cran (@1.1.0) |
| checkmate |  | 1.8.5 | 2017-10-24 | CRAN (R 3.4.3) |
| cluster |  | 2.0.6 | 2017-03-10 | CRAN (R 3.4.3) |
| colorspace |  | 1.3-2 | 2016-12-14 | CRAN (R 3.4.3) |
| compiler |  | 3.4.3 | 2018-02-14 | local |
| cowplot | \* | 0.9.4 | 2019-01-08 | cran (@0.9.4) |
| curl |  | 3.3 | 2019-01-10 | cran (@3.3) |
| data.table |  | 1.11.8 | 2018-09-30 | cran (@1.11.8) |
| datasets | \* | 3.4.3 | 2018-02-14 | local |
| DBI |  | 1.0.0 | 2018-05-02 | CRAN (R 3.4.3) |
| devtools |  | 1.13.4 | 2017-11-09 | CRAN (R 3.4.3) |
| digest |  | 0.6.18 | 2018-10-10 | cran (@0.6.18) |
| dplyr |  | 0.7.8 | 2018-11-10 | cran (@0.7.8) |
| evaluate |  | 0.10.1 | 2017-06-24 | CRAN (R 3.4.3) |
| forcats |  | 0.3.0 | 2018-02-19 | cran (@0.3.0) |
| foreign |  | 0.8-69 | 2017-06-22 | CRAN (R 3.4.3) |
| Formula |  | 1.2-2 | 2017-07-10 | CRAN (R 3.4.3) |
| GenomeInfoDb |  | 1.14.0 | 2018-08-07 | Bioconductor |
| GenomeInfoDbData |  | 1.0.0 | 2018-08-07 | Bioconductor |
| GenomicRanges |  | 1.30.3 | 2018-08-07 | Bioconductor |
| ggplot2 | \* | 3.1.0 | 2018-10-25 | cran (@3.1.0) |
| glue |  | 1.3.0 | 2018-07-17 | cran (@1.3.0) |
| graphics | \* | 3.4.3 | 2018-02-14 | local |
| grDevices | \* | 3.4.3 | 2018-02-14 | local |
| grid |  | 3.4.3 | 2018-02-14 | local |
| gridExtra |  | 2.3 | 2017-09-09 | CRAN (R 3.4.3) |
| gtable |  | 0.2.0 | 2016-02-26 | CRAN (R 3.4.3) |
| haven |  | 1.1.2 | 2018-06-27 | cran (@1.1.2) |
| Hmisc |  | 4.1-1 | 2018-01-03 | CRAN (R 3.4.3) |
| hms |  | 0.4.2 | 2018-03-10 | cran (@0.4.2) |
| htmlTable |  | 1.11.2 | 2018-01-20 | CRAN (R 3.4.3) |
| htmltools |  | 0.3.6 | 2017-04-28 | CRAN (R 3.4.3) |
| htmlwidgets |  | 1.3 | 2018-09-30 | cran (@1.3) |
| httr |  | 1.4.0 | 2018-12-11 | cran (@1.4.0) |
| hydromad | \* | 0.9-15 | 2019-01-11 | Github (floybix/hydromad@7cd2898) |
| IRanges | \* | 2.12.0 | 2018-02-14 | Bioconductor |
| jsonlite |  | 1.6 | 2018-12-07 | cran (@1.6) |
| knitr |  | 1.19 | 2018-01-29 | CRAN (R 3.4.3) |
| lattice | \* | 0.20-35 | 2017-03-25 | CRAN (R 3.4.3) |
| latticeExtra | \* | 0.6-28 | 2016-02-09 | CRAN (R 3.4.3) |
| lazyeval |  | 0.2.1 | 2017-10-29 | CRAN (R 3.4.3) |
| limma | \* | 3.34.9 | 2018-06-27 | Bioconductor |
| magrittr |  | 1.5 | 2014-11-22 | CRAN (R 3.4.3) |
| Matrix |  | 1.2-12 | 2017-11-20 | CRAN (R 3.4.3) |
| mclust | \* | 5.4.1 | 2018-06-27 | CRAN (R 3.4.3) |
| memoise |  | 1.1.0 | 2017-04-21 | CRAN (R 3.4.3) |
| methods | \* | 3.4.3 | 2018-02-14 | local |
| mgcv | \* | 1.8-22 | 2017-09-24 | CRAN (R 3.4.3) |
| munsell |  | 0.5.0 | 2018-06-12 | cran (@0.5.0) |
| nlme | \* | 3.1-131 | 2017-02-06 | CRAN (R 3.4.3) |
| nnet |  | 7.3-12 | 2016-02-02 | CRAN (R 3.4.3) |
| openxlsx |  | 4.1.0 | 2018-05-26 | cran (@4.1.0) |
| org.Dr.eg.db | \* | 3.5.0 | 2018-11-23 | Bioconductor |
| outliers | \* | 0.14 | 2011-01-24 | CRAN (R 3.4.3) |
| parallel | \* | 3.4.3 | 2018-02-14 | local |
| pbapply | \* | 1.3-4 | 2018-01-10 | cran (@1.3-4) |
| pillar |  | 1.1.0 | 2018-01-14 | CRAN (R 3.4.3) |
| pkgconfig |  | 2.0.2 | 2018-08-16 | cran (@2.0.2) |
| plotly |  | 4.8.0 | 2018-07-20 | cran (@4.8.0) |
| plyr |  | 1.8.4 | 2016-06-08 | CRAN (R 3.4.3) |
| polynom | \* | 1.3-9 | 2016-12-08 | cran (@1.3-9) |
| purrr |  | 0.2.5 | 2018-05-29 | cran (@0.2.5) |
| R6 |  | 2.3.0 | 2018-10-04 | cran (@2.3.0) |
| RColorBrewer | \* | 1.1-2 | 2014-12-07 | CRAN (R 3.4.3) |
| Rcpp |  | 1.0.0 | 2018-11-07 | cran (@1.0.0) |
| RCurl |  | 1.95-4.10 | 2018-01-04 | CRAN (R 3.4.3) |
| readxl |  | 1.1.0 | 2018-04-20 | cran (@1.1.0) |
| rio |  | 0.5.10 | 2018-03-29 | cran (@0.5.10) |
| rlang |  | 0.3.0.1 | 2018-10-25 | cran (@0.3.0.1) |
| rmarkdown |  | 1.8 | 2017-11-17 | CRAN (R 3.4.3) |
| rpart |  | 4.1-11 | 2017-03-13 | CRAN (R 3.4.3) |
| rprojroot |  | 1.3-2 | 2018-01-03 | CRAN (R 3.4.3) |
| RSQLite |  | 2.0 | 2017-06-19 | CRAN (R 3.4.3) |
| rstudioapi |  | 0.7 | 2017-09-07 | CRAN (R 3.4.3) |
| S4Vectors | \* | 0.16.0 | 2018-02-14 | Bioconductor |
| scales |  | 1.0.0 | 2018-08-09 | cran (@1.0.0) |
| snow | \* | 0.4-2 | 2016-10-14 | CRAN (R 3.4.3) |
| splines |  | 3.4.3 | 2018-02-14 | local |
| stats | \* | 3.4.3 | 2018-02-14 | local |
| stats4 | \* | 3.4.3 | 2018-02-14 | local |
| stringi |  | 1.2.4 | 2018-07-20 | cran (@1.2.4) |
| stringr |  | 1.3.1 | 2018-05-10 | cran (@1.3.1) |
| survival |  | 2.41-3 | 2017-04-04 | CRAN (R 3.4.3) |
| tibble |  | 1.4.2 | 2018-01-22 | CRAN (R 3.4.3) |
| tidyr |  | 0.8.2 | 2018-10-28 | cran (@0.8.2) |
| tidyselect |  | 0.2.5 | 2018-10-11 | cran (@0.2.5) |
| tools |  | 3.4.3 | 2018-02-14 | local |
| toxprofileR | \* | 0.3.0 | 2019-01-11 | git (@e234eed) |
| utils | \* | 3.4.3 | 2018-02-14 | local |
| viridisLite |  | 0.3.0 | 2018-02-01 | CRAN (R 3.4.3) |
| withr |  | 2.1.2 | 2018-03-15 | cran (@2.1.2) |
| XVector |  | 0.18.0 | 2018-08-07 | Bioconductor |
| yaml |  | 2.2.0 | 2018-07-25 | cran (@2.2.0) |
| zip |  | 1.0.0 | 2017-04-25 | cran (@1.0.0) |
| zlibbioc |  | 1.24.0 | 2018-06-27 | Bioconductor |
| zoo | \* | 1.8-4 | 2018-09-19 | cran (@1.8-4) |

## References

Andrews, F.T., B.F.W. Croke, and A.J. Jakeman. 2011. �An Open Software Environment for Hydrological Model Assessment and Development.� *Environmental Modelling & Software* 26 (10): 1171�85. doi:10/fqf4mp.

Durinck, Steffen, Paul T. Spellman, Ewan Birney, and Wolfgang Huber. 2009. �Mapping Identifiers for the Integration of Genomic Datasets with the R/Bioconductor Package biomaRt.� *Nature Protocols* 4 (8): 1184�91. doi:10/c4b7dd.

Goutelle, Sylvain, Michel Maurin, Florent Rougier, Xavier Barbaut, Laurent Bourguignon, Michel Ducher, and Pascal Maire. 2008. �The Hill Equation: A Review of Its Capabilities in Pharmacological Modelling.� *Fundamental & Clinical Pharmacology* 22 (6): 633�48. doi:10.1111/j.1472-8206.2008.00633.x.

Hill, Archibald Vivian. 1910. �The Possible Effects of the Aggregation of the Molecules of Haemoglobin on Its Dissociation Curves.� *The Journal of Physiology* 40: i�vii.

Ritchie, Matthew E., Belinda Phipson, Di Wu, Yifang Hu, Charity W. Law, Wei Shi, and Gordon K. Smyth. 2015. �Limma Powers Differential Expression Analyses for RNA-Sequencing and Microarray Studies.� *Nucleic Acids Research* 43 (7): e47�e47. doi:10/f7c4n5.

Scholze, Martin, Wolfgang Boedeker, Michael Faust, Thomas Backhaus, Rolf Altenburger, and L. Horst Grimme. 2001. �A General Best-Fit Method for Concentration-Response Curves and the Estimation of Low-Effect Concentrations.� *Environmental Toxicology and Chemistry* 20 (2): 448�57. doi:10/fbnfr8.

Wagner, J.G. 1968. �Kinetics of Pharmacologic Response I. Proposed Relationships Between Response and Drug Concentration in the Intact Animal and Man.� *Journal of Theoretical Biology* 20 (2): 173�201. doi:10/bxg4h8.

Wehrens, Ron, and Lutgarde M. C. Buydens. 2007. �Self- and Super-Organizing Maps in *R* : The **Kohonen** Package.� *Journal of Statistical Software* 21 (5). doi:10/gfc74p.

Xie, Yihui. 2017. *Dynamic Documents with R and Knitr*. Boca Raton, Florida: CRC Press. https://proquest.safaribooksonline.com/9781315360706.
