# Supplementary material for: Map and model—moving from observation to prediction in toxicogenomics
Source: Gigascience. 2019 May 29;8(6):giz057. doi: 10.1093/gigascience/giz057 (PMC6539241; doi:10.1093/gigascience/giz057)
Supplement: giz057_Response_to_Reviewer_Comments_Original_Submission [file giz057_response_to_reviewer_comments_original_submission.pdf]

Author's Response To Reviewer Comments

Close

We thank the editor and the three reviewers for their constructive and valuable remarks.
We addressed the suggested revisions in our manuscript and supply specific point-by-point answers below.

##
Response to editor:

“please register any new software application in the SciCrunch.org database to receive a RRID (Research Resource Identification Initiative ID) number, and include this in your manuscript. This will facilitate tracking, reproducibility and re-use of your tool.”

Response: Agree - We have registered toxprofileR and the Toxicogenomic Fingerprint Browser at the SciCrunch.org database and added the RRIDs in the manuscript.

Changes made in the manuscript:
Added RRIDs in the section "Availability of source code and requirements".

##
Responses to reviewer #1:
1.1 "Some of the technical implementation details could be potentially omitted in the scientific reporting part, in order to make it easier to follow. However, I would not request changes to the work regarding this."

Response: We acknowledge the technical heaviness of our study and double checked the scientific reporting part for technical details that could be omitted in favor of readability. However, we did not find any parts which would be dispensable in our view.

Changes made in the manuscript:
none

1.2 "Adding the Rmd source files in the supplements should be sufficient for transparency"

Response: Agree

Changes made in the manuscript:
.Rmd source files were added together with a source file for the plots and tables in a .zip file in the supplementary data and referenced in the supporting data section

----
1.3 “If it possible, I would propose switching from GPL to MIT license”

Response: We completely agree with the reviewer, that a more permissive licence would be desirable. However the work is partly based on GPL licensed packages, why this option is unfortunately not available.

Changes made in the manuscript:
none

##
Responses to reviewer #2:

2.1 "One suggestion would be to make the type of information provided in the supplemental methods more explicit in the manuscript methods section."

Response: Agree

Changes made in the manuscript:
Addition to the Methods part: "[We additionally prepared an extensive supplementary methods file] (supplementary_methods.html). This file contains [detailed information about the experimental procedure] (exposure, RNA extraction, measurement of toxicokinetics) [and the data analysis pipeline] (data import, quality control, creating the toxicogenomic universe, regression modelling)[...]

2.2 "I believe that it is difficult to adequately control for the amount of variability in the publically available transcriptomic datasets, which is why the limitations of the analysis need to be clearly stated. Major limitations of the bioinformatic analysis strategy described include the lack of consideration of other factors that may cause variation in transcriptomic results. Zebrafish embryos are sensitive to environmental factors, such as incubation temperature, and in toxicological studies, the time exposure is initiated is thought to be important. Based on the description of methods, length of exposure was taken into consideration, but time of initial exposure in the integrated studies seems not to have been accounted for. As reported in the manuscript many developmental toxicity assays start exposure immediately after fertilization, although 4-6 hours post fertilization is also commonly seen. The exposures in this study start at 24 hours post fertilization, which may mean that early gene expression changes were missed due to the later dosing period. The difference in dosing time also makes it difficult to compare across post exposure time points, because embryos may be different at different stages of development."

Response: Clarification - We completely agree with the reviewer that there can be many different factors (incubation temperature, exposure start and duration, pH, light conditions, genetic background, etc.) which may influence the transcriptome profiles of zebrafish embryos to different degree. Some of these factors do differ substantially between the experiments (which we discussed in detail in Schüttler et al. 2017). In the presented approach, however, these factors should not influence the analysis. This is because we use fold-change data which is normalized to the control of the respective experiment. Moreover, we did not compare different studies here, but used the normalized gene expression information for clustering genes within the self-organizing-map approach. Certainly, more data and data from experiments with different settings, chemicals, etc. would potentially influence the result of this clustering. We tried to clarify this in the manuscript. Additionally, we acknowledged in the discussion that co-expression may also differ between different treatments.

Changes made in the manuscript:
Addition to Data analysis part: "All datasets were normalised against the respective control of the same experiment"
Addition to Discussion: "With the help of the SOM we can retrieve information about co-expression of genes from publicly available toxicogenomic datasets and include this information into our analysis irrespective of differing experimental designs and occurrence of missing data."
Addition to Discussion: "Additionally, co-expression of genes is not necessarily consistent across different perturbations [68]. This would not be captured in the dynamic toxicogenomic fingerprints as shown here."

2.3 "The authors describe an incubation temperature of 26 degrees Celsius for this study, but 28.5 degrees Celsius is a more common incubation temperature."

Response: We agree that incubation temperature may influence the development of the zebrafish embryos. However, we mainly expect the fish to develop slower in this temperature range, which should not influence the found toxicogenomic responses. In our study we chose an incubation temperature of 26 degrees celsius as it is used in OECD guideline No. 236 (OECD, 2013).

OECD (2013), Test No. 236: Fish Embryo Acute Toxicity (FET) Test, OECD Guidelines for the Testing of Chemicals, Section 2, OECD Publishing, Paris, https://doi.org/10.1787/9789264203709-en.

Changes made in the manuscript:
none

2.4 "It should also be noted that the diuron exposure medium contained 0.1% methanol, and it does not appear that a 0.1% methanol solvent control was used in addition to a water control."

Response: Clarification - We clarified in the supplemental methods file, where we describe details of the exposure, that we indeed used solvent controls for the diuron exposure.

Changes made in the manuscript:
Addition in the supplemental methods file: "For the diuron exposure we used 0.1 % methanol as solvent control."

2.5
"Although the manuscript does provide very detailed descriptions of design and on the analysis of data from the case study, the limitations of the model are not well addressed."
"[...]more discussion is needed into the limitations of the model"
"As mentioned previously, I think the manuscript needs to better address limitations. Currently, the analysis and discussion are slightly on the overly positive side and need to be balanced."
"a weakness is the possible overgeneralization of different experimental design or environmental conditions associated with zebrafish embryos (incubation temperature, time exposure is initiated)"

Response:
Agree

Changes made in the manuscript:
i) Addition in discussion (Integration and aggregation of toxicogenomic responses): "A SOM shows limitations in capturing complex interactions compared to the more 'flexible' networks in the aforementioned studies (i.e. connections between nodes are only formed between direct neighbors on the map). Therefore some relevant interactions between transcriptomic units might get lost with the SOM. Additionally, co-expression of genes is not necessarily consistent across different perturbations [68]. This would not be captured in the dynamic toxicogenomic fingerprints as shown here. However,SOMs have the advantage of [...]"
ii) Addition in discussion (Modelling of time and concentration dependence): "For example, toxnodes showing a biphasic response on the concentration scale would not be accurately captured".
iii) Addition in discussion (Modelling of time and concentration dependence): "So far the regression model describes responses for a specific exposure setting. Other experimental settings may require refining the model."

2.6 "I would also like to see further discussion on some of the points in the Summary and Implications section. The "Dynamic toxicogenomic fingerprints for read-across and elucidation of adverse outcome pathway(s)" and "Molecular mixture toxicology" subsections could be further expanded: One of my questions from the "Dynamics" subsection is how can the existing Universe be used to evaluate a novel compound"

Response: Agree (also compare comment 3.2)

Changes made in the manuscript:
Addition to Implications: "For this we can use previously derived functional knowledge about responding toxnodes or clusters to draw conclusions about the effects of other substances, as exemplarily shown in Figure S8."

Addition to Discussion: "The supplementary methods file (supplementary_methods.html) demonstrates how to use our supplied R-package toxprofileR and the toxicogenomic universe to infer toxicogenomic fingerprints of additional compounds."

2.7 "The "Molecular mixture" subsection lacks any discussion of interactions such as synergy, additive effects, or antagonistic effects. This section needs further discussion; alternatively, it would not hurt the manuscript if it were removed."

Response: Agree

Changes made in the manuscript:
Addition to Implications: "Existing mixture concepts such as concentration addition or independent action could guide the evaluation of mixture responses and the identification of additive or non-additive behaviour [109]"

2.8 "I also wonder about how well a reduced array with only one gene from each toxnode would perform and if it would have any utility. I could see reduced arrays within toxnodes being more useful for interrogation of specific

mechanisms within a general effect."

Response: Clarification - We agree that the utility of reduced, comprehensive transcriptome arrays has to be proven. However, we observe an increased development and evaluation of reduced transcriptome arrays. We propose that the toxicogenomic universe could be helpful in the array design. We acknowledge the reviewers idea of reduced arrays withing toxnodes or clusters and added this to the manuscript.

Changes in the manuscript:
Addition to Implications: "[Therefore, an alternative approach to design a reduced zebrafish array could comprise the selection of a representative gene for each toxnode of the Zebrafish Embryo Toxicogenomic Universe (ZTU)] or the selection of genes within a specific region of interest in the ZTU."

2.9 "[...] it should be noted that no internal standard or spike in was used in the measurement of diuron, diclofenac, or naproxen in zebrafish embryo/larval tissues and it is not mentioned if the concentration of drug was confirmed in the exposure media"

"The toxicokinetics portion also is weaker than it could be due to only direct analysis of compounds only without accounting for internal or spiked controls or for active metabolites. These weaknesses should be acknowledged"

Response: Agree and clarification - we did not use a spike-in for calibration and clarified this now in the supplementary methods. We confirmed the concentration of drug in the exposure media and added this information to the supplementary methods file now.

Changes in the manuscript:
Addition in supplementary methods file (section toxicokinetics): "A calibration curve was acquired in the respective medium. No additional spike-in or internal standard was used for calibration."
Added plot with measured concentration in exposure medium to supplementary methods file.

##
Responses to reviewer #3:

3.1 "The generation of the toxicogenomic universe utilizes only microarrays, which is a predetermined set of probes. I realize the concept is like the Connectivity Maps from the L1000 platform, but how will this platform be expanded upon later in the future? The technology of microarray is slowly growing out of favor and no new probes will be added with the discovery of new genes. This should be discussed in the paper to explain what the next extension will be"

Response: We agree with the reviewer that the microarray technology may phase out eventually. However, our approach is independent of the technology used to measure the transcriptome. We added a statement in the discussion to clarify this.

Changes made in the manuscript:
Addition in Discussion: "While in our study the analysis focused on microarray data, the approach is not limited to microarray data and could also be applied on RNAseq data. This is also demonstrated in Figure S8B showing RNAseq data projected on the toxicogenomic universe."

3.2 "The toxicogenomic universe is provided with little explanation of how someone can adapt all this great work. It would be a disservice to not include an excerpt of how the field of toxicology can use it."

Response: Agree (compare comment 2.6). The R-package supplied with the paper should allow adaption of the toxicogenomic universe and the described methods for future studies. The supplementary methods file can be seen as a manual how to use this. We added a statement in the discussion to clarify this.

Changes made in the manuscript:
Addition to discussion: The supplementary methods file (supplementary_methods.html) demonstrates how to use our supplied R-package \textit{toxprofileR} and the toxicogenomic universe to infer toxicogenomic fingerprints."

3.3 "The shiny app is great to display your own results for the 3 chemicals, which has concentration and time data. However, how will it work with chemicals that are tested at only one concentration or one time point? This is a limitation should be explained."

Response: Clarification - As it is demonstrated in Figure S8 (also see comment 3.2) the toxicogenomic universe can also be used to project profiles of only one concentration or time point. With the help of the toxprofileR package time- and concentration dependent data can be processed to be viewed with the toxicogenomic fingerprint browser.

Changes made in the manuscript:
none

3.4 "However, how does this work if there non-monotonic responses? The chemical selected are known to have responses that are dose dependent, but not every chemical is this case. A discussion should be included on this."

Response: Agree

Changes made in the manuscript (see also Comment 2.5):
Addition in discussion (Modelling of time and concentration dependence): "For example, toxnodes showing a biphasic response on the concentration scale would not be accurately captured".

3.5 "AIC is used for the selection of the model - which is appropriate. Please provide some information on the range of AIC values. Also - was there a likelihood test conducted?"

Response: Clarification - Because AIC is only a relative measure of model fit for a given dataset, the absolute value itself is meaningless (Akaike, 1981). AIC values are only useful for comparing different models fitted one one dataset. We selected the models using AIC, which is the likelihood of a model penalized by its complexity. This also allowed us to compare non-nested models, which cannot be done using a Likelihood Ratio test. For these reasons, we chose not to conduct Likelihood Ratio tests. The range of relative AIC-weights is given in Figure S4.

Akaike, Hirotugu. (1981). Likelihood of a model and information criteria. Journal of Econometrics, 16(1), 3-14. doi: http://dx.doi.org/10.1016/0304-4076(81)90071-3

Changes in the manuscript: none

3.6 "Figure 8 is to small to see anything meaningful. The discussion around it is non-descript and hard to follow"

Response: Agree and Clarification - Figure 8 is not meant to show details of a fingerprint. We rewrote the discussion around Figure 8 to make this more clear.4

Changes in the manuscript:

Updated Figure 8

Changed paragraph discussing Figure 8: "As it is not conceivable that concentrations, selected and measured in experiments, will ever cover the whole range of concentrations relevant for estimating or interpreting environmental effects, measures for extrapolation become relevant. In this regard, the CTR-model allows building hypotheses and predictions about toxicogenomic effects at conditions not measured. For example, with the help of the model we can predict for each toxnode in the ZTU which response we would expect for an environmentally relevant concentration of 0.86 µmol/L of diuron [concentration measured in 75]. This is falling outside the concentration range measured in our study. In Figure 8 the expected logFCs for 16 and 80 hpe are projected on the ZTU. Without investigating the profiles in detail, we can observe that the predictions show distinctly different profiles. A prominently down-regulated cluster can be seen in the early time point (blue spot bottom left) and a prominently up-regulated cluster in the late time point (red spot upper left). This demonstrates once more that one substance may induce distinctly different effect profiles depending on time and concentration. Furthermore, if such profiles were indeed measured in an environmental sample, we would only be able to link these profiles to a diuron exposure with dynamic and

concentration-dependent information as supplied by the CTR-model.

3.7 "In the paragraph: "To obtain an overview over the ZTU we grouped the 3600 toxnodes into 118 clusters", please include information on the range of nodes per cluster"

Response: Agree

Changes in the manuscript:
Addition to results part: "The resulting clusters contained between 3 and 93 toxnodes with an average of 30."

3.8 "Figure 3- the size of the dot isn't even visible. This should be removed, as there is no point to this."

Response: Agree

Changes in the manuscript:
Changed figure accordingly

3.9 "modeling section - In regards to the first sentence - "consideration of finding specific" - that does this refer to and needs more clarification."

Response: Agree

Changes in the manuscript:
Changed sentence to "The analysis up to this step allows the consideration of findings for each exposure setting in isolation."

3.10 "Figure 6 legend - clarify "sum(CI)""

Response: Agree

Changes in the manuscript:
Added “Dotsize represents significant effect level (sumCI, compare Figure S5A).” to figure legend.

Close
