# Supplementary material for: Map and model—moving from observation to prediction in toxicogenomics
Source: Gigascience. 2019 May 29;8(6):giz057. doi: 10.1093/gigascience/giz057 (PMC6539241; doi:10.1093/gigascience/giz057)
Supplement: giz057_Response_to_Reviewer_Comments_Revision-1 [file giz057_response_to_reviewer_comments_revision-1.pdf]

Author's Response To Reviewer Comments

Close

We thank the reviewers once more for their constructive and valuable remarks and the scientific discussion about our manuscript.
We addressed the suggested revisions in our manuscript and supply specific point-by-point answers below.

##
Responses to editor:

"And as you have carried out some wet lab experiments, GigaScience encourages and assists with the submission of detailed protocols to the open access repository protocols.io. Please enter the details into protocols.io , issue a DOI, and cite the protocols.io record from the Methods section."

Response: We agree, that new methods and workflows should be published at protocols.io. We did this for the protocol of data retrieval for the ZTU as mentioned in the methods section. All wet lab experiments were performed according to standard protocols with minor modifications (such as the OECD test guideline for the fish embryo toxicity test (OECD TG 236) and the Agilent microarray protocol which we have cited accordingly). We do not see any advantage of re-publishing these standard protocols.
Changes made in the manuscript: none

##
Responses to reviewer #2:

2.1: "Previously, I requested a more explicit explanation of the limitations of the described model with respect to variation in experimental design factors of the zebrafish transcriptomic datasets. I disagree with the authors' statement about how using fold change data normalized to respective experimental controls would prevent factors such as time chemical exposure was initiated and incubation temperature from influencing the analysis. The dynamic nature of gene expression during development makes these factors critical, as genes are normally up- and downregulated during development and chemical exposure during different periods can cause different gene expression profiles. Likewise, difference in incubation temperatures would change the 'length' of those developmental windows and would influence microarray results. Comparing normalized fold change data would not adequately compare for these differences, as the controls would be different.

The authors cited in their clarification the Schüttler et al. (2017) manuscript. This article does a good job about discussing the limitations of comparing methods in zebrafish embryo transcriptomic studies. I think the current manuscript would benefit from a larger discussion of the previous article or otherwise a greater discussion of the problems associated with comparing across differently designed studies." [...]

"I can agree with the assumption that a slower development would not influence the toxicogenomic responses within a single study. I do think it limits comparisons of multiple studies at different incubation temperatures because the response to a chemical over time would not proceed at the same rate, due to the difference in rate of development. This can be addressed along with the previous comment, but I do think this is an important limitation of the study that needs to be mentioned in greater detail."

Response: Agree and Clarification - We agree with the reviewer about the limitations when comparing toxicogenomic effects across different experimental designs, which is essential to consider, but often neglected. Indeed, we did not intend to compare results of studies with different time windows, incubation temperatures, etc., but only used their findings to retrieve information about co-expression (which might be the only useful information to derive from these snapshot experiments). We understand that this distinction has not been made sufficiently clear in the manuscript and therefore added another paragraph for explanation.

Changes made in the manuscript:
Addition to discussion: "With the help of the SOM we can retrieve information about co-expression of genes from publicly available toxicogenomic datasets and include this information into our analysis. This way we can make use of this heterogeneous data and gain information for clustering the genes irrespective of differing experimental

designs and occurrence of missing data. While the projection of toxicogenomic fingerprints on the ZTU increases accessibility and comparability of past findings (compare Figure S8) one should be aware of the limitations of such comparisons due to differences in experimental factors as it was discussed in Schüttler et al. [18]. Here, we show how the application of regression models could foster comparisons between studies with different exposure concentrations and time frames (see below)."

2.2: "The comment, 'Additionally, co-expression of genes is not necessarily consistent across different perturbations [68]. This would not be captured in the dynamic toxicogenomic fingerprints as shown here.' needs to be expanded and more explicit."

Response: Agree - We added an example to make this statement more explicit.

Changes made in the manuscript: "For example, when a compound binds to a transcription factor and thereby modulates its activity, co-expression with its target genes will significantly decrease."

2.3: "The authors added to the manuscript the statement, "For example, toxnodes showing a biphasic response on the concentration scale would not be accurately captured." I think this is an important point and think some examples would be beneficial. For example, endocrine disrupting compounds typically have non-monotonic dose responses and a statement to this effect could be added."

Response: Agree

Changes made in the manuscript: "Biphasic responses of gene expression have been reported to commonly occur in response to chemical exposure [22]. Especially the activation of steroid hormone receptors favors a biphasic response in gene expression [74]. This might limit the applicability of our model for endocrine acting substances."

##

Response to reviewer #3:

The authors have adequately addressed most of my concerns. I understand that the values of AIC is relative, however, the methods section should provide enough information if one is to build this model again, that they would be able to obtain the same results. If this information is buried in the supplemental figures, that is inappropriate. The detail should be moved into the detailed methods.

Response: We are not sure if we understand the comment correctly here. The distribution of AIC weights are discussed and interpreted in the main part of the manuscript. Reviewer #1 recommended to rather leave out some technical details in the main part. Therefore we refrain from moving Figure S4 to the main part.

Changes made in the manuscript: none

Close
