# Supplementary material for: Map and model—moving from observation to prediction in toxicogenomics
Source: Gigascience. 2019 May 29;8(6):giz057. doi: 10.1093/gigascience/giz057 (PMC6539241; doi:10.1093/gigascience/giz057)
Supplement: giz057_Reviewer_1_Report_Original_Submission -- Leo Lahti — 2/27/2019 Reviewed [file giz057_reviewer_1_report_original_submission.pdf]

Reviewer Report

Title: Map and Model - moving from observation to prediction in toxicogenomics

Version: Original Submission **Date:** 2/27/2019

Reviewer name: Leo Lahti

Reviewer Comments to Author:

I have been requested to specifically address methodological aspects and reproducibility of "Map and Model - moving from observation to prediction in toxicogenomics". Therefore I limit the review on those aspects.

This work is very comprehensive, and it proposes, implements, and demonstrates the use of a toxicogenomic fingerprint database that has been constructed by combining publicly available gene expression data sets with self-organizing maps. It is demonstrated how this can guide the interpretation of new experiments. More specifically, transcriptomic profiles can be compared to the toxicogenomic map in order to detect toxicogenomic fingerprints in the samples. Various aspects of such analysis are being discussed, including dose-response and temporal effects with different treatments, and a web server application is provided to demonstrate the approach. The use of the method is demonstrated but the overall scope of this work is in designing the method. Value for applications remains to be proven but would be clearly out of scope of this methodologically oriented manuscript.

The manuscript is well written in fluent English. It is easy to read in terms of English language and I do not see a need for editing in this regard. The work is technically rather involved, though, and hence not a light read. This is also making it difficult to fully evaluate all technical aspects and details of the manuscript. Overall, the work appears to be sound. I have been able to assess all statistics in the manuscript to a sufficient degree, including the appropriateness of statistical tests. To my best understanding of this technically heavy work, the conclusions are adequately supported by the data.

The methods are appropriate to the aims of the study and well described. The robustness of the model has been evaluated sufficiently. Many technical details could be further benchmarked and improved but such comparisons would be clearly out of scope for this study. The availability of supplementary materials, in particular the web server application and HTML reports, is an advantage but at the same time, some of the technical implementation details could be potentially omitted in the scientific reporting part, in order to make it easier to follow. However, I would not request changes to the work regarding this.

I was also asked to evaluate the reproducibility. I could install the R package and access the data files and supplementary documentation (HTML). I did not find the original Rmarkdown files, however, which would be necessary to replicate the experiments. Therefore, although source code, data sets and documentation are available as separate but not readily executable files, I have to conclude that I could not reproduce the experiments based on the available material. This being said, it seems to me that the experiments have been carried out rigorously. Adding the Rmd source files in the supplements should be sufficient for transparency.

The chosen software license appears to be GPL3. This viral license is more restrictive than some other

open licenses, in particular the non-viral MIT license, that have been recommended for research software (see DOI:10.1371/journal.pcbi.1002598). If it possible, I would propose switching from GPL to MIT license. This will improve the usability of the work by others.

Methods

Are the methods appropriate to the aims of the study, are they well described, and are necessary controls included? Choose an item.

Conclusions

Are the conclusions adequately supported by the data shown? Choose an item.

Reporting Standards

Does the manuscript adhere to the journal's guidelines on [minimum standards of reporting?]() Choose an item.

Choose an item.

Statistics

Are you able to assess all statistics in the manuscript, including the appropriateness of statistical tests used? Choose an item.

Quality of Written English

Please indicate the quality of language in the manuscript: Choose an item.

Declaration of Competing Interests

Please complete a declaration of competing interests, considering the following questions:

- Have you in the past five years received reimbursements, fees, funding, or salary from an organisation that may in any way gain or lose financially from the publication of this manuscript, either now or in the future?
- Do you hold any stocks or shares in an organisation that may in any way gain or lose financially from the publication of this manuscript, either now or in the future?
- Do you hold or are you currently applying for any patents relating to the content of the manuscript?
- Have you received reimbursements, fees, funding, or salary from an organization that holds or has applied for patents relating to the content of the manuscript?
- Do you have any other financial competing interests?
- Do you have any non-financial competing interests in relation to this paper?

If you can answer no to all of the above, write 'I declare that I have no competing interests' below. If your reply is yes to any, please give details below.

I declare that I have no competing interests.

I agree to the open peer review policy of the journal. I understand that my name will be included on my report to the authors and, if the manuscript is accepted for publication, my named report including any attachments I upload will be posted on the website along with the authors' responses. I agree for my report to be made available under an Open Access Creative Commons CC-BY license (http://creativecommons.org/licenses/by/4.0/). I understand that any comments which I do not wish to be included in my named report can be included as confidential comments to the editors, which will not be published.

I agree to the open peer review policy of the journal

To further support our reviewers, we have joined with Publons, where you can gain additional credit to further highlight your hard work (see: https://publons.com/journal/530/gigascience). On publication of this paper, your review will be automatically added to Publons, you can then choose whether or not to claim your Publons credit. I understand this statement.

Yes Choose an item.
