# Supplementary material for: Map and model—moving from observation to prediction in toxicogenomics
Source: Gigascience. 2019 May 29;8(6):giz057. doi: 10.1093/gigascience/giz057 (PMC6539241; doi:10.1093/gigascience/giz057)
Supplement: giz057_Reviewer_2_Report_Original_Submission -- Katharine Horzmann, DVM, Ph.D., MPH — 2/27/2019 Reviewed [file giz057_reviewer_2_report_original_submission.pdf]

Reviewer Report

Title: Map and Model - moving from observation to prediction in toxicogenomics

Version: Original Submission **Date:** 2/27/2019

Reviewer name: Katharine Horzmann, DVM, Ph.D., MPH

Reviewer Comments to Author:

The submitted manuscript, "Map and Model - moving from observational to prediction in toxicogenomics" is a well written article that describes the creation of a self-organizing map to integrate known transcriptomic datasets into a "Zebrafish Embryo Toxicogenomic Universe", the aggregation of compound data into toxicogenomic fingerprints accounting for both time and concentration, and the utility of using the Zebrafish Embryo Toxicogenomic University to predict responses. The manuscript nicely fits the aims and scope of the journal and is sufficiently transparent with respect to data sources, coding, and results. All links to data and sources within the manuscript are active and working. The manuscript is the first, to my knowledge, to integrate both time and concentration into the bioinformatics analysis framework, which is a significant advancement. Although the manuscript does provide very detailed descriptions of design and on the analysis of data from the case study, the limitations of the model are not well addressed. The limitations and some of the Summary and Implication points should be expanded.

1. Is the rationale for collecting and analyzing the data well defined?

The rationale behind the development of a Universe of concentration and time dependent toxicogenomic fingerprints is very solid.

The work collects a number of datasets into an appropriately large-scale integration of transcriptomic responses.

The methods used in identification of published datasets and the methods for microarrays used to test the model are well described in the manuscript and supplemental methods material.

The description of the experimental design and the potential uses are adequately described, although more discussion is needed into the limitations of the model.

The study is well justified by previous work and properly makes use of references.

2. Is it clear how data was collected and curated?

The methods for data collection are well defined and all data and supporting information is easily accessed through links provided in the manuscript.

3. Is it clear - and was a statement provided - on how data and analyses tools used in the study can be accessed?

The methods for data analysis are well defined and all supporting information is easily accessed through links provided in the manuscript. Once suggestion would be to make the type of information provided in the supplemental methods more explicit in the manuscript methods section.

4. Are accession numbers given or links provided for data that, as a standard, should be submitted to a community approved public repository?

Yes, GEO accession numbers are provided for the previously published datasets used for the creation of

the Zebrafish Embryo Toxicogenomic Universe and for the array datasets from the case study presented in the manuscript.
5. Is the data and software available in the public domain under a Creative Commons license?
The GEO accession numbers are provided for the previously published transcriptomic datasets and for the microarrays used in the study. R code, analysis methods, and a results interactive webpage are publically available.
6. Are the data sound and well controlled?
I believe that it is difficult to adequately control for the amount of variability in the publically available transcriptomic datasets, which is why the limitations of the analysis need to be clearly stated. Major limitations of the bioinformatic analysis strategy described include the lack of consideration of other factors that may cause variation in transcriptomic results. Zebrafish embryos are sensitive to environmental factors, such as incubation temperature, and in toxicological studies, the time exposure is initiated is thought to be important. Based on the description of methods, length of exposure was taken into consideration, but time of initial exposure in the integrated studies seems not to have been accounted for. As reported in the manuscript many developmental toxicity assays start exposure immediately after fertilization, although 4-6 hours post fertilization is also commonly seen. The exposures in this study start at 24 hours post fertilization, which may mean that early gene expression changes were missed due to the later dosing period. The difference in dosing time also makes it difficult to compare across post exposure time points, because embryos may be different at different stages of development. This is further complicated by incubation temperature. The authors describe an incubation temperature of 26 degrees Celsius for this study, but 28.5 degrees Celsius is a more common incubation temperature. Again, development stage, and thus normal gene expression may be different between embryos the same age reared in different conditions. Another complication would be difference in controls across studies, as sometimes DMSO, ethanol, or methanol is used to solubilize chemicals for exposure, but some studies are not adequately controlled with both a water control and a solvent control.It should also be noted that the diuron exposure medium contained 0.1% methanol, and it does not appear that a 0.1% methanol solvent control was used in addition to a water control.
The numbers of embryos per treatment group seem low, although I think the type and robustness of the analysis compensates somewhat for this.
7. Is the interpretation (Analysis and Discussion) well balanced and supported by the data?
The interpretation of the Data Analyses and Results and the Discussion sections is appropriate. The sections are well organized and the authors logically report and interpret the data in a manner that is mostly balanced and well supported by references. As mentioned previously, I think the manuscript needs to better address limitations. Currently, the analysis and discussion are slightly on the overly positive side and need to be balanced.
I would also like to see further discussion on some of the points in the Summary and Implications section. The "Dynamic toxicogenomic fingerprints for read-across and elucidation of adverse outcome pathway(s)" and "Molecular mixture toxicology" subsections could be further expanded. One of my questions from the "Dynamics" subsection is how can the existing Universe be used to evaluate a novel compound? The "Molecular mixture" subsection lacks any discussion of interactions such as synergy, additive effects, or antagonistic effects. This section needs further discussion; alternatively, it would not hurt the manuscript if it were removed.

I also wonder about how well a reduced array with only one gene from each toxnode would perform and if it would have any utility. I could see reduced arrays within toxnodes being more useful for interrogation of specific mechanisms within a general effect.
8. Are the methods appropriate, well described, and include sufficient details and supporting information to allow others to evaluate and replicate the work?
The methods seem appropriate, although it should be noted that no internal standard or spike in was used in the measurement of diuron, diclofenac, or naproxen in zebrafish embryo/larval tissues and it is not mentioned if the concentration of drug was confirmed in the exposure media. Otherwise the methods described for the microarray, data processing, and modeling/analysis seem appropriate, well described, and accessible.
9. What are the strengths and weaknesses of the methods?
The most intriguing aspect of the methods is the incorporation of concentration and time into the model. However, as mentioned previously, a weakness is the possible overgeneralization of different experimental design or environmental conditions associated with zebrafish embryos (incubation temperature, time exposure is initiated). The toxicokinetics portion also is weaker than it could be due to only direct analysis of compounds only without accounting for internal or spiked controls or for active metabolites. These weaknesses should be acknowledged.
10. Have the authors followed best-practices in reporting standards?
The authors appear to have followed best-practices in reporting standards.
11. Can the writing, organization, tables and figures be improved?
The manuscript is well organized, and seems to appropriately use tables and figures of sufficient quality.

Methods

Are the methods appropriate to the aims of the study, are they well described, and are necessary controls included? Choose an item.

Conclusions

Are the conclusions adequately supported by the data shown? Choose an item.

Reporting Standards

Does the manuscript adhere to the journal's guidelines on minimum standards of reporting? Choose an item.

Choose an item.

Statistics

Are you able to assess all statistics in the manuscript, including the appropriateness of statistical tests used? Choose an item.

Quality of Written English

Please indicate the quality of language in the manuscript: Choose an item.

Declaration of Competing Interests

Please complete a declaration of competing interests, considering the following questions:

- Have you in the past five years received reimbursements, fees, funding, or salary from an organisation that may in any way gain or lose financially from the publication of this manuscript, either now or in the future?
- Do you hold any stocks or shares in an organisation that may in any way gain or lose financially from the publication of this manuscript, either now or in the future?
- Do you hold or are you currently applying for any patents relating to the content of the manuscript?
- Have you received reimbursements, fees, funding, or salary from an organization that holds or has applied for patents relating to the content of the manuscript?
- Do you have any other financial competing interests?
- Do you have any non-financial competing interests in relation to this paper?

If you can answer no to all of the above, write 'I declare that I have no competing interests' below. If your reply is yes to any, please give details below.

I declare that I have no competing interest.

I agree to the open peer review policy of the journal. I understand that my name will be included on my report to the authors and, if the manuscript is accepted for publication, my named report including any attachments I upload will be posted on the website along with the authors' responses. I agree for my report to be made available under an Open Access Creative Commons CC-BY license (http://creativecommons.org/licenses/by/4.0/). I understand that any comments which I do not wish to be included in my named report can be included as confidential comments to the editors, which will not be published.

I agree to the open peer review policy of the journal

To further support our reviewers, we have joined with Publons, where you can gain additional credit to further highlight your hard work (see: https://publons.com/journal/530/gigascience). On publication of this paper, your review will be automatically added to Publons, you can then choose whether or not to claim your Publons credit. I understand this statement.

Yes Choose an item.
