# Supplementary material for: Map and model—moving from observation to prediction in toxicogenomics
Source: Gigascience. 2019 May 29;8(6):giz057. doi: 10.1093/gigascience/giz057 (PMC6539241; doi:10.1093/gigascience/giz057)
Supplement: giz057_Reviewer_2_Report_Revision_1 -- Katharine Horzmann, DVM, Ph.D., MPH — 3/24/2019 Reviewed [file giz057_reviewer_2_report_revision_1.pdf]

Reviewer Report

Title: Map and Model - moving from observation to prediction in toxicogenomics

Version: Revision 1 **Date:** 3/24/2019

Reviewer name: Katharine Horzmann, DVM, Ph.D., MPH

Reviewer Comments to Author:

This is the second review of the submitted manuscript, "Map and Model - moving from observation to prediction in toxicogenomics". Overall, the manuscript is still well written and describes a very interesting model. The changes the authors have made to the manuscript are generally favorable and help provide additional information to clarify reviewer concerns or provide additional information. However, some reviewer comments could be further addressed.

The points below refer to the numbered sections in the Author's Response to Review's Comments.

Response 2.2: Previously, I requested a more explicit explanation of the limitations of the described model with respect to variation in experimental design factors of the zebrafish transcriptomic datasets. I disagree with the authors' statement about how using fold change data normalized to respective experimental controls would prevent factors such as time chemical exposure was initiated and incubation temperature from influencing the analysis. The dynamic nature of gene expression during development makes these factors critical, as genes are normally up- and downregulated during development and chemical exposure during different periods can cause different gene expression profiles. Likewise, difference in incubation temperatures would change the 'length' of those developmental windows and would influence microarray results. Comparing normalized fold change data would not adequately compare for these differences, as the controls would be different.

The authors cited in their clarification the SchÃ¼ttler et al. (2017) manuscript. This article does a good job about discussing the limitations of comparing methods in zebrafish embryo transcriptomic studies. I think the current manuscript would benefit from a larger discussion of the previous article or otherwise a greater discussion of the problems associated with comparing across differently designed studies. The comment, "Additionally, co-expression of genes is not necessarily consistent across different perturbations [68]. This would not be captured in the dynamic toxicogenomic fingerprints as shown here." needs to be expanded and more explicit.

Response 2.3: I can agree with the assumption that a slower development would not influence the toxicogenomic responses within a single study. I do think it limits comparisons of multiple studies at different incubation temperatures because the response to a chemical over time would not proceed at the same rate, due to the difference in rate of development. This can be addressed along with the previous comment, but I do think this is an important limitation of the study that needs to be mentioned in greater detail.

Response 3.4: The authors added to the manuscript the statement, "For example, toxnodes showing a biphasic response on the concentration scale would not be accurately captured." I think this is an important point and think some examples would be beneficial. For example, endocrine disrupting compounds typically have non-monotonic dose responses and a statement to this effect could be

added.
I really think it is important to highlight limitations, not to diminish the accomplishments of the research, but to add to its practicality and self-awareness.

Methods

Are the methods appropriate to the aims of the study, are they well described, and are necessary controls included? Choose an item.

Conclusions

Are the conclusions adequately supported by the data shown? Choose an item.

Reporting Standards

Does the manuscript adhere to the journal's guidelines on minimum standards of reporting? Choose an item.

Choose an item.

Statistics

Are you able to assess all statistics in the manuscript, including the appropriateness of statistical tests used? Choose an item.

Quality of Written English

Please indicate the quality of language in the manuscript: Choose an item.

Declaration of Competing Interests

Please complete a declaration of competing interests, considering the following questions:

- Have you in the past five years received reimbursements, fees, funding, or salary from an organisation that may in any way gain or lose financially from the publication of this manuscript, either now or in the future?
- Do you hold any stocks or shares in an organisation that may in any way gain or lose financially from the publication of this manuscript, either now or in the future?
- Do you hold or are you currently applying for any patents relating to the content of the manuscript?
- Have you received reimbursements, fees, funding, or salary from an organization that holds or has applied for patents relating to the content of the manuscript?
- Do you have any other financial competing interests?
- Do you have any non-financial competing interests in relation to this paper?

If you can answer no to all of the above, write 'I declare that I have no competing interests' below. If your reply is yes to any, please give details below.

I declare that I have no competing interests.

I agree to the open peer review policy of the journal. I understand that my name will be included on my report to the authors and, if the manuscript is accepted for publication, my named report including any attachments I upload will be posted on the website along with the authors' responses. I agree for my report to be made available under an Open Access Creative Commons CC-BY license (http://creativecommons.org/licenses/by/4.0/). I understand that any comments which I do not wish to be included in my named report can be included as confidential comments to the editors, which will not be published.

I agree to the open peer review policy of the journal

To further support our reviewers, we have joined with Publons, where you can gain additional credit to further highlight your hard work (see: https://publons.com/journal/530/gigascience). On publication of this paper, your review will be automatically added to Publons, you can then choose whether or not to claim your Publons credit. I understand this statement.

Yes Choose an item.
