# Supplementary material for: Map and model—moving from observation to prediction in toxicogenomics
Source: Gigascience. 2019 May 29;8(6):giz057. doi: 10.1093/gigascience/giz057 (PMC6539241; doi:10.1093/gigascience/giz057)
Supplement: giz057_Reviewer_3_Report_Original_Submission -- Lisa Truong — 3/3/2019 Reviewed [file giz057_reviewer_3_report_original_submission.pdf]

Reviewer Report

Title: Map and Model - moving from observation to prediction in toxicogenomics

Version: Original Submission **Date:** 3/3/2019

Reviewer name: Lisa Truong

Reviewer Comments to Author:

aThe manuscript "Map and Model - moving from observation to prediction in toxicogenomics" is well written and describes a bioinformatics approach to infer time and concentration toxicogenomic fingerprints. They utilize developing zebrafish and assessed their transcriptome response when exposed to diuron, diclofenac, and naproxen and measure the internal concentrations. The authors does a great describing the mathematics and their experimental design, however there are a few points that should be considered/discussed about in the manuscript as it is:

1. The generation of the toxicogenomic universe utilizes only microarrays, which is a predetermined set of probes. I realize the concept is like the Connectivity Maps from the L1000 platform, but how will this platform be expanded upon later in the future? The technology of microarray is slowly growing out of favor and no new probes will be added with the discovery of new genes. This should be discussed in the paper to explain what the next extension will be
2. The prediction ability of toxicogenomics is briefly discussed but its not clear how this will work for future of the field. The authors are able to model the mass amount of data, but its not clear how others in the field will utilize their data set. The toxicogenomic universe is provided with little explanation of how someone can adapt all this great work. It would be a disservice to not include an excerpt of how the field of toxicology can use it.
3. The shiny app is great to display your own results for the 3 chemicals, which has concentration and time data. However, how will it work with chemicals that are tested at only one concentration or one time point? This is a limitation should be explained.
4. The model used for the concentration and time-dependent response model is a good choice with the assumption that all the data fits under the hill model. However, how does this work if there non-monotonic responses? The chemical selected are known to have responses that are dose dependent, but not every chemical is this case. A discussion should be included on this. Some data might be better fit with a gain-loss model. Or constant model if the internal dose is non-existent (because of chemical 'soprtion properties).
5. AIC is used for the selectin of the model - which is appropriate. Please provide some information on the range of AIC values. Also - was there a likelihood test conducted?
6. Figure 8 is to small to see anything meaningful. The discussion around it is non-descript and hard to follow.

Minor clarification:

Pg 4 - "integration: the Zebrafish Embryo Toxicogenomic.." - In the paragraph: "To obtain an overview over the ZTU we grouped the 3600 toxnodes into 118 clusters", please include information on the range of nodes per cluster

Figure 3- the size of the dot isn't even visible. This should be removed, as there is no point to this. Also - the figure is specific to Naproxen so the concentrations should be stated on the plot.
Pg 5 - modeling section - In regards to the first sentence - "consideration of finding specific" - that does this refer to and needs more clarification.
Figure 6 legend - clarify "sum(CI)".

Methods

Are the methods appropriate to the aims of the study, are they well described, and are necessary controls included? Choose an item.

Conclusions

Are the conclusions adequately supported by the data shown? Choose an item.

Reporting Standards

Does the manuscript adhere to the journal's guidelines on [minimum standards of reporting?]() Choose an item.

Choose an item.

Statistics

Are you able to assess all statistics in the manuscript, including the appropriateness of statistical tests used? Choose an item.

Quality of Written English

Please indicate the quality of language in the manuscript: Choose an item.

Declaration of Competing Interests

Please complete a declaration of competing interests, considering the following questions:

- Have you in the past five years received reimbursements, fees, funding, or salary from an organisation that may in any way gain or lose financially from the publication of this manuscript, either now or in the future?
- Do you hold any stocks or shares in an organisation that may in any way gain or lose financially from the publication of this manuscript, either now or in the future?
- Do you hold or are you currently applying for any patents relating to the content of the manuscript?
- Have you received reimbursements, fees, funding, or salary from an organization that holds or has applied for patents relating to the content of the manuscript?
- Do you have any other financial competing interests?

- Do you have any non-financial competing interests in relation to this paper?

If you can answer no to all of the above, write 'I declare that I have no competing interests' below. If your reply is yes to any, please give details below.

I declare that I have no competing interests

I agree to the open peer review policy of the journal. I understand that my name will be included on my report to the authors and, if the manuscript is accepted for publication, my named report including any attachments I upload will be posted on the website along with the authors' responses. I agree for my report to be made available under an Open Access Creative Commons CC-BY license (http://creativecommons.org/licenses/by/4.0/). I understand that any comments which I do not wish to be included in my named report can be included as confidential comments to the editors, which will not be published.

I agree to the open peer review policy of the journal

To further support our reviewers, we have joined with Publons, where you can gain additional credit to further highlight your hard work (see: https://publons.com/journal/530/gigascience). On publication of this paper, your review will be automatically added to Publons, you can then choose whether or not to claim your Publons credit. I understand this statement.

Yes Choose an item.
